# Supplementary material for: Intimate partner violence against women with disability and associated mental health concerns: a cross-sectional survey in Mumbai, India
Source: BMJ Open. 2022 Apr 26;12(4):e056475. doi: 10.1136/bmjopen-2021-056475 (PMC9047698; doi:10.1136/bmjopen-2021-056475)
Supplement: Supplementary data [file bmjopen-2021-056475supp001.pdf]

Text (English)	Type	Text (Hindi)
****What is your religion?****		**आपका धर्म क्या है?**
Hindu	Choice	हिंदू
Muslim	Choice	मुस्लिम
Christian	Choice	ईसाई
Sikh	Choice	सिख
Buddhist/Neo-Buddhist	Choice	बौद्ध / नव-बौद्ध
Jain	Choice	जैन
Parsi/Zoroastrian	Choice	पारसी / पारसी
No religion/did not say	Choice	कोई धर्म नहीं/जानकारी नहीं
****What is your caste?****		**आपकी जाति क्या हैं?**
Open/General	Choice	सामान्य
OBC	Choice	ओबीसी
Scheduled caste (SC)	Choice	अनुसूचित जाति
Scheduled tribe (ST)	Choice	अनुसूचित जनजाति
None of these	Choice	इनमें से कोई भी नहीं
**What kind of house do you live in?**		**आप किस तरह के घर में रहतें हैं?**
Kachha (non-concrete)	Choice	कच्चा
Pucca (concrete)	Choice	पक्का
Mixed concrete and non-concrete	Choice	कच्चा एवं पक्का, दोनों
Other	Choice	अन्य
**own?**		आपका अपना घर है?**
Own	Choice	स्वयं का
Rented	Choice	किराये का
Other	Choice	अन्य
**What type of toilet facility do you use?**		**आप किस प्रकार के शौचालय सुविधा का प्रयोग करते हैं?**
Private or individual	Choice	निजी
Public	Choice	सार्वजानिक
Paid toilet	Choice	निजी लेकिन भुगतान शौचालय
Charity toilet	Choice	सार्वजानिक लेकिन भुगतान सौचालय

Open defecation	Choice	खुले में
Other	Choice	अन्य
*****Now I would like to ask you about some important aspects of a woman's life. You may find some of the questions personal. However, your answers are crucial for helping us understand the condition of women in the community. Let me assure you that your answers will be completely confidential and no one else in your household will know that you were asked. If I ask you any question you don't want to answer, just let me know and I will go on to the next question.*****		***अब मैं आपसे महिलाओं के जीवन के कुछ महत्वपूर्ण पहलुओं के बारे में सवाल पूछना चाहती हूँ. इनमें से कुछ प्रश्न व्यक्तिगत हो सकते हैं. इन जानकारी से बस्ती में महिलाओं की स्थिति को और अच्छे से समझने में के लिए आपके उत्तर महत्वपूर्ण हैं. मैं आपको भरोसा देती हूँ कि आपके उत्तर पूरी तरह से गोपनीय रखे जायेंगे और उन्हें किसी को भी बताया नहीं जाएगा, और आपके परिवार में से भी कोई भी नहीं जान पायेगा कि आपसे ये सरे सवाल पूछे गए हैं. यदि आप मेरे द्वारा पूछे गये किसी भी सवाल का जवाब नहीं चाहतीं हैं कृपया मुझे बताये, मै आगे के सवालों पर बढ़ जाउंगी.***
****What is your age?****	Integer	**आपकी (उत्तरदाता) की उम्र कितनी उम्र है?**
****What is your current marital status?****		**वर्तमान में आपकी (उत्तरदाता) वैवाहिक स्थिति क्या है?**
Unmarried	Choice	अविवाहित
Married, Gauna not performed	Choice	शादी की पर गौना नहीं हुआ
Currently married	Choice	शादीशुदा
Living with partner	Choice	साथी के साथ रहना
Separated	Choice	पति से अलग
Divorced	Choice	तलाकशुदा
Widowed/Widower	Choice	विधवा/ विधुर
Answer refused	Choice	उत्तर से इनकार कर दिया
**completed?****		**आपकी उच्चतम शिक्षा क्या है?**
No formal education	Choice	कोई औपचारिक शिक्षा नहीं
Primary (1-5th standard)	Choice	प्राइमरी (1-5th standard)
Middle (6-8th standard)	Choice	मिडिल (6-8th standard)
High school (9-10th standard)	Choice	हाई स्कूल (9-10th standard)
Senior school (11-12th standard)	Choice	सीनियर स्कूल (11-12th standard)
Undergraduate	Choice	ग्रेजुएशन पूरा नहीं
Graduate	Choice	ग्रेजुएशन

Incomplete postgraduate	Choice	पोस्ट ग्रेजुएशन पूरा नहीं
Postgraduate or higher	Choice	पोस्ट ग्रेजुएशन और आगे
Other	Choice	अन्य
**WG-SS**		**विकलांगता**
difficulty. Please reply which describes your condition appropriately.***		प्रकार की कठिनाई है तो, कृपया उत्तर दें जो कि आपकी स्थिति का उचित रूप से वर्णन करता हो.***
**Do you have difficulty seeing, even if wearing glasses?**		*(कृपया पूंछे कि क्या वे चश्मा पहनने पहनते है? यदि हाँ तो क्या चश्मा पहनने के बाद भी उन्हें देखने में परेशानी होती है क्या?)*
No - no difficulty	Choice	नहीं देखने में कोई कठिनाई नहीं
Yes – some difficulty	Choice	हां - देखने में कुछ कठिनाई
Yes – a lot of difficulty	Choice	हां - देखने में बहुत मुश्किल
Cannot do at all	Choice	बिल्कुल नहीं देख सकते
hearing aid?**		उपयोग करते है)?**
**Do you have difficulty walking or climbing steps?**		**क्या आपको चलने या चढ़ने में कठिनाई होती है?**
concentrating?**		**क्या आपको चीजे याद रखने में या ध्यान केंद्रित करने में कठिनाई होती है?**
washing all over or dressing?**		है?**
difficulty communicating, for example, understanding or being understood?**		कठिनाई होती है, उदाहरण के लिए, दूसरों की बातों को समझ पाने में या खुद की बातों को दूसरों को समझाने में?**
**Respondent's livelihood**		**आजीविका**
they are paid in cash or kind. Others sell things, have a small business or work on the family farm or in the family business. In the last 12 months, have you done any of these things or any other work?***		नगद या किसी चीज के रूप में भुगतान दिया जाता है, अन्य महिलायें सामान बेचती हैं, छोटा व्यापार करती हैं, अथवा घर की खेती या घर के व्यापार में हाथ बंटाती हैं. पिछले 12 महीनों में, क्या आपने इनमें से कोई काम या कोई और काम किया है?***
No	Choice	नहीं
Yes	Choice	हाँ
**What kind of work do you mainly do?**		**मुख्यतः आप किस प्रकार का काम करती हों?**
**Home-based earnings** *[stitching, pani-puri making, embroidery, snacks, gota/sitara]*	Choice	**घर पर आधारित काम (आय)** *[सहायता [सिलाई, पानी-पुरी बनाने, कढ़ाई, नाश्ते बनाने, गोटा / सीतारा टकिंग]*
**Vendor job** *[fish selling, vegetable selling, flower/garland selling, snacks selling]*	Choice	**विक्रेता नौकरी*** [मछली बेचने, सब्जी की बिक्री, फूल / माला बेचने, नाश्ते की बिक्री]*

Shop, parlor, saloon owner	Choice	दुकान, पार्लर, सैलून आदि के मालिक
Driver-Taxi/Auto/Cab/Bus	Choice	**ड्राइवर** *[टैक्सी / ऑटो / टैक्सी / बस]*
labour etc.	Choice	घर नौकरानी, सफाई कर्मचारी, निर्माण या कृषि श्रमिक आदि
executive, private job etc.]	Choice	निजी नौकरी आदि]*
Salaried job, consultant, executive, doctor, nurse	Choice	वेतनभोगी नौकरी, परामर्शदाता, अधिकारी, डॉक्टर, नर्स
Other	Choice	अन्य
**Husband/ Partner's information**		**पति/साथी**
**How old is (was) your husband/parner?**	Integer	**आपके पति/साथी की उम्र कितनी है (थी)?**
**What is the highest level of education your husband/partner has (had) completed?**		**आपके पति /साथी की उच्चतम शिक्षा क्या है (थी) ?**
No formal education	Choice	कोई औपचारिक शिक्षा नहीं
Primary (1-5th standard)	Choice	प्राइमरी (1-5th standard)
Middle (6-8th standard)	Choice	मिडिल (6-8th standard)
High school (9-10th standard)	Choice	हाई स्कूल (9-10th standard)
Senior school (11-12th standard)	Choice	सीनियर स्कूल (11-12th standard)
Undergraduate	Choice	ग्रेजुएशन पूरा नहीं
Graduate	Choice	ग्रेजुएशन
Incomplete postgraduate	Choice	पोस्ट ग्रेजुएशन पूरा नहीं
Postgraduate or higher	Choice	पोस्ट ग्रेजुएशन और आगे
Other	Choice	अन्य
Don't know	Choice	नहीं पता
**Is (was) your husband/partner working?**		**क्या आपके पति/ साथी कोई रोजगार या काम करते हैं (थे)?**
No	Choice	नहीं
Yes	Choice	हाँ
Don’t Know	Choice	पता नहीं
**What kind of work does he (did he) mainly do?**		**मुख्यतः आपके पति/साथी किस प्रकार का काम/व्यवसाय करते हैं (थे)?**
**Home-based earnings** *[stitching, pani-puri making, embroidery, snacks, gota/sitara]*	Choice	**घर पर आधारित काम (आय)** [सहायता [सिलाई, पानी-पुरी बनाने, कढ़ाई, नाश्ते बनाने, गोटा / सीतारा टकिंग]
flower/garland selling, snacks selling]	Choice	बिक्री]
etc.]	Choice	**दुकान मालिक** [पार्लर, सैलून, पान, किराना, मांस इत्यादि]

**Driver**-Taxi/Auto/Cab/Bus etc.]	Choice	**ड्राइवर** [टैक्सी / ऑटो / टैक्सी / बस इत्यादि]
labour etc.	Choice	घर नौकरानी, सफाई कर्मचारी, निर्माण या कृषि श्रमिक इत्यादि|
executive, private job etc.]	Choice	निजी नौकरी आदि]
teacher	Choice	वेतनभोगी नौकरी, परामर्शदाता, अधिकारी, डॉक्टर, नर्स, शिक्षक
Don’t Know	Choice	पता नहीं
Other	Choice	अन्य
take drugs?**		**क्या आपके पति/साथी किसी भी तरह के मादक पदार्थ या शराब पीते है (थे)?**
No	Choice	नहीं
Yes	Choice	हाँ
Don’t Know	Choice	पता नहीं
**How often in a month does (did) he take it?**		**आपके पति/साथी एक महीने में कितनी बार नशा करते है (थे)?**
Sometimes	Choice	कभी कभी
Regularly	Choice	नियमित रूप से
Don’t Know	Choice	पता नहीं
had during your life.	Label	जीवनकाल में जन्म दिया हैं.***
**Have you ever been pregnant?**		**क्या आप कभी गर्भवती हुई है?**
No	Choice	नहीं
Yes	Choice	हाँ
**Have you ever given birth to a child?**		**क्या आपने कभी किसी बच्चे को जन्म दिया है?**
No	Choice	नहीं
Yes	Choice	हाँ
**Do you have any (live) children?**		**क्या आपको कोई (जीवित) बच्चे है?**
No	Choice	नहीं
Yes	Choice	हाँ
**Number of living son(s)**	Integer	**जीवित बेटों की संख्या**
**Number of living daughter(s)**	Integer	**जीवित बेटियों की संख्या**
**Household assets**		**हाउसहोल्ड की संपत्ति**
**Which of these things do you have in your home?**		**आपके घर में इनमें से कौन से समान हैं?**
Mattress	Choice	गद्दे

Chair	Choice	कुर्सी
Sofa-set	Choice	सोफा सेट
Cot/bed (Sofa bed)	Choice	बेड (पलंग)/सोफा बेड
Table	Choice	टेबल /मेज
Almirah	Choice	अलमारी
Pressure cooker	Choice	प्रेशर कुकर
Clock/watch	Choice	घड़ी
Metered Electricity	Choice	मीटर बिजली
Radio/transistor	Choice	रेडिओ / ट्रांजिस्टर
Electric fan	Choice	बिजली का पंखा
Cooler	Choice	कूलर
AC	Choice	ए. सी.
Normal TV	Choice	सामान्य टीवी
LED or LCD TV	Choice	एल.इ.डी या एल.सी.डी टी. वी.
Basic mobile phone	Choice	साधारण मोबाइल फ़ोन
Smart phone	Choice	स्मार्ट फ़ोन
Computer (desktop)	Choice	कम्पूटर
Laptop	Choice	लैपटॉप
Induction cooker	Choice	इंडक्शन कुकर
Refrigerator/fridge	Choice	फ्रिज
Mixer-grinder	Choice	मिक्सर
Water purifier	Choice	वाटर प्यूरीफायर
Washing machine	Choice	वॉशिंग मशीन
Sewing machine	Choice	सिलाई मशीन
Water-pump/motor	Choice	पानी का पंप / मोटर
Cycle	Choice	साइकल
Bike/scooter/scooty (two-wheeler)	Choice	बाइक /स्कूटर/स्कूटी/(अन्य दुपहिया)
Car	Choice	कार
Auto or taxi	Choice	रिक्शा/ टैक्सी
Geyser	Choice	ग्रीज़र

Tap drinking water	Choice	नल का पानी
**Do you drink alcohol or take any drug?**		**क्या आप किसी भी तरह के मादक पदार्थ या शराब पीते हैं?**
No	Choice	नहीं
Yes	Choice	हाँ
**How often in a month do you take it?**		**आप इसे महीने में कितनी बार लेते हैं ?**
Sometimes	Choice	कभी कभी
Regularly	Choice	नियमित रूप से
**PHQ9**		**अवसाद (PHQ9)**
bothered by any of the following problems?***		***पिछले 2 हफ्तों (15 दिनों) में, आपको कितनी बार निम्नलिखित समस्याओं से परेशान किया गया है?***
**Little interest or pleasure in doing things**		है.**
Not at all sure	Choice	एक भी दिन नहीं
Several days	Choice	कई दिन
Over half the days	Choice	ज्यादातर दिनों में
Nearly every day	Choice	लगभग हर रोज
**Feeling down, depressed, or hopeless**	As 171	**उदास रहना या हर वक्त निराशा में रहना.**
much**		रहना.**
**Feeling tired or having little energy**		**थकावट सी रहना या कमजोरी महसूस करना.**
**Poor appetite or overeating**		**खाने को दिल नहीं करना या जरूरत से ज्यादा खाना.**
**Feeling bad about yourself or that you are a failure or have let yourself or your family down.**		**खुद के बारे में बुरा महसूस करना या खुद को असफल समझना. या खुद को नीचा समझना क्योकिं आपने परिवार वालों की उम्मीदों पर खरे नहीं उतर पाए.**
**Trouble concentrating on things, such as reading the newspaper or watching television**		**किसी भी काम में मन नहीं लगना जैसे कि कुछ पढना, लिखना या टी.वी. देखना इत्यादि.**
could have noticed. Or the opposite being so figety or restless that you have been moving around a lot more than usual**		**इतने धीरे (अहिस्ता) चलना या इतने धीरे बातें करना कि लोग इस बात को ध्यान (नोटिस) देने लगे या फिर इस का उलट की आप हर समय बहुत जल्दी में और बेचैनी में रहते हों.**
hurting yourself.**		नुकसान पहुचने के बारे में सोचा.**
**GAD7**		**चिंता (GAD7)**
bothered by any of the following problems?***		***पिछले 2 हफ्तों (15 दिनों) में, आपको कितनी बार निम्नलिखित समस्याओं से परेशान किया गया है?***

**Feeling nervous, anxious, or on edge**		**घबराहट या परेशनी महसूस करना.**
Not at all sure	Choice	एक भी दिन नहीं
Several days	Choice	कई दिन
Over half the days	Choice	ज्यादातर दिनों में
Nearly every day	Choice	लगभग हर रोज
**Not being able to stop or control worrying**	As 186	**चिंता को रोक न पाना**
**Worrying too much about different things**	As 186	**हर बात के लिए ज्यादा चिंतित रहना.**
**Trouble relaxing**	As 186	**आराम न कर पाना या मुश्किल होना.**
**Being so restless that it's hard to sit still**	As 186	हों**
**Becoming easily annoyed or irritable**	As 186	**मामूली सी बात पर बेचैन या गुस्सा या नाराज हो जाना.**
**Feeling afraid as if something awful might happen**	As 186	**कोई भयकर घटना घटने का डर या भय महसूस होता हो.**
**Suicidal ideation**		**आत्महत्या करने की प्रवत्ति**
**Apart from in the last two weeks, have you ever thought about ending your life?**		**क्या आपने अपने जीवन में कभी भी आत्महत्या करने के बारे में सोचा है?**
Never	Choice	कभी नहीं
Yes, in the last 6 months	Choice	हाँ, पिछले 6 महीने में
Yes, in the last year	Choice	हाँ, पिछले एक साल में
Yes more than a year ago,	Choice	हाँ, पिछले एक साल से पहले
**Have you ever tried to take your life?**		है?**
**Emotional violence**		**भावनात्मक हिंसा**
which happen to some women. Please tell me if these apply to your relationship with your husband/partner or other family members?*		***अब मैं आपको कुछ परिस्थितियों के बारे में पूछने जा रही हूं जो कुछ महिलाओं के साथ होती हैं. कृपया मुझे बताएं कि क्या ये आपके रिश्ते पर लागू होते हैं?***
you feel bad about yourself?		आपको आपके बारे में बुरा महसूस करवाया?**
Yes	Choice	हाँ
No	Choice	नहीं
**How often has this happened to you?**		**आपके साथ ऐसा कितनी बार हुआ है?**
Once in the last 12 months	Choice	पिछले 12 महीनों मे एक बार
Sometimes in the last 12 months	Choice	पिछले 12 महीनों मे कई बार
Many times in the last 12 months	Choice	पिछले 12 महीनों मे बहुत बार

In the last 15 days	Choice	पिछले 15 दिनों में
None of the above	Choice	इनमें से कोई नहीं
**Who mainly did this to you?**		**आपके साथ यह करने वाला मुख्य व्यक्ति कौन था?**
Husband (Partner)	Choice	पति (साथी)
Natal family members	Choice	मायके (के लोग) वाले
Husband's family members	Choice	ससुराल (के लोग) वाले
Other (stranger, neighbour, teacher, leader, police etc)	Choice	अन्य (अजनबी, पड़ोसी, शिक्षक, नेता, पुलिस आदि)
treated you indifferently?**	As 209	आपके साथ बत्तमीजी से बर्ताव किया?**
something to belittle or humiliate you in front of other people?**	As 209	सामने नीचा दिखाया या आपके साथ ऐसा कुछ व्यवहार किया जिससे लोगो के सामने आपको अपमानित लगे?**
you or intimidate you on purpose (e.g. By the way they looked at you, by yelling and smashing things)? **	As 209	जानबूज कर कुछ किया है (जैसे आपकी तरफ गुस्से से देखा या चिल्लाया या चीजो को तोडा इत्यादि)?
you or someone you care about or take away your children?**	As 209	अपने/ नजदीकी व्यक्ति को चोट या नुकसान पहुचाने या बच्चो को आपसे दूर करने की धमकी दी?**
**Physical violence**		**शारीरिक हिंसा**
happen to some women. Please tell me if anyone has ever done any of the following things to you***		होती हैं. कृपया मुझे बताओ कि क्या आपने कभी भी इनमें से कुछ भी आपके साथ हुआ है.***
**Has anyone in your family ever pushed you, shoved you, shaken you or done something to hurt you?**		**क्या आपके परिवार के किसी भी सदस्य नें कभी भी आपको धक्का दिया, झिंझोडा या आपकी तरफ कोई चीज़ उठाकर फेंकी ताकि आपको चोट पहुचे?**
Yes	Choice	हाँ
No	Choice	नहीं
**How often has this happened to you?**		**आपके साथ ऐसा कितनी बार हुआ है?**
Once in the last 12 months	Choice	पिछले 12 महीनों मे एक बार
Sometimes in the last 12 months	Choice	पिछले 12 महीनों मे कई बार
Many times in the last 12 months	Choice	पिछले 12 महीनों मे बहुत बार
In the last 15 days	Choice	पिछले 15 दिनों में
None of the above	Choice	इनमें से कोई नहीं
**Who mainly did this to you?**		**आपके साथ यह करने वाला मुख्य व्यक्ति कौन था?**
Husband (Partner)	Choice	पति (साथी)
Natal family members	Choice	मायके (के लोग) वाले

Husband's family members	Choice	ससुराल (के लोग) वाले
Other (stranger, neighbour, teacher, leader, police etc)	Choice	अन्य (अजनबी, पड़ोसी, शिक्षक, नेता, पुलिस आदि)
**Has anyone in your family ever twisted your arm, banged your head or pulled your hair?**	As 226	**क्या आपके परिवार के किसी भी सदस्य नें कभी भी आपकी बांह मरोड़ी या आपका सिर पटका या आपके बाल खींचे?**
bitten you?**	As 226	चिमटी काटा या दांतों से काटा?**
**Has anyone in your family ever hit or punched you with their fist or something else that could hurt you?**	As 226	**क्या आपके परिवार के किसी भी सदस्य नें कभी भी, आपको मुक्के मारे या किसी चीज़ से मारा ज़िससे आपको चोट लग सके?**
you or beaten you up?**	As 226	घसीटा या आपको पीटा है?**
or burned you with a cigarette/Bidi, kerosene, chemicals, acid?**	As 226	या आप पर सिगरेट / बीड़ी, मिट्टी का तेल, रसायन, एसिड का उपयोग कर जलाया?**
sharp object such as broken glass, a razor blade, axe, or knife or used any instruments or weapons to threaten or harm you?**	As 226	**क्या आपके परिवार के किसी भी सदस्य नें कभी भी, आप पर किसी तेज़ धार वाले साधन या हथियार जैसे टूटे कांच, रेजर, ब्लेड, कुल्हाड़ी, चाकू इत्यादि से हमला किया या करने की धमकी दी?**
threatened you with a blunt object such as a belt, stone, broomstick, or rolling pin?**	As 226	**क्या आपके परिवार के किसी भी सदस्य नें कभी भी, आपको बेल्ट, पत्थर, झाड़ू या बेलन जैसी या किसी भारी वस्तु से मारा है, या मारने की धमकी दी?**
suffocate, choke, hang you, or poison you on purpose?**	As 226	**क्या आपके परिवार के किसी भी सदस्य नें कभी भी, आपका दम या गला घोंटने या आपको फांसी लगाने या जहर देने की कोशिश की?**
**Sexual violence**		**लैंगिक हिंसा**
happen to some women. Please tell me if anyone has ever done any of the following things to you.***		साथ होती है. आप मुझे बताये की इनमे ने कोई भी बात आप कें साथ किसी ने की है?***
you to have sexual intercourse with him *even when you did not want to*?**		शारीरिक बल के प्रयोग से आपके साथ सोने (सेक्स/ यौन-संबंध) के लिए आपको मजबूर किया?**
Yes	Choice	हाँ
No	Choice	नहीं
**How often has this happened to you?**		**आपके साथ ऐसा कितनी बार हुआ है?**
Once in the last 12 months	Choice	पिछले 12 महीनों मे एक बार
Sometimes in the last 12 months	Choice	पिछले 12 महीनों मे कई बार
Many times in the last 12 months	Choice	पिछले 12 महीनों मे बहुत बार
In the last 15 days	Choice	पिछले 15 दिनों में
None of the above	Choice	इनमें से कोई नहीं

**Who mainly did this to you?**		**आपके साथ यह करने वाला मुख्य व्यक्ति कौन था?**
Husband (Partner)	Choice	पति (साथी)
Natal family members	Choice	मायके (के लोग) वाले
Husband's family members	Choice	ससुराल (के लोग) वाले
Other (stranger, neighbour, teacher, leader, police etc)	Choice	अन्य (अजनबी, पड़ोसी, शिक्षक, नेता, पुलिस आदि)
**Has anyone ever physically forced you to perform any other sexual acts *even you did not want to*?**	As 250	शारीरिक बल से आपको सेक्स/यौन से सम्बंधित कोई भी हरकत (क्रिया) करने को मजबूर किया?**
**other way to perform sexual acts *when you did not want to*?**	As 250	डरा-धमका कर या कोई और तरीके से सेक्स/यौन सम्बन्धी क्रिया (हरकतें) करने के को मजबूर किया?**
**replicate a sexual behaviour from pornography or other sexual material *against your will*?**	As 250	अश्लील साहित्य या अन्य यौन सामग्री दिखाकर आपको वैसे ही यौन व्यवहार (हरकत) को दोहराने के लिए मजबूर किया?**
