# Supplementary material for: Intimate partner violence against women with disability and associated mental health concerns: a cross-sectional survey in Mumbai, India
Source: BMJ Open. 2022 Apr 26;12(4):e056475. doi: 10.1136/bmjopen-2021-056475 (PMC9047698; doi:10.1136/bmjopen-2021-056475)
Supplement: Author's manuscript [file bmjopen-2021-056475.draft_revisions.pdf]

BMJ Open is committed to open peer review. As part of this commitment we make the peer review history of every article we publish publicly available.

When an article is published we post the peer reviewers' comments and the authors' responses online. We also post the versions of the paper that were used during peer review. These are the versions that the peer review comments apply to.

The versions of the paper that follow are the versions that were submitted during the peer review process. They are not the versions of record or the final published versions. They should not be cited or distributed as the published version of this manuscript.

BMJ Open is an open access journal and the full, final, typeset and author-corrected version of record of the manuscript is available on our site with no access controls, subscription charges or pay-per-view fees (http://bmjopen.bmj.com).

If you have any questions on BMJ Open's open peer review process please email info.bmjopen@bmj.com

BMJ Open

Intimate partner violence against women with disability and associated mental health concerns: a cross-sectional survey in Mumbai, India

Journal:	*BMJ Open*
Manuscript ID	bmjopen-2021-056475
Article Type:	Original research
Date Submitted by the Author:	16-Aug-2021

Complete List of Authors:	Riley, Andrew; University of Liverpool, Institute of Population Health<br>Daruwalla, Nayreen; SNEHA<br>Kanougiya, Suman; Tata Institute of Social Sciences (TISS)<br>Gupta, Apoorwa; SNEHA<br>Wickenden, Mary; University of Sussex<br>Osrin, David; University College London

Keywords:	MENTAL HEALTH, PUBLIC HEALTH, SOCIAL MEDICINE

SCHOLARONE™
Manuscripts

I, the Submitting Author has the right to grant and does grant on behalf of all authors of the Work (as defined in the below author licence), an exclusive licence and/or a non-exclusive licence for contributions from authors who are: i) UK Crown employees; ii) where BMJ has agreed a CC-BY licence shall apply, and/or iii) in accordance with the terms applicable for US Federal Government officers or employees acting as part of their official duties; on a worldwide, perpetual, irrevocable, royalty-free basis to BMJ Publishing Group Ltd ("BMJ") its licensees and where the relevant Journal is co-owned by BMJ to the co-owners of the Journal, to publish the Work in this journal and any other BMJ products and to exploit all rights, as set out in our licence.

The Submitting Author accepts and understands that any supply made under these terms is made by BMJ to the Submitting Author unless you are acting as an employee on behalf of your employer or a postgraduate student of an affiliated institution which is paying any applicable article publishing charge ("APC") for Open Access articles. Where the Submitting Author wishes to make the Work available on an Open Access basis (and intends to pay the relevant APC), the terms of reuse of such Open Access shall be governed by a Creative Commons licence – details of these licences and which Creative Commons licence will apply to this Work are set out in our licence referred to above.

Other than as permitted in any relevant BMJ Author's Self Archiving Policies, I confirm this Work has not been accepted for publication elsewhere, is not being considered for publication elsewhere and does not duplicate material already published. I confirm all authors consent to publication of this Work and authorise the granting of this licence.

For peer review only

Intimate partner violence against women with disability and associated mental health concerns: a cross-sectional survey in Mumbai, India

Authors

1. Andrew Riley *Corresponding author*

   Institute of Population Health, University of Liverpool, Liverpool, L69 3BX, UK

   andrew.riley@liverpool.ac.uk

2. Nayreen Daruwalla

   Program on Prevention of Violence Against Women and Children, SNEHA, Mumbai, Maharashtra, 400017, India

   nayreen@snehamumbai.org

3. Suman Kanougiya

   School of Health Systems Studies (SHSS), Tata Institute of Social Sciences (TISS), Mumbai, Maharashtra, India

   suman.kanougiya@gmail.com

4. Apoorwa Deepak Gupta

   Program on Prevention of Violence Against Women and Children, SNEHA, Mumbai, Maharashtra, 400017, India

   apoorwa.gupta@snehamumbai.org

5. Mary Wickenden

   Institute of Development Studies, University of Sussex, Library Rd, Falmer, Brighton BN1 9RE, UK

   M.Wickenden@ids.ac.uk

6. David Osrin

For peer review only

Institute for Global Health, University College London,

London, WC1N IEH, UK

d.osrin@ucl.ac.uk

Abstract

Objectives

The risk of intimate partner violence (IPV) against women with disability is believed to be high. We aimed to compare the prevalence of past-year IPV against women with and without functional difficulties in urban informal settlements, to review its social determinants, and to explore its association with mental health.

Design

Cross-sectional survey

Setting

Fifty clusters within four informal settlements.

Participants

For peer review only

5122 women aged 18 to 49 years.

Primary and secondary outcome measures

We used the Washington Group Short Set of Questions to assess functional difficulties. IPV in the past year was described by binary composites of questions about physical, sexual, and emotional violence. We screened for symptoms of depression using the Patient Health Questionnaire 9 and of anxiety using the Generalized Anxiety Disorder 7. Multivariable logistic regression models examined associations between functional difficulties, IPV, and mental health.

Results

The 10% of respondents who screened positive for functional disability had greater odds of experiencing physical or sexual IPV (Adjusted Odds Ratio (AOR) 1.68, 95% CI 1.23, 2.29) and emotional IPV (1.52, CI 1.16, 2.00) than women who screened negative. Women who screened positive for functional disability had greater odds than women who screened negative of symptoms suggesting moderate or severe anxiety (AOR 2.50, CI 1.78, 3.49), depression (2.91, CI 2.13, 3.99), and suicidal thinking (AOR 1.94, CI 1.50, 2.50).

Conclusions

The burden of IPV fell disproportionately on women with functional difficulties, who were also more likely to screen positive for common mental disorder. Public health initiatives need to respond at local and national levels to address the overlapping and mutually reinforcing determinants of violence, while existing policy needs to be better utilised to ensure protection for the most vulnerable.

For peer review only

Keywords

Intimate partner violence; Domestic violence; Disability; India; Poverty areas

Strengths and limitations of this study

- Data came from a large cross-sectional survey of women in informal settlements.
- Data were collected by an organisation providing support for survivors of intimate partner violence.
- The study addresses a neglected area of research: the experience of disabled women in India.
- Functional difficulties were self-reported and not the primary focus of the survey.
- The cross-sectional nature of the study limits the possibility of causal inference.

For peer review only

Background

Approximately 15% of the world's population, around one billion people, live with a physical, intellectual, sensory, or mental health impairment that affects their daily lives and long-term health and wellbeing [1]. Disability is the product of attitudinal and environmental barriers that limit persons with functional difficulties from fully participating in society [2]. These barriers mean that, compared with non-disabled people, people with disability are less likely to be educated [3], financially secure [4], employed [5], have access to health services [1], have their health needs met [1, 6], and have their social needs met [7-9].

The double burden on women and girls with disability means that they are among the more marginalised and vulnerable in society, particularly when this burden intersects with multidimensional poverty [1]. Lacking physical and financial independence or access to socially inclusive services, disabled women who depend on partners and families for support are vulnerable to further social marginalisation and isolation and are at higher risk of physical violence and sexual abuse [10, 11], as well as other forms of abuse such as neglect, exploitation, and coercive control [12]. Irrespective of

For peer review only - http://bmjopen.bmj.com/site/about/guidelines.xhtml

disability, violence against women occurs most commonly in the home, in the form of intimate partner violence (IPV) or non-partner domestic violence [13, 14]. Women with disability are believed to be at greater risk of these and other forms of violence. However, a dearth of population-level research [15], and the typically hidden nature of violence [16], mean that little is known of how disability status may lead to additional risks of IPV. Such research is important for informing IPV prevention programmes [17, 18], and for meeting the objectives of the United Nations Sustainable Development Goals.

In India, the 2011 national census [19] and the 2018 National Sample Survey (NSS) [20] both estimated disability prevalence at 2.2% (~26.8 million people), although these are considered underestimates [21, 22]. Other national estimates range from 8% by the World Bank [22], to 25% by the World Health Survey [1, 23]. More local estimates reflect different approaches to classification and include 1.6% in Tamil Nadu State [24], 1.9% in a Delhi slum community [25] and 12.2% in Telangana State [26]. Disability is most common among older, rural women and members of scheduled tribes and

For peer review only - http://bmjopen.bmj.com/site/about/guidelines.xhtml
For peer review only

scheduled castes [20, 27], while prevalence increases with age, among lower-socioeconomic groups and those without employment / the unemployed [24, 26]. Disabled women often report lower rates of marriage [20, 26], and higher rates of widowhood, separation, and divorce [20, 24].

The ubiquity of violence against women in India has been well reported in both popular media and population-level research. The fourth National Family Health Survey (NFHS-4) reported that 30% of married women had experienced physical, sexual, or emotional violence in their lifetime and 26% in the past year [28]. Similar rates were reported in a nationally representative systematic review [29] and in urban and slum settings [30-34]. Violence against women with disabilities in India is less documented: to date, only three major studies to date have reported on it at population level. A survey in Mumbai involving women aged 15-49 years with functional impairments (visual, locomotor, hearing) found that 20% of 123 ever-married disabled women had experienced past-year physical IPV, 23% emotional IPV (being insulted, humiliated or threatened), and 10% sexual IPV. Violence perpetrated by people other than intimate

For peer review only - http://bmjopen.bmj.com/site/about/guidelines.xhtml

partners was experienced by 18% of ever-married and 23% of unmarried women, most commonly by mothers-in-law in the marital family and brothers in the natal [11].

In Karnataka, of 70 female interviewees with disability, 73% reported ‘significant’ violence (versus ‘rare’ or ‘occasional’) and 23% sexual violence in the previous 12 months, while 59% reported generalised violence and 10% sexual violence in their childhood. Around half of this violence was perpetrated by family members (46%) or by people outside the family (54%) [35]. In a study involving 729 women in Odisha State, 48% of those with learning difficulties and 23% with physical impairments reported experiencing domestic violence [36]. While these studies were limited by small samples and the latter two by use of convenience sampling [35, 36], similar findings have been reported in the wider South Asia region [37-39].

For all women, violence increases the risk of serious injury and mortality [40] [41], HIV and sexually transmitted infections [42, 43], unintended pregnancy [44], reproductive health morbidity [45, 46] and a range of mental health concerns [47-49]. Among women with disabilities in India who have experienced violence, high levels of severe mental

For peer review only - http://bmjopen.bmj.com/site/about/guidelines.xhtml

distress, depression, suicidal ideation and attempted suicide have been documented [11].

Given its impact on physical and mental health, as well as being a matter of individual and social justice, violence against disabled women is largely absent from the literature. This absence limits the capacity of health planners and policymakers to act, while also contributing to the ongoing marginalisation of disabled communities, experiences, and concerns.

Objectives

This study sought to address the gap in understanding of the sociodemographic circumstances of women with disabilities in urban India and their experience of intimate partner violence. We aimed to identify associations between individual socioeconomic determinants and disability in women aged 18 to 49 years living in informal settlements in Mumbai and to test the hypothesis that women with functional difficulty/disability are more likely to have experienced recent intimate partner violence and have higher rates of common mental disorders.

For peer review only - http://bmjopen.bmj.com/site/about/guidelines.xhtml

Methods

Setting

Maharashtra ranks fourth among Indian states in the human development index [50], and women tend to have higher than the national averages on indicators of literacy, marriage age and workforce participation. Mumbai, Maharashtra's capital, has approximately 12.7 million residents, 41% of whom live in informal settlements [51, 52]. These are characterised by overcrowding, lack of tenure and insufficient living space, housing durability, and water and sanitation facilities [53, 54]. Women in these communities typically depend on relatives for housing, making them vulnerable to homelessness, poverty and violence [54].

Design

We used data collected by SNEHA (Society for Nutrition, Education and Health Action), a non-government organization working in Mumbai's disadvantaged communities to improve the health of women and children and address the burden of violence. The dataset came from a survey conducted before the implementation of a community-based intervention to prevent violence against women and girls through

For peer review only - http://bmjopen.bmj.com/site/about/guidelines.xhtml

individual volunteers and groups [55]. The survey included 50 clusters of equal size, each of ~500 dwellings, covering a population of ~77,000 in four areas within major informal settlements. The participants were women aged 18-49 years who consented to interview.

Data collection

Data collection has been described in detail elsewhere [56]. Clusters were mapped and homes visited to identify potential participants. One respondent per household was sampled (~100 respondents per cluster). Data collectors visited participants to explain the survey, discuss the process, answer questions, and provide a participant information sheet. Signed consent to interview was obtained and participants were assured of data confidentiality. Participants were asked about demographic characteristics, socioeconomic indicators, their health, wellbeing, functional difficulty and disability status, common mental disorders, their experience of emotional, physical, and sexual domestic violence, injuries, help-seeking, support and disclosure, and spousal drug and alcohol use. All participant information was confidential and no names or identifiable details appear in outputs or analyses. Sources of questions included the National Family Health Survey (NFHS-4) [28], the Indian Family Violence

For peer review only - http://bmjopen.bmj.com/site/about/guidelines.xhtml

and Control Scale [57], the International Violence Against Women Survey [58] and the WHO multi-country study [59]. Questions were translated where necessary, back-translated, piloted, and refined.

Sample size

An estimate of prevalence from a cross-sectional sample of 5000 in a population of 125,000 would have a precision of ~1%. Within this, a comparison of two categories of determinant for 100 respondents in each of 50 clusters would provide 80% power at 5% significance level to detect a difference of 6% in prevalence estimates of 10-20%.

Variables

Disability was assessed using the Washington Group Short Set of Questions (WG-SS), based on the International Classification of Functioning, Disability and Health (ICF) model of disability [60]. The questions cover six functional domains (*seeing, hearing, walking, memory, self-care, speaking),* with four categories of response (*no difficulty, some difficulty, a lot of difficulty, cannot do at all*) [60]. For this analysis, a respondent was classified as having disability if they reported 'some difficulty' in at

For peer review only - http://bmjopen.bmj.com/site/about/guidelines.xhtml

least one domain. This cut-off is one of several possible approaches and has been used in a similar setting [37].

Definitions of violence follow the WHO Multi-Country Study [59] and are discussed in detail elsewhere [56]. We screened for anxiety, depression, and suicidal thinking as common mental disorders. Anxiety was screened for using the Generalized Anxiety Disorder 7-item (GAD-7) scale [61]. Depression was screened for using the Patient Health Questionnaire (PHQ-9) [62]. For both instruments, a cut-off score of ≥10 has been used as suggestive of clinically relevant conditions requiring further assessment [63]. In this study, variables were coded 'yes' for depression and anxiety if responses were recorded as moderate or severe. Suicidal thinking was coded 'yes' for lifetime positive responses.

Covariates corresponded to determinants of risk for IPV identified predominantly from the wider body of Indian IPV research undertaken in comparable settings with women whose disability status was not identified. They were categorised following Heise's ecologic framework for violence against women [64, 65]. In this framework, risk of IPV

For peer review only - http://bmjopen.bmj.com/site/about/guidelines.xhtml

is best understood as a function of multiple mutually interacting factors operating within and across different levels: woman, relationship, household, community, and society. For example, the risk of IPV may be greater in households in which a wife is working and a husband unemployed in a social environment that privileges traditional male roles.

We hypothesised that a woman's odds of experiencing IPV would increase with age [66] [67] [68], number of children [66] [40], and employment outside the home [69] [70] [71], and would decrease with years of education [32] [72] [40]. Partner use of alcohol or drugs was hypothesised to increase the odds [30] [73] [74] [67] [75], while no clear relationship between IPV and partner level of education was predicted [33] [76] [66] [77]. Lower socioeconomic status was considered as potentially increasing the odds of IPV [11] [33] [34] [40] [74] [78], as were faith [67] [78] and caste [79] [67], due to the association of some groups with lower socioeconomic status.

Associations between common mental disorders and sociodemographic characteristics were hypothesised to conform to a similar pattern due to their close

For peer review only - http://bmjopen.bmj.com/site/about/guidelines.xhtml

association with IPV. Hypothetical determinants were modelled as independent variables in both univariable and multivariable analyses [80]. At ‘woman’ level, age-group, marital status, parity, education and employment were entered as categorical variables. Categories of age and schooling were based on those used in other studies for comparability. Individual employment categories were recoded as either home-based or outside the home as IPV has been associated with women working outside the home in general rather than individual employment categories [69] [70] [71]. At ‘partner’ level, husband’s schooling and employment were entered as categorical indicators and alcohol or drug use as binary. At ‘household’ level, faith, caste and socioeconomic quintile were entered as categorical indicators. Socioeconomic quintile was entered as a categorical rather than continuous variable to retain categorical consistency and because it was hypothesised that a reduction in odds would be observed in the wealthier quintiles. Quintiles were based on analysis of 22 individual household assets, with scores derived from standardized weights from the first component of a principal components analysis [81] [82].

Statistical analysis

For peer review only - http://bmjopen.bmj.com/site/about/guidelines.xhtml

The survey had a single-stage design with clustering and stratification. Data analyses were unweighted because the clusters were of similar size and each was sampled for ~100 questionnaires. Sociodemographic factors were summarised and cross-tabulated using frequencies and percentages, and univariable logistic regression models used to test associations between each exposure variable and the outcome. Variables with associations at $p<0.1$ were included in multivariable analysis. Multivariable logistic regression was used to examine associations between outcome variables (disability, IPV, depression, anxiety) and primary exposure variables, adjusting for confounders (individual demographic and socioeconomic characteristics). Post hoc Wald tests were performed to check models. All analyses used survey commands in Stata 15.1 (College Station, TX).

Ethical considerations

The survey involved disclosing personal and sensitive information, which raises issues of interviewer behaviour, consent, data sharing, and privacy and confidentiality. All interviewers underwent appropriate training and WHO ethical and safety recommendations were followed [83]. Interview processes were checked through random visits and systematic monitoring by project officers and supervisors. During

For peer review only - http://bmjopen.bmj.com/site/about/guidelines.xhtml

the interview, participants were made aware of the potentially sensitive nature of the questions about violence and consent was obtained before they began. Efforts were made to interview participants alone, mostly in their homes. If a researcher was concerned about privacy, an arrangement was made for a second visit. If household members were unwilling to leave, researchers encouraged them to listen to some of the routine questions to be reassured of the survey's nature: most were amenable to leaving. All participants were given information on local counselling and crisis support services for themselves or other women, whether they reported violence or not. Service providers were made aware of the study and the potential for increased service use. Researchers were able to arrange referrals if requested. Data collection was approved by the UCL Research Ethics Committee (3546/003 27/09/2017) and the PUKAR (Partners for Urban Knowledge, Action and Research) Institutional Ethics Committee (25/12/2017) [55].

Patient and public involvement

Our research responds to the urgency of preventing violence and improving services for survivors, primarily through community-based programming in informal settlements. It is driven by survivors' needs, one of which is inclusion. We followed a

For peer review only

protocol co-developed with community members to achieve maximal inclusion of women with functional difficulties. We discussed the survey questions with participants in a pilot phase and adapted them for subsequent use. Our findings will be shared through community women's groups, three of which meet monthly in each of the 50 informal settlement clusters.

For peer review only

Results

Interviewers approached 5277 households between 5th December 2017 and 28th March 2019, of which 967 (5%) had no eligible woman resident. They could not achieve privacy in 592 homes (11%) and 155 (3%) potential respondents declined interview. They conducted 5122 interviews.

Disability prevalence

Table 1 summarises the type and frequency of functional difficulties reported by women. 10% had 'some difficulty' in at least one domain, of which the most common were visual (4%) and locomotor (5%). Across all domains, the number of respondents reporting a lot of difficulty or no ability at all was low, each less than 1%.

Table 1: Frequencies of domains of functional difficulty among 5122 women aged 18-49 years living in informal settlements in Mumbai

Functional difficulty	None n (%)	Some difficulty n (%)	A lot of difficulty n (%)	Cannot do at all n (%)
Seeing	4896 (96)	212 (4)	12 (<1)	2 (<1)
Hearing	5078 (99)	40 (<1)	3 (<1)	1 (<1)
Walking	4860 (95)	234 (5)	25 (<1)	3 (<1)
Memory	4988 (97)	123 (2)	11 (<1)	0 (0)
Self-care	5108 (>99)	10 (<1)	4 (<1)	0 (0)
Speaking	5109 (>99)	4 (<1)	9 (<1)	0 (0)

Washington Group Disability prevalence assessment	n (%)
At least one domain *some difficulty*	505 (10)
At least one domain *a lot of difficulty*	63 (1)
At least one domain *cannot at all*	6 (<1)
At least one domain *a lot of difficulty* or *cannot at all* or at least *some difficulty* in two domains	178 (3)

Women's sociodemographic profiles and disability

Most women were in their 20s or 30s (80%), currently married (92%) and with one or two children (51%). A fifth of women (19%) reported having no schooling, while 44% had had six to 10 years. Having no remunerated work was common (76%), and those

For peer review only - http://bmjopen.bmj.com/site/about/guidelines.xhtml
For peer review only

who earned tended to work from home (15%). In unadjusted analyses, greater odds of disability were observed in women over 40 (odds ratio 2.7; 95% confidence interval 1.3, 5.5), women who were separated or divorced (3.3; 1.9, 5.6), or widowed (3.6; 2.2, 5.9), and women who had three or more children (1.9; 1.4, 2.4). Attending school for at least six years was associated with lesser odds of disability than no schooling at all (0.8; 0.6, 0.9). Women who earned in the home had greater odds of disability than women who did not (1.5; 1.1, 2.0), as did women from Muslim rather than Hindu families (1.4; 1.1, 1.8).

Associations between disability and past-year IPV

Table 2 presents associations between women's functional difficulty and past-year physical, sexual, and emotional IPV. Of 5122 women, 628 reported suffering physical or sexual IPV in the last year (12%) and 606 had suffered emotional IPV (12%). These proportions were greater for women with disability: 84 of 505 who reported at least some functional difficulty had suffered physical or sexual IPV (17%) and 83 emotional IPV (16%). We had information on socioeconomic asset score for 4918 women who had ever been married, and on their husbands for 4805 women whose husbands were alive. After adjustment for age, marital status, parity, woman's and husband's

For peer review only

schooling, woman's and husband's employment, religion, and husband's use of alcohol or drugs, these differences were manifest in adjusted odds ratios of 1.68 (95% CI 1.23, 2.29) for physical or sexual and 1.52 (1.16, 2.00) for emotional IPV.

For peer review only

Table 2: Associations between experience of intimate partner violence (physical or sexual IPV, emotional IPV) in the preceding year, disability, and sociodemographic characteristics, for 5122 women aged 18-49 years living in informal settlements in Mumbai

Characteristic (n)	Physical or sexual IPV in last year							Emotional IPV in last year						
	No n (%)	Yes n (%)	OR	(95% CI)	p-value	Adjusted OR	(95% CI)	No n (%)	Yes n (%)	OR	(95% CI)	p-value	Adjusted OR	(95% CI)
Disability														
None	4073 (88)	544 (12)	1			1		4093 (89)	524 (11)	1			1	
At least some difficulty	421 (83)	84 (17)	1.49	1.24, 1.99	0.007	1.68	1.23, 2.29	422 (84)	83 (16)	1.54	1.20, 1.97	0.001	1.52	1.16, 2.00
All	4494 (88)	628 (12)						4515 (88)	607 (12)					
Woman's age (years)														
<20	104 (90)	11 (10)	1			1		108 (94)	7 (6)	1			-	-
20-29	1747 (86)	291 (14)	1.57	0.54, 4.64		0.82	0.26, 2.56	1782 (87)	256 (13)	2.22	0.80, 6.16		-	-
30-39	1808 (88)	248 (12)	1.30	0.43, 3.88		0.57	0.18, 1.86	1812 (88)	243 (12)	2.07	0.74, 5.81		-	-
>40	835 (91)	78 (9)	0.88	0.28, 2.75	<0.001	0.39	0.11, 1.36	812 (89)	101 (11)	1.92	0.69, 5.34	0.329	-	-
All	4494 (88)	628 (12)						4515 (88)	607 (12)					
Marital status														
Married	4112 (87)	593 (13)	1			1		4131 (88)	574 (12)	1			1	
Unmarried	203 (99)	2 (1)	0.07	0.02, 0.27		0.07	0.02, 0.23	204 (99)	1 (<1)	0.04	0.01, 0.26		0.03	0.01, 0.24
Separated or divorced	78 (78)	22 (22)	1.96	1.20, 3.18		1.44	0.88, 2.33	71 (71)	29 (29)	2.94	1.90, 4.54		2.14	1.37, 3.35
Widowed	101 (90)	11 (10)	0.76	0.44, 1.29	0.001	0.79	0.45, 1.41	109 (97)	3 (3)	0.20	0.07, 0.59	<0.001	0.14	0.04, 0.44
All	4494 (88)	628 (12)						4515 (88)	607 (12)					
Parity														
No children	692 (92)	63 (8)	1			1		692 (92)	63 (8)	1			1	
1 or 2	2263 (86)	358 (14)	1.74	1.29, 2.34		1.41	1.04, 1.91	2279 (87)	342 (13)	1.65	1.28, 2.13		1.16	0.89, 1.51
3 or more	1539 (88)	207 (12)	1.48	1.09, 2.00	0.002	1.29	0.92, 1.80	1544 (88)	202 (12)	1.44	1.09, 1.90	0.002	0.96	0.70, 1.31
All	4494 (88)	628 (12)						4515 (88)	607 (12)					

For peer review only

For peer review only - http://bmjopen.bmj.com/site/about/guidelines.xhtml

Woman's schooling														
None	848 (89)	100 (11)	1			1		854 (90)	94 (10)	1			1	
1-5 years	734 (85)	125 (15)	1.44	1.03, 2.02		1.39	0.99, 1.96	737 (86)	122 (14)	1.50	1.13, 2.00		1.45	1.09, 1.94
6-10 years	1976 (87)	304 (13)	1.30	1.00, 1.70		1.24	0.93, 1.66	1983 (87)	297 (13)	1.36	1.07, 1.73		1.29	1.00, 1.67
11 years or more	936 (90)	99 (10)	0.90	0.66, 1.21	0.027	0.93	0.67, 1.29	941 (91)	94 (9)	0.91	0.72, 1.15	0.002	0.95	0.72, 1.24
All	4494 (88)	628 (12)						4515 (88)	607 (12)					
Woman's employment														
None	3437 (89)	433 (11)	1			1		3446 (89)	424 (11)	1			1	
Home-based	665 (86)	108 (14)	1.29	1.07, 1.56		1.22	1.01, 1.49	677 (88)	96 (12)	1.15	0.92, 1.44		1.08	0.85, 1.37
Outside the home	392 (82)	87 (18)	1.76	1.33, 2.32	<0.001	2.11	1.60, 2.80	392 (82)	87 (18)	1.80	1.31, 2.49	0.003	2.00	1.43, 2.80
All	4494 (88)	628 (12)						4515 (88)	607 (12)					

Table 2 continued

Religion														
Hindu	2685 (89)	317 (11)	1			1		2677 (89)	325 (11)	1			1	
Muslim	1629 (86)	273 (14)	1.42	1.15, 1.76		1.38	1.11, 1.72	1655 (87)	247 (13)	1.23	1.00, 1.52		1.23	1.00, 1.54
Buddhist	163 (83)	34 (17)	1.77	1.25, 2.50		1.68	1.16, 2.43	167 (85)	30 (15)	1.48	1.11, 1.97		1.33	1.00, 1.79
Other	17 (81)	4 (19)	1.99	0.64, 6.23	0.003	1.90	0.63, 5.74	16 (76)	5 (24)	2.57	0.90, 7.36	0.013	2.94	0.93, 9.27
All	4494 (88)	628 (12)						4515 (88)	607 (12)					
Caste														
General	2637 (88)	350 (12)	1			-	-	2651 (89)	336 (11)	1			-	-
Other Backward Caste	1064 (87)	157 (13)	1.11	0.92, 1.35		-	-	1063 (87)	158 (13)	1.17	0.95, 1.45		-	-
Scheduled Caste/Tribe	621 (86)	103 (14)	1.25	1.01, 1.54		-	-	629 (87)	95 (13)	1.19	0.93, 1.53		-	-
None stated	172 (91)	18 (9)	0.79	0.45, 1.37	0.202	-	-	172 (91)	18 (9)	0.83	0.47, 1.45	0.375	-	-
All	4494 (88)	628 (12)						4515 (88)	607 (12)					
Socioeconomic quintile (n=4918 ever-married women)														
1 poorest	829 (84)	161 (16)	1			1		849 (86)	141 (14)	1			-	-
2	861 (88)	117 (12)	0.70	0.55, 0.88		0.70	0.54, 0.90	865 (88)	113 (12)	0.79	0.58, 1.07		-	-

For peer review only - http://bmjopen.bmj.com/site/about/guidelines.xhtml

3	865 (88)	118 (12)	0.70	0.52, 0.95		0.72	0.53, 0.99	871 (89)	112 (11)	0.77	0.53, 1.13		-	-
4	876 (89)	108 (11)	0.63	0.49, 0.83		0.66	0.50, 0.88	876 (89)	108 (11)	0.74	0.53, 1.04		-	-
5 least poor	884 (90)	99 (10)	0.58	0.41, 0.81	0.007	0.63	0.45, 0.89	875 (89)	108 (11)	0.74	0.53, 1.04	0.325	-	-
All	4315 (88)	603 (12)						4336 (88)	582 (12)					
Husband's schooling (n=4805 women with living husbands)														
None	391 (81)	90 (19)	1			1		396 (82)	85 (18)	1			1	
1-5 years	507 (86)	83 (14)	0.71	0.51, 1.00		0.73	0.51, 1.04	509 (86)	81 (14)	0.74	0.50, 1.10		0.76	0.51, 1.11
6-10 years	2206 (87)	318 (13)	0.63	0.48, 0.82		0.66	0.50, 0.87	2214 (88)	310 (12)	0.65	0.48, 0.89		0.68	0.50, 0.92
11 years or more	1069 (89)	123 (11)	0.54	0.39, 0.75		0.63	0.41, 0.97	1066 (89)	126 (11)	0.59	0.43, 0.83		0.68	0.49, 0.95
Unknown	17 (91)	1 (9)	0.44	0.29, 0.66	<0.001	0.55	0.37, 0.84	17 (91)	1 (9)	0.48	0.33, 0.70	0.005	0.60	0.40, 0.92
All	4190 (87)	615 (13)						4202 (87)	603 (13)					
Husband employed in previous 12 months (n=4805 women with living husbands)														
No	73 (74)	26 (26)	1			1		76 (77)	23 (23)	1			1	
Yes	4111 (88)	586 (12)	0.40	0.25, 0.64		0.50	0.29, 0.84	4120 (88)	577 (12)	0.46	0.28, 0.76		0.75	0.45, 1.27
Unknown	6 (67)	3 (33)	1.40	0.35, 5.60	0.001	1.20	0.21, 6.91	6 (67)	3 (33)	1.65	0.32, 8.42	0.001	1.34	0.34, 5.27
All	4190 (87)	615 (13)						4202 (87)	603 (13)					
Husband uses alcohol/drugs (n=4805 women with living husbands)														
No	3198 (89)	377 (11)	1			1		3216 (90)	359 (10)	1			1	
Yes	992 (81)	238 (19)	2.04	1.59, 2.61	<0.001	1.81	1.36, 2.40	986 (80)	244 (20)	2.22	1.79, 2.75	<0.001	2.00	1.59, 2.53
All	4190 (87)	615 (13)						4202 (87)	603 (13)					

IPV: intimate partner violence. OR: odds ratio. AOR: adjusted odds ratio. Ref: reference category. ORs and 95% confidence intervals (CI) from univariable and multivariable logistic regression models. Multivariable models (Adjusted OR) include all variables for which Adjusted ORs are presented. Of 5122 women, we had data on socioeconomic status for the 4918 who had ever been married and data on their husbands for 4805 who had not been widowed.

For peer review only - http://bmjopen.bmj.com/site/about/guidelines.xhtml

Associations between disability and symptoms of anxiety and depression

Table 3 compares the profiles of women screened as having symptoms of at least moderate anxiety or depression with those who did not. 10% of women fell into this category and disabled women had nearly three times greater odds of doing so than non-disabled women (AOR 2.88, 95% CI 2.17, 3.82). Women who reported physical or sexual IPV (AOR 2.50, CI 1.77, 3.53) or emotional IPV (AOR 2.34, CI 1.59, 3.43) also had greater odds of anxiety or depression. Associations with anxiety or depression were also seen for separated, divorced, or widowed women, who were engaged in remunerated work, and whose husbands used alcohol or drugs. Associations were not seen in unadjusted models for parity or caste.

Table 3: Associations of positive screens for moderate or severe anxiety (GAD-7) or depression (PHQ-9) with disability and intimate partner violence (IPV) in the preceding year, for 5122 women aged 18-49 years living in informal settlements in Mumbai

Characteristic (n)	No, minimal, or mild anxiety or depression n (%)	Moderate or severe anxiety or depression n (%)	OR	(95% CI)	p	Adjusted OR	(95% CI)
Disability							
None	4217 (91)	400 (9)	1			1	
At least some impairment	372 (74)	133 (26)	3.77	2.99, 4.75	<0.001	2.88	2.17, 3.82
All	4589 (90)	533 (10)					
Physical or sexual IPV							

For peer review only

No	4132 (92)	362 (8)	1			1	
Yes	457 (73)	171 (27)	4.27	3.35, 5.45	<0.001	2.50	1.77, 3.53
All	4589 (90)	533 (10)					
Emotional IPV							
No	4150 (92)	365 (8)	1			1	
Yes	439 (72)	168 (28)	4.35	3.37, 5.62	<0.001	2.34	1.59, 3.43
All	4589 (90)	533 (10)					
Woman's age (years)							
<20	102 (89)	13 (11)	1			1	
20-29	1854 (91)	184 (9)	0.78	0.44, 1.38		0.69	0.38, 1.24
30-39	1857 (90)	199 (10)	0.84	0.47, 1.51		0.70	0.38, 1.28
>40	776 (85)	137 (15)	1.39	0.75, 2.56	<0.001	0.95	0.50, 1.82
All	4589 (90)	533 (10)					
Marital status							
Married	4267 (91)	438 (9)	1			1	
Unmarried	189 (92)	16 (8)	0.82	0.46, 1.48		1.21	0.64, 2.25
Separated or divorced	57 (57)	43 (43)	7.35	4.72, 11.45		4.73	2.68, 9.34
Widowed	76 (68)	36 (32)	4.61	2.91, 7.33	<0.001	3.25	1.72, 6.12
All	4589 (90)	533 (10)					
Woman's schooling							
None	819 (86)	129 (14)	1 =			1	
1-5 years	758 (88)	101 (12)	0.85	0.61, 1.17		0.72	0.50, 1.06
6-10 years	2046 (90)	234 (10)	0.73	0.56, 0.94		0.70	0.50, 0.96
11 years or more	966 (93)	69 (7)	0.45	0.32, 0.65	<0.001	0.59	0.39, 0.90
All	4589 (90)	533 (10)					
Woman's employment							
None	3535 (91)	335 (9)	1			1	
Home-based	662 (86)	111 (14)	1.77	1.39, 2.25		1.48	1.12, 1.96
Outside the home	392 (82)	87 (18)	2.34	1.73, 3.18	<0.001	1.70	1.19, 2.44
All	4589 (90)	533 (10)					
Religion							
Hindu	2737 (91)	265 (9)	1			1	
Muslim	1669 (88)	233 (12)	1.44	1.15, 1.81		1.28	0.98, 1.67
Buddhist	165 (84)	32 (16)	2.00	1.27, 3.17		1.63	0.98, 2.70
Other	18 (86)	3 (14)	1.72	0.45, 6.61	0.003	1.13	0.40, 3.22
All	4589 (90)	533 (10)					
Socioeconomic quintile (n=4918 ever-married women)							
1 poorest	857 (87)	133 (13)	1			1	
2	877 (90)	101 (10)	0.74	0.56, 0.98		0.73	0.53, 1.02
3	879 (89)	104 (11)	0.76	0.56, 1.04		0.77	0.54, 1.11
4	891 (91)	93 (9)	0.67	0.50, 0.90		0.68	0.48, 0.96
5 least poor	903 (92)	80 (8)	0.57	0.41, 0.80	0.014	0.64	0.43, 0.96
All	4407 (90)	511 (10)					
Husband's schooling (n=4805 women with living husbands)							

For peer review only - http://bmjopen.bmj.com/site/about/guidelines.xhtml

None	410 (85)	71 (15)	1			1	
1-5 years	517 (88)	73 (12)	0.82	0.59, 1.13		1.04	0.76, 1.44
6-10 years	2282 (90)	242 (10)	0.61	0.44, 0.84		1.01	0.73, 1.38
11 years or more	1103 (91)	89 (9)	0.58	0.38, 0.91		1.19	0.72, 1.95
Unknown	12 (94)	6 (6)	0.37	0.24, 0.58	<0.001	0.84	0.47, 1.50
All	4324 (90)	481 (10)					
Husband employed in previous 12 months (n=4805 women with living husbands)							
No	72 (73)	27 (27)	1			1	
Yes	4247 (90)	450 (10)	0.28	0.19, 0.42		0.81	0.45, 1.48
Unknown	5 (56)	4 (44)	2.13	0.49, 9.23	<0.001	1.70	0.29, 9.94
All	4324 (90)	481 (10)					
Husband uses alcohol or drugs (n=4805 women with living husbands)							
No	3317 (93)	258 (7)	1			1	
Yes	1007 (82)	223 (18)	2.85	2.19, 3.70	<0.001	1.98	1.48, 2.64
All	4324 (90)	481 (10)					

OR: odds ratio. AOR: adjusted odds ratio. ORs and 95% confidence intervals (CI) from univariable and multivariable logistic regression models.

Multivariable models (Adjusted OR) include all variables for which Adjusted ORs are presented. Of 5122 women, we had data on socioeconomic status for the 4918 who had ever been married, and data on their husbands for 4805 who had not been widowed.

Table 4 examines associations in more detail, presenting odds ratios for positive screens for moderate or severe anxiety, depression, and suicidal thinking in the presence of disability and physical, sexual, or emotional IPV. Overall, 6% of women screened positive for anxiety symptoms and 9% for symptoms of depression, while 13% had experienced suicidal thinking. In unadjusted models, disability increased the odds of each of these by two to four times. Adjusting for potential confounders reduced the odds ratios, but they remained substantial. IPV may also be a confounding factor, as it is associated with both mental health concerns and disability. Adjusting for IPV

For peer review only

and sociodemographic characteristics further reduced the odds ratios, but women with disability continued to have elevated odds of having a mental health concern. In this second adjusted model, women with functional difficulty had more than twice the odds of reporting anxiety symptoms as non-disabled women (AOR2 2.50, CI 1.78, 3.49) and nearly three times the odds of depression (AOR2 2.91, CI 2.13, 3.99). They had almost twice the odds of reporting suicidal thinking (AOR2 1.94, CI 1.50, 2.50). Independent of functional status, physical, sexual and emotional IPV were all associated with greater odds of having a mental health concern.

Table 4: Unadjusted and adjusted logistic regression models for associations between disability status, symptoms of anxiety and depression, and suicidal thinking, for 5122 women aged 18-49 years living in informal settlements in Mumbai

	Moderate or severe anxiety n (%)		OR	(95% CI)	Adjusted OR 1	(95% CI)	Adjusted OR 2	(95% CI)
	No	Yes						
Disability status								
None	4389 (95)	228 (5)	1		1		1	
At least some difficulty	425 (84)	80 (16)	3.62	2.77, 4.73	2.75	2.01, 3.76	2.50	1.78, 3.49
All	4814 (94)	308 (6)						
Physical or sexual IPV								
No	4293 (95)	201 (4)	1		1			
Yes	521 (83)	107 (17)	4.39	3.41, 5.65	4.05	2.98, 5.50	-	-
All	4814 (94)	308 (6)						
Emotional IPV								
No	4315 (96)	200 (4)	1		1			
Yes	499 (82)	108 (18)	4.67	3.59, 6.07	4.11	3.08, 5.49	-	-
All	4814 (94)	308 (6)						

For peer review only - http://bmjopen.bmj.com/site/about/guidelines.xhtml

	Moderate or severe depression n (%)		OR	(95% CI)	Adjusted OR 1	(95% CI)	Adjusted OR 2	(95% CI)
	No	Yes						
Disability status								
None	4280 (93)	337 (7)	1		1		1	
At least some difficulty	383 (76)	122 (24)	4.05	3.16, 5.18	3.12	2.36, 4.14	2.91	2.13, 3.99
All	4663 (91)	459 (9)						
Physical or sexual IPV								
No	4186 (93)	308 (7)	1		1			
Yes	477 (76)	151 (24)	4.30	3.40, 5.44	3.97	3.10, 5.09	-	-
All	4663 (91)	459 (9)						
Emotional IPV								
No	4204 (92)	311 (7)	1		1			
Yes	459 (76)	148 (24)	4.36	3.40, 5.58	4.01	3.04, 5.29	-	-
All	4663 (91)	459 (9)						
	Suicidal ideation n (%)		OR	(95% CI)	Adjusted OR 1	(95% CI)	Adjusted OR 2	(95% CI)
	No	Yes						
Disability status								
None	4089 (89)	528 (11)	1		1		1	
At least some difficulty	384 (76)	121 (24)	2.44	1.98, 3.01	2.13	1.66, 2.75	1.94	1.50, 2.50
All	4473 (87)	649 (13)						
Physical or sexual IPV								
No	4087 (91)	407 (9)	1		1			
Yes	386 (61)	242 (39)	6.30	5.01, 7.92	6.04	4.80, 7.62	-	-
All	4473 (87)	649 (13)						
Emotional IPV								
No	4112 (91)	403 (9)	1		1			
Yes	361 (59)	246 (41)	6.95	5.81, 8.33	6.55	5.36, 8.00	-	-
All	4473 (87)	649 (13)						

OR: odds ratio. AOR: adjusted odds ratio. ORs and 95% confidence intervals (CI) from univariable and multivariable logistic regression models.

Adjusted OR 1: adjusted for sociodemographic covariates: woman's age, marital status, schooling, employment, religion, socioeconomic quintile, and husband's schooling, employment, and alcohol or drug use.

Adjusted OR 2: adjusted for sociodemographic covariates and preceding year physical or sexual IPV and emotional IPV.

For peer review only

Discussion

For peer review only - http://bmjopen.bmj.com/site/about/guidelines.xhtml

In a cross-sectional survey of women living in informal settlements in Mumbai, disability was more common in women over 30 years of age and with children, who were separated, divorced or widowed, and from poorer households. Women with disability had over 50% greater odds of reporting physical, sexual, or emotional IPV than non-disabled women, and disabled women and those who had experienced IPV had greater odds of anxiety, depression, and suicidal thinking. Overall, the increased odds of disabled women experiencing IPV are consistent with studies from high-income [12] [84] and low-income settings [37] [85]. These findings contribute to our understanding of a serious but insufficiently investigated public health issue [15].

Efforts should be made to identify and support disabled women unknown to healthcare and violence support services. Routine healthcare and IPV education and screening programmes need to be disability-inclusive and the vulnerability of disabled women needs to be incorporated into service provision. At screenings, disabled women should have access to safe and private spaces, away from accompanying family members who may be party to violence. Those delivering screenings may need training to consider different kinds of functional difficulty and access needs such as adapted

For peer review only

communication. Training should also incorporate issues of trust and respect, as women with disabilities can experience disbelief when reporting episodes of violence [86] [87]. Respect and service provision for older women and those who are widowed, separated, or divorced are also necessary. In our study, disabled women were more likely to be separated or divorced [10]. The ending of marriage is often viewed critically in India and the burden of fault placed on the woman [88]. Healthcare workers should be sensitive to this and be aware of disability inclusive practice, and so play a part in challenging stigma.

The mental health burden in India is high and greater treatment provision is needed [89] [90]. One-in-seven Indian people are believed to be affected by some mental disorder and the burden of depressive and anxiety disorders is believed to be greatest among women [91]. For women with disabilities, the burden may be higher [92]. Two studies from South India found that disabled women were more likely to experience comorbid depression and diabetes than non-disabled women [93] [94]. One of these found that disabled women were nearly ten times more likely to have depression than non-disabled women (AOR 9.5, CI, 2.2-40.8) [93]. Our study supports these findings:

For peer review only - http://bmjopen.bmj.com/site/about/guidelines.xhtml

disabled women had more mental health concerns even when controlling for socioeconomic conditions and experience of violence. Whether poor mental health and disability precede violence or vice-versa cannot be determined here. Comorbid mental health disorders may also result from pre-existing health conditions and be exacerbated by exposure to violence. These issues should be the subject of future research. Further, as with the elevated vulnerability to violence, this burden of mental disorder needs to be communicated to relevant stakeholders and methods to address it developed.

The intersection of poverty, inequality, and discriminatory social norms is central to both violence and mental health disorder [95] [96]. In visualising and categorising interconnected determinants, the ecological framework used in our analysis has utility. Determining the source of violence, whether at individual micro or broader macro levels, is less clear-cut. The effects of multidimensional poverty and gender in ecologies of violence have received attention in recent years [9] [97], but more research is needed, both quantitative and qualitative, to understand the place of disability and mental health in this matrix. A potential data source is India's National

For peer review only - http://bmjopen.bmj.com/site/about/guidelines.xhtml

Family Health Survey (NFHS), which has been broadened to include measures of disability [98]. The NFHS has already been used widely in IPV studies involving non-disabled women [99] [40]. It is hoped that this change will bring greater visibility to women with disabilities in investigations of violence.

Alongside improved visibility, regressive social and cultural norms must be challenged. These include the conception of disability as a personal failing or a sign of ill fate, and of disabled people as deserving of pity [100] [86], as well as patriarchal and stigmatizing attitudes towards women and mental disorder in general [101, 102], and tolerance of violence among both women and men [103]. This can be achieved, in part, through integrated inclusive interventions that incorporate anti-violence messages with other approaches, and through targeted interventions that seek to change violence-related norms directly [104] [55]. These efforts work best when addressing both men and women across all social classes and levels of the social ecology [65]. A recent targeted pilot study in Jharkhand, India that mobilised community resources through participatory learning and action groups facilitated by Accredited Social Health Activists (ASHAs) reported reductions in experience and tolerance of violence and

For peer review only - http://bmjopen.bmj.com/site/about/guidelines.xhtml

greater levels of help-seeking [105]. The study contributes to a growing body of evidence demonstrating that norms and practices that perpetuate inequities and violence can be challenged by community mobilisation and education [106]. Such interventions should work with disabled communities and activists, incorporating disability-positive messaging, to challenge discrimination at the community level and provide a more nuanced and inclusive approach to anti-violence efforts.

At a policy level, three major instruments in Indian law pertain directly to the evidence presented herein: the Protection of Women from Domestic Violence Act, 2005 (PWDVA) [107], the Rights of Persons with Disability Act, 2016 [108], and the Mental Healthcare Act, 2017 [109]. The greater scope for understanding and protection that these most recent instruments afford is a step in the right direction. The Rights of Persons with Disability Act brought India's disability policy in line with the United Nations Convention on the Rights of Persons with Disabilities (UN-CRPD) and introduced a rights-based approach towards disability [110], while the Mental Healthcare Act shifted the approach to mental health away from criminalisation towards provision of healthcare [111]. However, there were limitations in the

For peer review only - http://bmjopen.bmj.com/site/about/guidelines.xhtml

development of both [110] [112], including the Mental Healthcare Act's failure to sufficiently address stigma [113]. As for the PWDVA, India's persistently high rates of domestic violence, which have grown during the COVID-19 pandemic [114], are testament to the act's failure to effect any real change in society [115] [116]. This further demonstrates that, while policy can be an important tool for addressing attitudinal and environmental barriers, change will remain elusive without commitment to implementation supported by adequate funding and monitoring-accountability mechanisms.

Limitations

Limitations include the cross-sectional study design, which precludes determination of whether disability was an outcome of or risk factor for IPV. Use of self-report for exposure items is a further limitation, although the recall period was short, participants being asked about their experiences in the last year. Responses to questions about IPV and mental health may have been self-censored due to the sensitive nature of the questions and therefore underestimated, despite researchers being familiar figures in the community and their efforts to develop trust. The reported prevalence of IPV among all women (12% for physical or sexual and emotional IPV) was lower than at

For peer review only - http://bmjopen.bmj.com/site/about/guidelines.xhtml

national level [28] [29] and in slums [32] [33]. It was also lower among disabled women (17% for physical/sexual IPV; 16% for emotional IPV) when compared to other studies [11, 35, 36]. On the other hand, having used definitions of violence and survey instruments comparable with other studies, it may be accurate. In favour of this, the rate accords closely with a study of physical (12%), sexual (2%), and emotional IPV (8%) experienced six weeks postpartum also undertaken by SNEHA in Mumbai informal settlements [74].

Conclusion

Violence against women in India is a major and ongoing public health problem. Women with disabilities are perhaps the most marginalised of all and experience violence to a greater degree than others. This violence derives from a culture that tolerates and perpetuates the marginalisation of both women in general and women with disabilities in particular and contributes to an unacceptable physical and mental health burden. The issue requires greater attention from national and community leaders to address its causes, especially poverty and gender inequality, and to meet India's commitments to its disabled population.

For peer review only - http://bmjopen.bmj.com/site/about/guidelines.xhtml

Acknowledgments

We are grateful to the women and community guardians who agreed to contribute to the study. We thank the investigators, data collection supervisors Miheeka Vast and Manju Singh, Bhaskar Kakad for supervision, Gauri Savkur for training support, Unnati Machchhar and Shilpa Adelkar for subsequent supervision of the intervention programme, Archana Bagra and Vibhavari Bali for financial and human resources management, and Vanessa D’Souza and Shanti Pantvaidya for leadership at SNEHA.

For peer review only

Contributorship

AR, ND and DO conceived the study. ND, MW, SK, AG, and DO developed the methodology for data collection. ND, SK, and AG oversaw investigation. AR and DO curated the data and designed the analysis. AR did the analysis and wrote the first draft. ND managed the project and ND and DO acquired funding. All authors critically reviewed drafts of the manuscript and read and commented on the final version.

Competing interests.

The authors declare no competing interest.

Funding

This research was funded in part by the Wellcome Trust (206417). For the purpose of open access, the author has applied a CC BY public copyright licence to any Author Accepted Manuscript version arising from this submission. The funder had no role in study design, data collection and analysis, decision to publish, or preparation of the manuscript.

Data sharing

Data are available in Open Science Framework: Osrin D. Disability dataset. 2021.osf.io/th6a3

For peer review only

References

1. WHO. 2011. *World report on disability*. https://www.who.int/disabilities/world_report/2011/report/en/.
2. UN. 2006. *Convention on the Rights of Persons with Disabilities* (13 December 2006); A/RES/61/106, Annex I. https://www.un.org/development/desa/disabilities/convention-on-the-rights-of-persons-with-disabilities.html.
3. UNESCO. 2018. *Education and Disability: Analysis of Data from 49 Countries. Information Paper No. 49*. Institute for Statistics. http://uis.unesco.org/en/news/education-and-disability-analysis-data-49-countries.
4. Mitra, S., Posarac, A. and Vick, B. 2013. Disability and poverty in developing countries: a multidimensional study. *World Development*. 41; pp.1-18.
5. Heymann, J., Stein, M.A. and Moreno, G. 2013. *Disability and Equity at Work*. Oxford University Press, Oxford.
6. Mithen, J., Aitken, Z., Ziersch, A. and Kavanagh, A.M. 2015. Inequalities in social capital and health between people with and without disabilities. *Social Science & Medicine*. 126; pp.26-35.
7. Banks, L.M., Kuper, H. and Polack, S. 2017. Poverty and disability in low-and middle-income countries: A systematic review. *PloS One*. 12(12), p.e0189996.

For peer review only - http://bmjopen.bmj.com/site/about/guidelines.xhtml

8. Braithwaite, J., Mont, D. 2009. Disability and poverty: a survey of World Bank poverty assessments and implications. *Alter*. 3(3); pp.219-232.
9. Groce, N., Kett, M., Lang, R. and Trani, J.F. 2011. Disability and poverty: The need for a more nuanced understanding of implications for development policy and practice. *Third World Quarterly*. 32(8); pp.1493-1513.
10. Daruwalla, N., Chakravarty, S., Chatterji, S., Shah More, N., Alcock, G., Hawkes, S. and Osrin, D. 2013. Violence against women with disability in Mumbai, India: a qualitative study. *Sage Open*. 3(3); p.2158244013499144.
11. Rashid, S., Daruwalla, N., Puri, M., Hawkes, S. and Chow, J. 2012. *Count me in! Research Report: Violence Against Disabled, Lesbian, and Sex-Working Women in Bangladesh, India, and Nepal*. CREA. http://www.creaworld.org/sites/default/files/The%20Count%20Me%20In!%20Research%20Report.pdf.
12. Brownridge, D.A. 2006. Partner violence against women with disabilities: Prevalence, risk and explanations. *Violence Against Women*. 12; pp.805-822.
13. Krug, E.G., Mercy, J.A., Dahlberg, L.L. and Zwi, A.B. (eds.). 2002. *World Report on Violence and Health*. World Health Organisation. https://www.who.int/violence_injury_prevention/violence/world_report/en/.
14. Butchart, A., Mikton, C. 2014. Global Status Report on Violence Prevention. *World Health Organisation*. https://www.who.int/violence_injury_prevention/violence/status_report/2014/en/.
15. Hughes, K., Bellis, M.A., Jones, L., Wood, S., Bates, G., Eckley, L., McCoy, E., Mikton, C., Shakespeare, T. and Officer, A. 2012. Prevalence and risk of violence against adults with disabilities: a systematic review and meta-analysis of observational studies. *The Lancet*. 379(9826); pp.1621-1629.
16. García-Moreno, C., Pallitto, C., Devries, K., Stöckl, H., Watts, C. and Abrahams, N. 2013. Global and regional estimates of violence against women: prevalence and health effects of intimate partner violence and non-partner sexual violence. *World Health Organization*. https://www.who.int/reproductivehealth/publications/violence/9789241564625/en/.
17. Cieza, A., Sabariego, C., Bickenbach, J. and Chatterji, S. 2018. Rethinking disability. *BMC Medicine*. 16(1); pp.1-5.
18. Mikton, C., Maguire, H. and Shakespeare, T. 2014. A systematic review of the effectiveness of interventions to prevent and respond to violence against persons with disabilities. *Journal of Interpersonal Violence*. 29(17); pp.3207-3226.
19. Government of India. 2016a. *Disabled Persons in India: A statistical profile 2016*. Ministry of Statistics and Programme Implementation. http://mospi.nic.in/sites/default/files/publication_reports/Disabled_persons_in_India_2016.pdf.
20. Government of India. 2018. *Persons with Disabilities in India*. National Statistics Office. http://www.mospi.gov.in/sites/default/files/publication_reports/Report_583_Final_0.pdf.
21. Dandona, R., Pandey, A., George, S., Kumar, G.A. and Dandona, L. 2019. India's disability estimates: Limitations and way forward. *PloS One*. 14(9).
22. O'Keefe, P. 2009. *People with Disabilities in India: From Commitments to Outcomes*. World Bank Human Development Unit, South Asia Region. https://documents.worldbank.org/en/publication/documents-

For peer review only - http://bmjopen.bmj.com/site/about/guidelines.xhtml

reports/documentdetail/577801468259486686/people-with-disabilities-in-india-from-commitments-to-outcomes.
23. Mitra, S. and Sambamoorthi, U. 2014. Disability prevalence among adults: estimates for 54 countries and progress toward a global estimate. *Disability and Rehabilitation*. 36(11); pp.940-947.
24. Velayutham, B., Kangusamy, B. and Mehendale, S. 2017. Prevalence of Disability in Tamil Nadu, India. *The National Medical Journal of India*. 30(3); p.125.
25. Mopari, R., Garg, B., Puliyel, J. and Varughese, S. 2019. Measuring disability in an urban slum community in India using the Washington Group questionnaire. *Disability and Health Journal*. 12(2); pp.263-268.
26. Gudlavalleti, M.V.S. 2014. *The Telangana Disability Study, India Country Report*. International Centre for Evidence in Disability and London School of Hygiene and Tropical Medicine. https://www.researchgate.net/publication/280312727_Telangana_Disability_Study.
27. Saikia, N., Bora, J.K., Jasilionis, D. and Shkolnikov, V.M. 2016. Disability divides in India: evidence from the 2011 census. *PloS One*. 11(8).
28. IIPS. 2017. *National Family Health Survey 2015-16 (NFHS-4)*. International Institute for Population Sciences, Government of India, Mumbai. https://dhsprogram.com/pubs/pdf/FR339/FR339.pdf.
29. Kalokhe, A., del Rio, C., Dunkle, K., Stephenson, R., Metheny, N., Paranjape, A. and Sahay, S. 2017. Domestic violence against women in India: A systematic review of a decade of quantitative studies. *Global Public Health*. 12(4); pp.498-513.
30. Begum, S., Donta, B., Nair, S. and Prakasam, C.P. 2015. Socio-demographic factors associated with domestic violence in urban slums, Mumbai, Maharashtra, India. *The Indian Journal of Medical Research*. 141(6); p.783.
31. Dasgupta, A., Preeti, P.S., Sahoo, S.K., Biswas, D., Kumar, A. and Das, M. 2015. Domestic Violence and its Determinants: A cross-sectional study among women in a slum of Kolkata. *Indian Journal of Community Health*. 27(3); pp.334-340.
32. Gaikwad, V., Rao, D.H. 2014. A Cross-sectional Study of Domestic Violence Perpetrated by Intimate Partner against Married Women in the Reproductive Age Group in an Urban Slum Area in Mumbai. *Indian Journal of Public Health Research & Development*. 5(1); pp.49-54.
33. Pal, J., Mullick, T.H., Ahmad, S. and Yadav, A.K. 2017. Domestic Violence against Women-An Unsolved Issue: A Community Based Study in an Urban Slum of Kolkata, India. *Journal of Clinical & Diagnostic Research*. 11(10).
34. Sinha, A., Mallik, S., Sanyal, D., Dasgupta, S., Pal, D. and Mukherjee, A. 2012. Domestic violence among ever married women of reproductive age group in a slum area of Kolkata. *Indian Journal of Public Health Research & Development*. 56(1); p.31.
35. Deepak, S., Kumar, J., Santosh, B., Gornalli, S., Manikappa, P., Vyjantha, U. and Giriyappa, R. 2014. Violence against persons with disabilities in Bidar District, India. *Disability, CBR & Inclusive Development*. 25(2); pp.35-53.
36. Mohapatra, S. and Mohanty, M. 2005. *Abuse and activity limitation - A study on domestic violence against disabled women in Orissa, India*. Oxfam, India.
37. Gupta, J., Cardoso, L.F., Ferguson, G., Shrestha, B., Shrestha, P.N., Harris, C., Groce, N. and Clark, C.J. 2018. Disability status, intimate partner violence and perceived social support among married women in three districts of the Terai region of Nepal. *BMJ Global Health*. 3(5); p.e000934.

For peer review only

38. Hasan, T., Muhaddes, T., Camellia, S., Selim, N. and Rashid, S.F. 2014. Prevalence and experiences of intimate partner violence against women with disabilities in Bangladesh: Results of an explanatory sequential mixed-method study. *Journal of Interpersonal Violence*. 29(17); pp.3105-3126.
39. Puri, M., Misra, G. and Hawkes, S. 2015. Hidden voices: prevalence and risk factors for violence against women with disabilities in Nepal. *BMC Public Health*. 15(1); p.261.
40. Sabri, B., Renner, L.M., Stockman, J.K., Mittal, M. and Decker, M.R. 2014. Risk factors for severe intimate partner violence and violence-related injuries among women in India. *Women & Health*. 54(4); pp.281-300.
41. Spiwak, R., Logsetty, S., Afifi, T.O. and Sareen, J. 2015. Severe partner perpetrated burn: Examining a nationally representative sample of women in India. *Burns*. 41(8); pp.1847-1854.
42. Silverman, J.G., Decker, M.R., Saggurti, N., Balaiah, D. and Raj, A. 2008. Intimate partner violence and HIV infection among married Indian women. *JAMA*. 300(6); pp.703-710.
43. Sudha, S. and Morrison, S. 2011. Marital violence and women's reproductive health care in Uttar Pradesh, India. *Women's Health Issues*. 21(3); pp.214-221.
44. Anand, E., Unisa, S. and Singh, J. 2017. Intimate partner violence and unintended pregnancy among adolescent and young adult married women in south Asia. *Journal of Biosocial Science*. 49(2); pp.206-21.
45. Dhar, D., McDougal, L., Hay, K., Atmavilas, Y., Silverman, J., Triplett, D. and Raj, A. 2018. Associations between intimate partner violence and reproductive and maternal health outcomes in Bihar, India: a cross-sectional study. *Reproductive Health*. 15(1); p.109.
46. Jejeebhoy, S.J., Santhya, K.G. and Acharya, R. 2013. Physical and sexual violence and symptoms of gynaecological morbidity among married young women in India. *Global Public Health*. 8(10); pp.1151-1167.
47. Chandra, P.S., Satyanarayana, V.A. and Carey, M.P. 2009. Women reporting intimate partner violence in India: Associations with PTSD and depressive symptoms. *Archives of Women's Mental Health*. 12(4); p.203.
48. Chowdhury, A.N., Brahma, A., Banerjee, S. and Biswas, M.K. 2009. Pattern of domestic violence amongst non-fatal deliberate self-harm attempters: A study from primary care of West Bengal. *Indian Journal of Psychiatry*. 51(2); p.96.
49. Maselko, J. and Patel, V. 2008. Why women attempt suicide: the role of mental illness and social disadvantage in a community cohort study in India. *Journal of Epidemiology & Community Health*. 62(9); pp.817-822.
50. Suryanarayana, M., Agrawal, A. and Prabhu, K.S. 2011. *Inequality adjusted human development index for India's states*. United Nations Development Programme, India.
51. MCGM. 2020. *Mumbai Census Population*. Municipal Corporation of Greater Mumbai. https://portal.mcgm.gov.in/irj/portal/anonymous/qlvitalstatsreport?guest_user=english.
52. Chandramouli, C. 2011. *Housing stock, amenities and assets in slums - Census 2011*. New Delhi: Office of the Registrar General and Census Commissioner.
53. Ezeh, A., Oyebode, O., Satterthwaite, D., Chen, Y.F., Ndugwa, R., Sartori, J., Mberu, B., Melendez-Torres, G.J., Haregu, T., Watson, S.I. and Caiaffa, W. 2017. The history, geography, and sociology of slums and the health problems of people who live in slums. *The Lancet*. 389(10068); pp.547-558.

For peer review only

For peer review only - http://bmjopen.bmj.com/site/about/guidelines.xhtml

54. UN-Habitat. 2017. *Human Rights in Cities Handbook Series Volume 1: The Human Rights-Based Approach to Housing and Slum Upgrading*. https://www.ohchr.org/Documents/Issues/Housing/InformalSettlements/UNHABITAT_HumanRights-BasedApproch.pdf.
55. Daruwalla, N., Machchhar, U., Pantvaidya, S., D'Souza, V., Gram, L., Copas, A. and Osrin, D. 2019. Community interventions to prevent violence against women and girls in informal settlements in Mumbai: the SNEHA-TARA pragmatic cluster randomised controlled trial. *Trials*. 20(1); p.743.
56. Daruwalla, N., Kanougiya, S., Gupta, A., Gram, L. and Osrin, D. . 2020. Prevalence of domestic violence against women in informal settlements in Mumbai, India: a cross-sectional survey. *BMJ Open*. 10:e042444. DOI: 10.1136/bmjopen-2020-042444.
57. Kalokhe, A.S., Stephenson, R., Kelley, M.E., Dunkle, K.L., Paranjape, A., Solas, V., Karve, L., del Rio, C. and Sahay, S. 2016. The development and validation of the Indian family violence and control scale. *PLoS One*. 11(1); p.e0148120.
58. Johnson, H., Ollus, N. and Nevala, S. 2008. *Violence Against Women: An International Perspective*. Springer Science & Business Media, New York.
59. García-Moreno, C., Jansen, H.A., Ellsberg, M., Heise, L. and Watts, C. 2005. WHO multi-country study on women's health and domestic violence against women. *World Health Organization*. https://www.who.int/reproductivehealth/publications/violence/24159358X/en/.
60. Washington Group on Disability Statistics. 2009. *Understanding and interpreting disability as measured using the WG short set of questions*. https://www.cdc.gov/nchs/data/washington_group/meeting8/interpreting_disability.pdf.
61. Spitzer, R.L., Kroenke, K., Williams, J.B. and Löwe, B. 2006. A brief measure for assessing generalized anxiety disorder: the GAD-7. *Archives of Internal Medicine*. 166(10); pp.1092-1097.
62. Kroenke, K., Spitzer, R.L. and Williams, J.B. 2001. The PHQ-9: validity of a brief depression severity measure. *Journal of General Internal Medicine*. 16(9); pp.606-613.
63. Kroenke, K. and Spitzer, R.L. 2010. *Instruction manual: Instructions for patient health questionnaire (PHQ) and GAD-7 measures*. https://www.phqscreeners.com/.
64. Heise, L. 1998. Violence against women: An integrated, ecological framework. *Violence Against Women*. 4(3); pp.262-290.
65. Heise, L. 2011. *What works to prevent partner violence? An evidence overview*. STRIVE. http://strive.lshtm.ac.uk/system/files/attachments/What%20works%20to%20prevent%20partner%20violence.pdf.
66. Jain, S., Varshney, K., Vaid, N.B., Guleria, K., Vaid, K. and Sharma, N. 2017. A hospital-based study of intimate partner violence during pregnancy. *International Journal of Gynecology & Obstetrics*. 137(1); pp.8-13.
67. Mahapatro, M., Gupta, R.N. and Gupta, V. 2012. The risk factor of domestic violence in India. *Indian journal of Community Medicine: Official Publication of Indian Association of Preventive & Social Medicine*. 37(3); p.153.
68. Shrivastava, P.S. and Shrivastava, S.R. 2013. A study of spousal domestic violence in an urban slum of Mumbai. *International Journal of Preventive Medicine*. 4(1); p.27.
69. Biswas, C.S. 2017. Spousal Violence against Working Women in India. *Journal of Family Violence*. 32(1); pp.55-67.
70. Bhattacharya, H. 2015. Spousal violence and Women's employment in India. *Feminist Economics*. 21(2); pp.30-52.

For peer review only - http://bmjopen.bmj.com/site/about/guidelines.xhtml

For peer review only

71. Weitzman, A. 2014. Women's and men's relative status and intimate partner violence in India. *Population and Development Review*. 40(1); pp.55-75.
72. Rapp, D., Zoch, B., Khan, M.M.H., Pollmann, T. and Krämer, A. 2012. Association between gap in spousal education and domestic violence in India and Bangladesh. *BMC Public Health*. 12(1); p.467.
73. Kimuna, S.R., Djamba, Y.K., Ciciurkaite, G. and Cherukuri, S. 2013. Domestic violence in India: Insights from the 2005-2006 national family health survey. *Journal of Interpersonal Violence*. 28(4); pp.773-807.
74. Das, S., Bapat, U., More, N.S., Alcock, G., Joshi, W., Pantvaidya, S. and Osrin, D. 2013. Intimate partner violence against women during and after pregnancy: a cross-sectional study in Mumbai slums. *BMC Public Health*. 13(1); p.817.
75. Wagman, J.A., Donta, B., Ritter, J., Naik, D.D., Nair, S., Saggurti, N., Raj, A. and Silverman, J.G. 2018. Husband's alcohol use, intimate partner violence, and family maltreatment of low-income postpartum women in Mumbai, India. *Journal of Interpersonal Violence*. 33(14); pp.2241-2267.
76. Huria, K.A., Deepti, D., Lajya, D. and Sunder, S.S. 2005. Domestic violence in pregnancy in North Indian women. *Indian Journal of Medical Sciences*. 59(5); pp.195-199.
77. Chibber, K.S., Krupp, K., Padian, N. and Madhivanan, P. 2012. Examining the determinants of sexual violence among young, married women in Southern India. *Journal of Interpersonal Violence*. 27(12); pp.2465-2483.
78. Dalal, K., Lindqvist, K. 2012. A national study of the prevalence and correlates of domestic violence among women in India. *Asia Pacific Journal of Public Health*. 24(2); pp.265-277.
79. Krishnan, S. 2005. Gender, caste, and economic inequalities and marital violence in rural South India. *Health Care for Women International*. 26(1); pp.87-99.
80. Victora, C.G., Huttly, S.R., Fuchs, S.C. and Olinto, M.T. 1997. The role of conceptual frameworks in epidemiological analysis: a hierarchical approach. *International Journal of Epidemiology*. 26(1); pp.224-227.
81. Filmer, D., Pritchett, L.H. 2001. Estimating wealth effects without expenditure data—or tears: an application to educational enrollments in states of India. *Demography*. 38(1); pp.115-132.
82. Vyas, S. and Kumaranayake, L. 2006. Constructing socio-economic status indices: how to use principal components analysis. *Health Policy and Planning*. 21(6); pp.459-468.
83. WHO. 2001. *Putting Women First: Ethical and Safety Recommendations for Research on Domestic Violence against Women*. Department of Gender and Women's Health, Family and Community Health. https://www.who.int/gender-equity-rights/knowledge/who_fch_gwh_01.1/en/.
84. Hahn, J.W., McCormick, M.C., Silverman, J.G., Robinson, E.B. and Koenen, K.C. 2014. Examining the impact of disability status on intimate partner violence victimization in a population sample. *Journal of Interpersonal Violence*. 29(17); pp.3063-3085.
85. Van Der Heijden, I. and Dunkle, K. 2017. *What Works Evidence Review: Preventing violence against women and girls with disabilities in lower-and middle-income countries (LMICs)*. UKAIDS. https://www.whatworks.co.za/resources/evidence-reviews/item/349-what-works-evidence-review-preventing-violence-against-women-and-girls-with-disabilities-in-lower-and-middle-income-countries-lmics.
86. Ghosh, N. 2016. Negotiating Femininity: Lived Experiences of Women with Locomotor Disabilities in Bengal. In: Ghosh, N. (ed.). *Interrogating Disability in India*. 2016 pp. 127-144, Springer, New Delhi.

For peer review only - http://bmjopen.bmj.com/site/about/guidelines.xhtml

87. Sharma, S. and Sivakami, M. 2019. Sexual and reproductive health concerns of persons with disability in india: An issue of deep-rooted silence. *Journal of Biosocial Science*. 51(2); pp.225-243.
88. Asthana, A. 2017. Women in Distress: A Study on the Widowed, Divorced and Separated Women in Surguja (Chhattisgarh, India). *Divorced and Separated Women in Surguja (Chhattisgarh, India)*. https://dx.doi.org/10.2139/ssrn.3223334.
89. Kallakuri, S., Devarapalli, S., Tripathi, A.P., Patel, A. and Maulik, P.K. 2018. Common mental disorders and risk factors in rural India: baseline data from the SMART mental health project. *BJPsych Open*. 4(4); pp.192-198.
90. Kar, S.K., Sharma, E., Agarwal, V., Singh, S.K., Dalal, P.K., Singh, A., Gopalkrishna, G. and Rao, G.N. 2018. Prevalence and pattern of mental illnesses in Uttar Pradesh, India: Findings from the National Mental Health Survey 2015–16. *Asian Journal of Psychiatry*. 38; pp.45-52.
91. Sagar, R., Dandona, R., Gururaj, G., Dhaliwal, R.S., Singh, A., Ferrari, A., Dua, T., Ganguli, A., Varghese, M., Chakma, J.K. and Kumar, G.A. 2020. The burden of mental disorders across the states of India: The Global Burden of Disease Study 1990–2017. *The Lancet Psychiatry*. 7(2); pp.148-161.
92. Dembo, R.S., Mitra, M. and McKee, M. 2018. The psychological consequences of violence against people with disabilities. *Disability and Health Journal*. 11(3); pp.390-397.
93. Gudlavalleti, M.V.S., John, N., Allagh, K., Sagar, J., Kamalakannan, S., Ramachandra, S.S 2014. Access to health care and employment status of people with disabilities in South India, the SIDE (South India Disability Evidence) study. *BMC Public Health*. 14(1); p.1125.
94. Murthy, G.V.S., John, N., Sagar, J. . 2014. Reproductive health of women with and without disabilities in South India, the SIDE study (South India Disability Evidence) study: a case control study. *BMC Women's Health*. 14(1); p.146.
95. Bhattacharya, A., Camacho, D., Kimberly, L.L. and Lukens, E.P. 2019. Women's experiences and perceptions of depression in India: A metaethnography. *Qualitative Health Research*. 29(1); pp.80-95.
96. Malhotra, S. and Shah, R. 2015. Women and mental health in India: An overview. *Indian Journal of Psychiatry*. 57(Suppl 2); p.S205.
97. Subbaraman, R., Nolan, L., Shitole, T., Sawant, K., Shitole, S., Sood, K., Nanarkar, M., Ghannam, J., Betancourt, T.S., Bloom, D.E. and Patil-Deshmukh, A. 2014. The psychological toll of slum living in Mumbai, India: a mixed methods study. *Social Science & Medicine*. 119; pp.155-169.
98. Government of India. 2019. *Conducting the NFHS/Annual Health Surveys*. Ministry of Health and Family Welfare. https://pib.gov.in/PressReleaseIframePage.aspx?PRID=1576629.
99. Ahmad, J., Khan, N. and Mozumdar, A. 2019. Spousal Violence Against Women in India: A Social–Ecological Analysis Using Data from the National Family Health Survey 2015 to 2016. *Journal of Interpersonal Violence*. DOI: 10.1177/0886260519881530.
100. Ghai, A. 2019. Rethinking Disability in India. *Taylor & Francis*. London.
101. Goel, R. 2005. Sita's trousseau: Restorative justice, domestic violence, and South Asian culture. *Violence Against Women*. 11; 639-665.
102. Ahmed-Ghosh, H. 2004. Chattels of society: Domestic violence in India. *Violence against women*. 10; 94-118.

For peer review only - http://bmjopen.bmj.com/site/about/guidelines.xhtml

103. Rowan, K., Mumford, E. and Clark, C.J. . 2018. Is women's empowerment associated with help-seeking for spousal violence in India? *Journal of interpersonal violence*. 33; 1519-1548.
104. WHO. 2009. *Changing cultural and social norms supportive of violence behaviour. Series of briefings on violence prevention: the evidence*. https://www.who.int/violence_injury_prevention/violence/norms.pdf.
105. Nair, N., Daruwalla, N., Osrin, D., Rath, S., Gagrai, S., Sahu, R., Pradhan, H., De, M., Ambavkar, G., Das, N. and Dungdung, G.P. . 2020. Community mobilisation to prevent violence against women and girls in eastern India through participatory learning and action with women's groups facilitated by accredited social health activists: a before-and-after pilot study. *BMC International Health and Human Rights*. 20(1), pp.1-12.
106. Ellsberg, M., Arango, D.J., Morton, M., Gennari, F., Kiplesund, S., Contreras, M. and Watts, C. . 2015. Prevention of violence against women and girls: what does the evidence say? *The Lancet*. 385(9977), pp.1555-1566.
107. Government of India. 2005. *The Protection of Women from Domestic Violence Act, 2005*. Ministry of Law and Justice. https://legislative.gov.in/actsofparliamentfromtheyear/protection-women-domestic-violence-act-2005.
108. Government of India. 2016b. *The Rights of Persons with Disability Act, 2016*. Ministry of Law and Justice. http://legislative.gov.in/actsofparliamentfromtheyear/rights-persons-disabilities-act-2016.
109. Government of India. 2017. *The Mental Healthcare Act, 2017*. Ministry of Law and Justice. https://www.prsindia.org/uploads/media/Mental%20Health/Mental%20Healthcare%20Act,%202017.pdf.
110. Math, S.B., Gowda, G.S., Basavaraju, V., Manjunatha, N., Kumar, C.N., Philip, S. and Gowda, M. 2019. The rights of persons with disability act, 2016: Challenges and opportunities. *Indian Journal of Psychiatry*. 61(Suppl 4); p.S809.
111. Mishra, A. and Galhotra, A. 2018. Mental healthcare Act 2017: Need to wait and watch. *International Journal of Applied and Basic Medical Research*. 8(2), p.67.
112. Balakrishnan, A., Kulkarni, K., Moirangthem, S., Kumar, C.N., Math, S.B. and Murthy, P. 2019. The rights of persons with disabilities Act 2016: Mental health implications. *Indian Journal of Psychological Medicine*. 41(2), pp.119-125.
113. Duffy, R.M., Gulati, G., Kasar, N., Paralikar, V., Narayan, C.L., Desousa, A., Goyal, N. and Kelly, B.D. 2019. Stigma, inclusion and India's Mental Healthcare Act 2017. *Journal of Public Mental Health*. Vol. 18 No. 3, pp. 199-205.
114. Maji, S., Bansod, S. and Singh, T. 2021. Domestic violence during COVID-19 pandemic: The case for Indian women. *Journal of Community & Applied Social Psychology*. https://doi.org/10.1002/casp.2501.
115. Manjoo, R. 2014. Report of the Special Rapporteur on violence against women, its causes and consequences. *United Nations Human Rights Council*. Twenty-sixth session. https://www.ohchr.org/en/hrbodies/hrc/regularsessions/session26/documents/a-hrc-26-38-add1_en.doc.
116. Tulsyan, A. 2016. Protection of Women from Domestic Violence Act 2005: Lessons from a Decade of Implementation. *Oxfam India*. https://www.oxfamindia.org/sites/default/files/Protection-of-women-from-domestic-violence-act-2005.pdf.

For peer review only - http://bmjopen.bmj.com/site/about/guidelines.xhtml

For peer review only

For peer review only - http://bmjopen.bmj.com/site/about/guidelines.xhtml

STROBE Statement—Checklist of items that should be included in reports of ***cross-sectional studies***

	Item No	Recommendation	Page No
**Title and abstract**	1	(*a*) Indicate the study's design with a commonly used term in the title or the abstract	1
		(*b*) Provide in the abstract an informative and balanced summary of what was done and what was found	2
**Introduction**			
Background/rationale	2	Explain the scientific background and rationale for the investigation being reported	4-6
Objectives	3	State specific objectives, including any prespecified hypotheses	6-7
**Methods**			
Study design	4	Present key elements of study design early in the paper	7
Setting	5	Describe the setting, locations, and relevant dates, including periods of recruitment, exposure, follow-up, and data collection	7
Participants	6	(*a*) Give the eligibility criteria, and the sources and methods of selection of participants	8
Variables	7	Clearly define all outcomes, exposures, predictors, potential confounders, and effect modifiers. Give diagnostic criteria, if applicable	8-10
Data sources/ measurement	8*	For each variable of interest, give sources of data and details of methods of assessment (measurement). Describe comparability of assessment methods if there is more than one group	*8*
Bias	9	Describe any efforts to address potential sources of bias	11
Study size	10	Explain how the study size was arrived at	8
Quantitative variables	11	Explain how quantitative variables were handled in the analyses. If applicable, describe which groupings were chosen and why	10
Statistical methods	12	(*a*) Describe all statistical methods, including those used to control for confounding	11
		(*b*) Describe any methods used to examine subgroups and interactions	
		(*c*) Explain how missing data were addressed	
		(*d*) If applicable, describe analytical methods taking account of sampling strategy	
		(*e*) Describe any sensitivity analyses	
**Results**			
Participants	13*	(a) Report numbers of individuals at each stage of study—eg numbers potentially eligible, examined for eligibility, confirmed eligible, included in the study, completing follow-up, and analysed	12
		(b) Give reasons for non-participation at each stage	12
		(c) Consider use of a flow diagram	
Descriptive data	14*	(a) Give characteristics of study participants (eg demographic, clinical, social) and information on exposures and potential confounders	12-13
		(b) Indicate number of participants with missing data for each variable of interest	
Outcome data	15*	Report numbers of outcome events or summary measures	14-19

For peer review only

Main results	16	(*a*) Give unadjusted estimates and, if applicable, confounder-adjusted estimates and their precision (eg, 95% confidence interval). Make clear which confounders were adjusted for and why they were included	14-19
		(*b*) Report category boundaries when continuous variables were categorized	
		(*c*) If relevant, consider translating estimates of relative risk into absolute risk for a meaningful time period	
Other analyses	17	Report other analyses done—eg analyses of subgroups and interactions, and sensitivity analyses	
**Discussion**			
Key results	18	Summarise key results with reference to study objectives	20
Limitations	19	Discuss limitations of the study, taking into account sources of potential bias or imprecision. Discuss both direction and magnitude of any potential bias	23
Interpretation	20	Give a cautious overall interpretation of results considering objectives, limitations, multiplicity of analyses, results from similar studies, and other relevant evidence	20-23
Generalisability	21	Discuss the generalisability (external validity) of the study results	24
**Other information**			
Funding	22	Give the source of funding and the role of the funders for the present study and, if applicable, for the original study on which the present article is based	26

*Give information separately for exposed and unexposed groups.

Note: An Explanation and Elaboration article discusses each checklist item and gives methodological background and published examples of transparent reporting. The STROBE checklist is best used in conjunction with this article (freely available on the Web sites of PLoS Medicine at http://www.plosmedicine.org/, Annals of Internal Medicine at http://www.annals.org/, and Epidemiology at http://www.epidem.com/). Information on the STROBE Initiative is available at www.strobe-statement.org.

For peer review only

BMJ Open

Intimate partner violence against women with disability and associated mental health concerns: a cross-sectional survey in Mumbai, India

Journal:	*BMJ Open*
Manuscript ID	bmjopen-2021-056475.R1
Article Type:	Original research
Date Submitted by the Author:	08-Dec-2021

Complete List of Authors:	Riley, Andrew; University of Liverpool, Institute of Population Health<br>Daruwalla, Nayreen; SNEHA<br>Kanougiya, Suman; Tata Institute of Social Sciences (TISS)<br>Gupta, Apoorwa; SNEHA<br>Wickenden, Mary; University of Sussex<br>Osrin, David; University College London

**Primary Subject Heading**:	Public health
Secondary Subject Heading:	Mental health, Global health
Keywords:	MENTAL HEALTH, PUBLIC HEALTH, SOCIAL MEDICINE

SCHOLARONE™
Manuscripts

I, the Submitting Author has the right to grant and does grant on behalf of all authors of the Work (as defined in the below author licence), an exclusive licence and/or a non-exclusive licence for contributions from authors who are: i) UK Crown employees; ii) where BMJ has agreed a CC-BY licence shall apply, and/or iii) in accordance with the terms applicable for US Federal Government officers or employees acting as part of their official duties; on a worldwide, perpetual, irrevocable, royalty-free basis to BMJ Publishing Group Ltd ("BMJ") its licensees and where the relevant Journal is co-owned by BMJ to the co-owners of the Journal, to publish the Work in this journal and any other BMJ products and to exploit all rights, as set out in our licence.

The Submitting Author accepts and understands that any supply made under these terms is made by BMJ to the Submitting Author unless you are acting as an employee on behalf of your employer or a postgraduate student of an affiliated institution which is paying any applicable article publishing charge ("APC") for Open Access articles. Where the Submitting Author wishes to make the Work available on an Open Access basis (and intends to pay the relevant APC), the terms of reuse of such Open Access shall be governed by a Creative Commons licence – details of these licences and which Creative Commons licence will apply to this Work are set out in our licence referred to above.

Other than as permitted in any relevant BMJ Author's Self Archiving Policies, I confirm this Work has not been accepted for publication elsewhere, is not being considered for publication elsewhere and does not duplicate material already published. I confirm all authors consent to publication of this Work and authorise the granting of this licence.

For peer review only

Intimate partner violence against women with disability and associated mental health concerns: a cross-sectional survey in Mumbai, India

Authors

1. Andrew Riley *Corresponding author*

   Institute of Population Health, University of Liverpool, Liverpool, L69 3BX, UK

   andrew.riley@liverpool.ac.uk

2. Nayreen Daruwalla

   Program on Prevention of Violence Against Women and Children, SNEHA, Mumbai, Maharashtra, 400017, India

   nayreen@snehamumbai.org

3. Suman Kanougiya

   School of Health Systems Studies (SHSS), Tata Institute of Social Sciences (TISS), Mumbai, Maharashtra, India

   suman.kanougiya@gmail.com

4. Apoorwa Deepak Gupta

   Program on Prevention of Violence Against Women and Children, SNEHA, Mumbai, Maharashtra, 400017, India

   apoorwa.gupta@snehamumbai.org

5. Mary Wickenden

   Institute of Development Studies, University of Sussex, Library Rd, Falmer, Brighton BN1 9RE, UK

   M.Wickenden@ids.ac.uk

6. David Osrin

For peer review only

Institute for Global Health, University College London,

London, WC1N IEH, UK

d.osrin@ucl.ac.uk

For peer review only

Abstract

Objectives

The risk of intimate partner violence (IPV) against women with disability is believed to be high. We aimed to compare the prevalence of past-year IPV against women with and without functional difficulties in urban informal settlements, to review its social determinants, and to explore its association with mental health.

Design

Cross-sectional survey

Setting

Fifty clusters within four informal settlements.

Participants

5122 women aged 18 to 49 years.

Primary and secondary outcome measures

We used the Washington Group Short Set of Questions to assess functional difficulties. IPV in the past year was described by binary composites of questions about physical, sexual, and emotional violence. We screened for symptoms of depression using the Patient Health Questionnaire 9 and of anxiety using the Generalized Anxiety Disorder 7. Multivariable logistic regression models examined associations between functional difficulties, IPV, and mental health.

Results

The 10% of participants who screened positive for functional disability had greater odds of experiencing physical or sexual IPV (Adjusted Odds Ratio (AOR) 1.68, 95% CI 1.23, 2.29) and emotional IPV (1.52, CI 1.16, 2.00) than women who screened negative. Women who screened positive for functional disability had greater odds than women who screened negative of symptoms suggesting moderate or severe anxiety (AOR 2.50, CI 1.78, 3.49), depression (2.91, CI 2.13, 3.99), and suicidal thinking (AOR 1.94, CI 1.50, 2.50).

Conclusions

The burden of IPV fell disproportionately on women with functional difficulties, who were also more likely to screen positive for common mental disorder. Public health initiatives need to respond at local and national levels to address the overlapping and mutually reinforcing determinants of violence, while existing policy needs to be better utilised to ensure protection for the most vulnerable.

For peer review only

Keywords

Intimate partner violence; Domestic violence; Disability; India; Poverty areas

Strengths and limitations of this study

- Data came from a large cross-sectional survey of women in informal settlements.
- Data were collected by an organisation providing support for survivors of intimate partner violence.
- The study addresses a neglected area of research: the experience of disabled women in India.
- Functional difficulties were self-reported and not the primary focus of the survey.
- The cross-sectional nature of the study limits the possibility of causal inference.

For peer review only - http://bmjopen.bmj.com/site/about/guidelines.xhtml

Background

Approximately 15% of the world's population, around one billion people, live with a physical, intellectual, sensory, or mental health impairment that affects their daily lives and long-term health and wellbeing [1]. Disability is the product of attitudinal and environmental barriers that limit persons with functional difficulties from fully participating in society [2][1]. These barriers mean that, compared with non-disabled people, people with disability are less likely to be educated [3], financially secure [4], employed [5], have access to health services [1], have their health needs met [1, 6], and have their social needs met [7-9].

The double burden on women and girls with disability means that they are among the more marginalised and vulnerable in society, particularly when this burden intersects with multidimensional poverty [1]. Lacking physical and financial independence or access to socially inclusive services, disabled women who depend on partners and

[1] In this paper our approach to disability is in line with the social model which identifies disability as a social creation, 'a relationship between people with impairment and a disabling society' (Shakespeare, 2006, p.198), distinct from medical and individual models of disability. We therefore use the term 'disabled women', which denotes this position, interchangeably with 'women with disabilities'.

For peer review only - http://bmjopen.bmj.com/site/about/guidelines.xhtml

families for support are vulnerable to further social marginalisation and isolation and are at higher risk of physical violence and sexual abuse [10, 11], as well as other forms of abuse such as neglect, exploitation, and coercive control [12]. Irrespective of disability, violence against women occurs most commonly in the home, in the form of intimate partner violence (IPV) or non-partner domestic violence [13, 14]. Women with disability are believed to be at greater risk of these and other forms of violence. However, a dearth of population-level research [15], and the typically hidden nature of violence [16], mean that little is known of how disability status may lead to additional risks of IPV. Such research is important for informing IPV prevention programmes [17, 18], and for meeting the objectives of the United Nations Sustainable Development Goals.

In India, the 2011 national census [19] and the 2018 National Sample Survey (NSS) [20] both estimated disability prevalence at 2.2% (26.8 million people), although these are considered underestimates due to proxy reporting and insufficient consideration of disability [21, 22]. Other national estimates range from 8% by the World Bank [22], to 25% by the World Health Survey [1, 23]. More local estimates reflect different

For peer review only

approaches to classification and include 1.6% in Tamil Nadu State [24], 1.9% in a Delhi slum community [25] and 12.2% in Telangana State [26]. Disability is most common among older, rural women and members of scheduled tribes and scheduled castes [20, 27], while prevalence increases with age, among lower-socioeconomic groups and those without employment / the unemployed [24, 26]. Disabled women often report lower rates of marriage [20, 26], and higher rates of widowhood, separation, and divorce [20, 24].

The ubiquity of violence against women in India has been well reported in both popular media and population-level research. The fourth National Family Health Survey (NFHS-4) reported that 30% of married women had experienced physical, sexual, or emotional violence in their lifetime and 26% in the past year [28]. Similar rates were reported in a nationally representative systematic review [29] and in urban and slum settings [30-34]. Violence against women with disabilities in India is less documented: to date, only three major studies to date have reported on it at population level. A survey in Mumbai involving women aged 15-49 years with functional impairments (visual, locomotor, hearing) found that 20% of 123 ever-married disabled women had

For peer review only - http://bmjopen.bmj.com/site/about/guidelines.xhtml

experienced past-year physical IPV, 23% emotional IPV (being insulted, humiliated or threatened), and 10% sexual IPV. Violence perpetrated by people other than intimate partners was experienced by 18% of ever-married and 23% of unmarried women, most commonly by mothers-in-law in the marital family and brothers in the natal [11].

In Karnataka, of 70 female interviewees with disability, 73% reported ‘significant’ violence (versus ‘rare’ or ‘occasional’) and 23% sexual violence in the previous 12 months, while 59% reported generalised violence and 10% sexual violence in their childhood. Around half of this violence was perpetrated by family members (46%) or by people outside the family (54%) [35]. In a study involving 729 women in Odisha State, 48% of those with learning difficulties and 23% with physical impairments reported experiencing domestic violence [36]. There are a number of limitations to these studies, notably their small sample sizes and, in the latter two, their imprecision in defining violence and the use of convenience sampling [35, 36]; however, their findings are consistent with other reports from the wider South Asia region [37-39].

For peer review only

For all women, violence increases the risk of serious injury and mortality [40] [41], HIV and sexually transmitted infections [42, 43], unintended pregnancy [44], reproductive health morbidity [45, 46] and a range of mental health concerns [47-49]. Among women with disabilities in India who have experienced violence, high levels of severe mental distress, depression, suicidal ideation and attempted suicide have been documented [11].

Given its impact on physical and mental health, as well as being a matter of individual and social justice, violence against disabled women is largely absent from the literature. This absence limits the capacity of health planners and policymakers to act, while also contributing to the ongoing marginalisation of disabled communities, experiences, and concerns.

Objectives

This study sought to address the gap in understanding of the sociodemographic circumstances of women with disabilities in urban India and their experience of intimate partner violence. We aimed to identify associations between individual

For peer review only - http://bmjopen.bmj.com/site/about/guidelines.xhtml

socioeconomic determinants and disability in women aged 18 to 49 years living in informal settlements in Mumbai and to test the hypothesis that women with functional difficulty/disability are more likely to have experienced recent intimate partner violence and have higher rates of common mental disorders.

Methods

Setting

Maharashtra ranks fourth among Indian states in the human development index [50], and women tend to have higher than the national averages on indicators of literacy, marriage age and workforce participation. Mumbai, Maharashtra's capital, has approximately 12.7 million residents, 41% of whom live in informal settlements [51, 52]. These are characterised by overcrowding, lack of tenure and insufficient living space, housing durability, and water and sanitation facilities [53, 54]. Women in these disadvantaged communities typically depend on relatives for housing, making them vulnerable to homelessness, poverty and violence [54].

Design

For peer review only - http://bmjopen.bmj.com/site/about/guidelines.xhtml

We used data collected by SNEHA (Society for Nutrition, Education and Health Action), a non-government organization working in Mumbai's disadvantaged communities to improve the health of women and children and address the burden of violence. The dataset came from a survey conducted before the implementation of a community-based intervention to prevent violence against women and girls through individual volunteers and groups [55]. The survey included 50 clusters of equal size, each of 500 dwellings, covering a population of 77,000 in four areas within major informal settlements.

Data collection

Data collection has been described in detail elsewhere [56]. Clusters were mapped and homes visited to identify potential participants. Criteria for inclusion were women aged 18-49 years resident in a study cluster, who consented to interview. One woman per household was sampled (around 100 participants per cluster). Data collectors visited participants to explain the survey, discuss the process, answer questions, and provide a participant information sheet. Signed consent to interview was obtained and participants were assured of data confidentiality.

For peer review only - http://bmjopen.bmj.com/site/about/guidelines.xhtml

Sixteen women interviewers collected data. They were graduates who had not worked in the mental health field and were selected after application and interview through open recruitment. They received three months of training, which included three days of training facilitated by an expert on disability awareness and the Washington Group Short Set of questions. For participants with visual impairment, the interviews were oral, as they were for all participants. For participants with hearing impairment, a communications specialist accompanied the interviewer. For participants with learning difficulties, we developed an alternative interview in a simple visual format that was used 14 times during the survey (see Supplementary File 1).

Participants were asked about demographic characteristics, socioeconomic indicators, their health, wellbeing, functional difficulty and disability status, common mental disorders, their experience of emotional, physical, and sexual domestic violence, injuries, help-seeking, support and disclosure, and spousal drug and alcohol use. All participant information was confidential and no names or identifiable details appear in outputs or analyses. Sources of questions included the National Family Health Survey (NFHS-4) [28], the Indian Family Violence and Control Scale [57], the

For peer review only - http://bmjopen.bmj.com/site/about/guidelines.xhtml

International Violence Against Women Survey [58] and the WHO multi-country study [59]. Questions were translated where necessary, back-translated, piloted, and refined (see Supplementary File 2).

Sample size

An estimate of prevalence from a cross-sectional sample of 5000 in a population of 125,000 would have a precision of around 1%. Within this, a comparison of two categories of determinant for 100 participants in each of 50 clusters would provide 80% power at 5% significance level to detect a difference of 6% in prevalence estimates of 10-20%.

Variables

Disability was assessed using the Washington Group Short Set of Questions (WG-SS), based on the International Classification of Functioning, Disability and Health (ICF) model of disability [60]. The questions cover six functional domains (*seeing, hearing, walking, memory, self-care, speaking),* with four categories of response (*no difficulty, some difficulty, a lot of difficulty, cannot do at all*) [60]. For this analysis, a participant was classified as having disability if they reported ‘some difficulty’ in at least

For peer review only - http://bmjopen.bmj.com/site/about/guidelines.xhtml

one domain. This cut-off is one of several possible approaches and has been used in a similar setting [37].

Definitions of violence follow the WHO Multi-Country Study [59] and are discussed in detail elsewhere [56]. We screened for anxiety, depression, and suicidal thinking as common mental disorders. Anxiety was screened for using the Generalized Anxiety Disorder 7-item (GAD-7) scale [61]. Depression was screened for using the Patient Health Questionnaire (PHQ-9) [62]. For both instruments, a cut-off score of ≥10 has been used as suggestive of clinically relevant conditions requiring further assessment [63]. In this study, variables were coded 'yes' for depression and anxiety if responses were recorded as moderate or severe. Suicidal thinking was coded 'yes' for lifetime positive responses.

Covariates corresponded to determinants of risk for IPV identified from the wider body of Indian IPV research undertaken in comparable settings with women whose disability status was not identified. Covariates were classified by level of occurrence, following Heise's ecologic framework for violence against women [64, 65]. This approach

For peer review only

conceptualises risk of violence as a function of mutually interacting personal, situational, and sociocultural factors across individual, relationship, household, community, and society levels.

We hypothesised that a woman's odds of experiencing IPV would increase with age [66] [67] [68], number of children [66] [40], and employment outside the home [69] [70] [71], and would decrease with years of education [32] [72] [40]. Partner use of alcohol or drugs was hypothesised to increase the odds [30] [73] [74] [67] [75], while no clear relationship between IPV and partner level of education was predicted [33] [76] [66] [77]. Lower socioeconomic status was considered as potentially increasing the odds of IPV [11] [33] [34] [40] [74] [78], as were faith [67] [78] and caste [79] [67], due to the association of some groups with lower socioeconomic status.

Associations between common mental disorders and sociodemographic characteristics were hypothesised to conform to a similar pattern due to their close association with IPV. Hypothetical determinants were modelled as independent variables in both univariable and multivariable analyses [80]. At the level of individual

For peer review only - http://bmjopen.bmj.com/site/about/guidelines.xhtml

women, age-group, marital status, parity, education and employment were entered as categorical variables. Categories of age and schooling were based on those used in other studies [37] [40] [42] [45]. Individual employment categories were recoded as either home-based or outside the home as IPV has been associated with women working outside the home in general rather than individual employment categories [69] [70] [71]. At 'partner' level, husband's schooling and employment were entered as categorical indicators and alcohol or drug use as binary. At 'household' level, faith, caste and socioeconomic quintile were entered as categorical indicators. Socioeconomic quintile was entered as a categorical rather than continuous variable to retain categorical consistency and because it was hypothesised that a reduction in odds would be observed in the wealthier quintiles. Quintiles were based on analysis of 22 individual household assets, with scores derived from standardized weights from the first component of a principal components analysis [81] [82].

Statistical analysis

The survey had a single-stage design with clustering and stratification. Data analyses were unweighted because the clusters were of similar size and each was sampled for about 100 questionnaires. Sociodemographic factors were summarised and cross-

For peer review only - http://bmjopen.bmj.com/site/about/guidelines.xhtml

tabulated using frequencies and percentages, and univariable logistic regression models used to test associations between each exposure variable and the outcome. Variables with associations at $p<0.1$ were included in multivariable analysis. Multivariable logistic regression was used to examine associations between outcome variables (disability, IPV, depression, anxiety) and primary exposure variables, adjusting for confounders (individual demographic and socioeconomic characteristics). Post hoc Wald tests were performed to check models. All analyses used survey commands in Stata 15.1 (College Station, TX). The data are openly available online [dataset] [83] Riley, A., Daruwalla, N., Kanougiya, S., Apoorwa, G., Wickenden, M. and Osrin, D. 2021. Data from: Intimate partner violence against women with disability and associated mental health concerns: a cross-sectional survey in Mumbai, India. Dryad Digital Repository, October 18, 2021, https://doi.org/10.5061/dryad.wwpzgmskk.

Ethical considerations

The survey involved disclosing personal and sensitive information, which raises issues of interviewer behaviour, consent, data sharing, and privacy and confidentiality. All

For peer review only

interviewers underwent appropriate training and WHO ethical and safety recommendations were followed [84]. Interview processes were checked through random visits and systematic monitoring by project officers and supervisors.

During the interview, participants were made aware of the potentially sensitive nature of the questions about violence and consent was obtained before they began. Efforts were made to ensure privacy by prearranging visits at a preferred time and location. Participants were able to attend a local community office, but most were interviewed at home. If household members were unwilling to leave, researchers encouraged them to listen to some of the routine questions to be reassured of the survey's nature. Most were amenable to leaving, but when they were not, the interview was terminated and completed over up to three repeat visits

Safeguarding was paramount during the survey. All participants were given information on local counselling and crisis support services for themselves or other women, whether they reported violence or not. Service providers were made aware of the study and the potential for increased service use. Researchers were able to

For peer review only

For peer review only - http://bmjopen.bmj.com/site/about/guidelines.xhtml

arrange referrals if requested. For those who disclosed experience of violence, duty of care was of particular concern. Details of safeguarding measures and counselling services made available to survivors are described elsewhere [56].

Ethics approval

Data collection was approved by the UCL Research Ethics Committee (3546/003 27/09/2017) and the PUKAR (Partners for Urban Knowledge, Action and Research) Institutional Ethics Committee (25/12/2017) [55].

Patient and public involvement

Our research responds to the urgency of preventing violence and improving services for survivors, primarily through community-based programming in informal settlements. It is driven by survivors' needs, one of which is inclusion. We followed a protocol co-developed with community members to achieve maximal inclusion of women with functional difficulties. We discussed the survey questions with participants in a pilot phase and adapted them for subsequent use. Our findings will be shared

For peer review only

through community women's groups, three of which meet monthly in each of the 50 informal settlement clusters.

Results

Interviewers approached 5277 households between 5th December 2017 and 28th March 2019. In 967 (5%) households there was no eligible female resident; in 592 households (11%) the interviewers could not achieve privacy across repeat visits; and in 155 (3%) households a potential participant declined interview. In total, 5122 interviews were conducted.

Disability prevalence

Table 1 summarises the type and frequency of functional difficulties reported by women. 10% had 'some difficulty' in at least one domain, of which the most common were visual (4%) and locomotor (5%). Across all domains, the number of participants reporting a lot of difficulty or no ability at all was low, each less than 1%.

For peer review only - http://bmjopen.bmj.com/site/about/guidelines.xhtml

Table 1: Frequencies of domains of functional difficulty among 5122 women aged 18-49 years living in informal settlements in Mumbai

Functional difficulty	None n (%)	Some difficulty n (%)	A lot of difficulty n (%)	Cannot do at all n (%)
Seeing	4896 (96)	212 (4)	12 (<1)	2 (<1)
Hearing	5078 (99)	40 (<1)	3 (<1)	1 (<1)
Walking	4860 (95)	234 (5)	25 (<1)	3 (<1)
Memory	4988 (97)	123 (2)	11 (<1)	0 (0)
Self-care	5108 (>99)	10 (<1)	4 (<1)	0 (0)
Speaking	5109 (>99)	4 (<1)	9 (<1)	0 (0)
**Washington Group Disability prevalence assessment**				n (%)
At least one domain *some difficulty*				505 (10)
At least one domain *a lot of difficulty*				63 (1)
At least one domain *cannot at all*				6 (<1)
At least one domain *a lot of difficulty* or *cannot at all* or at least *some difficulty* in two domains				178 (3)

For peer review only

Women's sociodemographic profiles and disability

Most women were in their 20s or 30s (80%), currently married (92%) and with one or two children (51%). A fifth of women (19%) reported having no schooling, while 44% had had six to 10 years. Having no remunerated work was common (76%), while those who had an income tended to work from home (15%). In unadjusted analyses, greater

odds of disability were observed in women over 40 (odds ratio 2.7; 95% confidence interval 1.3, 5.5), women who were separated or divorced (3.3; 1.9, 5.6), or widowed (3.6; 2.2, 5.9), and women who had three or more children (1.9; 1.4, 2.4). Attending school for at least six years was associated with lesser odds of disability than no schooling at all (0.8; 0.6, 0.9). Women who earned in the home had greater odds of disability than women who did not (1.5; 1.1, 2.0), as did women from Muslim rather than Hindu families (1.4; 1.1, 1.8).

Associations between disability and past-year IPV

Table 2 presents associations between women's functional difficulty and past-year physical, sexual, and emotional IPV. Of 5122 women, 628 reported suffering physical or sexual IPV in the last year (12%) and 606 had suffered emotional IPV (12%). These proportions were greater for women with disability: 84 of 505 who reported at least some functional difficulty had suffered physical or sexual IPV (17%) and 83 emotional IPV (16%). We had information on socioeconomic asset score for 4918 women who had ever been married, and on their husbands for 4805 women whose husbands were alive. After adjustment for age, marital status, parity, woman's and husband's schooling, woman's and husband's employment, religion, and husband's use of

For peer review only - http://bmjopen.bmj.com/site/about/guidelines.xhtml

For peer review only

alcohol or drugs, these differences were manifest in adjusted odds ratios of 1.68 (95% CI 1.23, 2.29) for physical or sexual and 1.52 (1.16, 2.00) for emotional IPV.

For peer review only

Table 2: Associations between experience of intimate partner violence (physical or sexual IPV, emotional IPV) in the preceding year, disability, and sociodemographic characteristics, for 5122 women aged 18-49 years living in informal settlements in Mumbai

Characteristic (n)	Physical or sexual IPV in last year							Emotional IPV in last year						
	No n (%)	Yes n (%)	OR	(95% CI)	p-value	Adjusted OR	(95% CI)	No n (%)	Yes n (%)	OR	(95% CI)	p-value	Adjusted OR	(95% CI)
Disability														
None	4073 (88)	544 (12)	1			1		4093 (89)	524 (11)	1			1	
At least some difficulty	421 (83)	84 (17)	1.49	1.24, 1.99	0.007	1.68	1.23, 2.29	422 (84)	83 (16)	1.54	1.20, 1.97	0.001	1.52	1.16, 2.00
All	4494 (88)	628 (12)						4515 (88)	607 (12)					
Woman's age (years)														
<20	104 (90)	11 (10)	1			1		108 (94)	7 (6)	1			-	-
20-29	1747 (86)	291 (14)	1.57	0.54, 4.64		0.82	0.26, 2.56	1782 (87)	256 (13)	2.22	0.80, 6.16		-	-
30-39	1808 (88)	248 (12)	1.30	0.43, 3.88		0.57	0.18, 1.86	1812 (88)	243 (12)	2.07	0.74, 5.81		-	-
>40	835 (91)	78 (9)	0.88	0.28, 2.75	<0.001	0.39	0.11, 1.36	812 (89)	101 (11)	1.92	0.69, 5.34	0.329	-	-
All	4494 (88)	628 (12)						4515 (88)	607 (12)					
Marital status														
Married	4112 (87)	593 (13)	1			1		4131 (88)	574 (12)	1			1	
Unmarried	203 (99)	2 (1)	0.07	0.02, 0.27		0.07	0.02, 0.23	204 (99)	1 (<1)	0.04	0.01, 0.26		0.03	0.01, 0.24
Separated or divorced	78 (78)	22 (22)	1.96	1.20, 3.18		1.44	0.88, 2.33	71 (71)	29 (29)	2.94	1.90, 4.54		2.14	1.37, 3.35
Widowed	101 (90)	11 (10)	0.76	0.44, 1.29	0.001	0.79	0.45, 1.41	109 (97)	3 (3)	0.20	0.07, 0.59	<0.001	0.14	0.04, 0.44
All	4494 (88)	628 (12)						4515 (88)	607 (12)					
Parity														
No children	692 (92)	63 (8)	1			1		692 (92)	63 (8)	1			1	
1 or 2	2263 (86)	358 (14)	1.74	1.29, 2.34		1.41	1.04, 1.91	2279 (87)	342 (13)	1.65	1.28, 2.13		1.16	0.89, 1.51
3 or more	1539 (88)	207 (12)	1.48	1.09, 2.00	0.002	1.29	0.92, 1.80	1544 (88)	202 (12)	1.44	1.09, 1.90	0.002	0.96	0.70, 1.31
All	4494 (88)	628 (12)						4515 (88)	607 (12)					

For peer review only

For peer review only - http://bmjopen.bmj.com/site/about/guidelines.xhtml

Woman's schooling														
None	848 (89)	100 (11)	1			1		854 (90)	94 (10)	1			1	
1-5 years	734 (85)	125 (15)	1.44	1.03, 2.02		1.39	0.99, 1.96	737 (86)	122 (14)	1.50	1.13, 2.00		1.45	1.09, 1.94
6-10 years	1976 (87)	304 (13)	1.30	1.00, 1.70		1.24	0.93, 1.66	1983 (87)	297 (13)	1.36	1.07, 1.73		1.29	1.00, 1.67
11 years or more	936 (90)	99 (10)	0.90	0.66, 1.21	0.027	0.93	0.67, 1.29	941 (91)	94 (9)	0.91	0.72, 1.15	0.002	0.95	0.72, 1.24
All	4494 (88)	628 (12)						4515 (88)	607 (12)					
Woman's employment														
None	3437 (89)	433 (11)	1			1		3446 (89)	424 (11)	1			1	
Home-based	665 (86)	108 (14)	1.29	1.07, 1.56		1.22	1.01, 1.49	677 (88)	96 (12)	1.15	0.92, 1.44		1.08	0.85, 1.37
Outside the home	392 (82)	87 (18)	1.76	1.33, 2.32	<0.001	2.11	1.60, 2.80	392 (82)	87 (18)	1.80	1.31, 2.49	0.003	2.00	1.43, 2.80
All	4494 (88)	628 (12)						4515 (88)	607 (12)					

Table 2 continued

Religion														
Hindu	2685 (89)	317 (11)	1			1		2677 (89)	325 (11)	1			1	
Muslim	1629 (86)	273 (14)	1.42	1.15, 1.76		1.38	1.11, 1.72	1655 (87)	247 (13)	1.23	1.00, 1.52		1.23	1.00, 1.54
Buddhist	163 (83)	34 (17)	1.77	1.25, 2.50		1.68	1.16, 2.43	167 (85)	30 (15)	1.48	1.11, 1.97		1.33	1.00, 1.79
Other	17 (81)	4 (19)	1.99	0.64, 6.23	0.003	1.90	0.63, 5.74	16 (76)	5 (24)	2.57	0.90, 7.36	0.013	2.94	0.93, 9.27
All	4494 (88)	628 (12)						4515 (88)	607 (12)					
Caste														
General	2637 (88)	350 (12)	1			-	-	2651 (89)	336 (11)	1			-	-
Other Backward Caste	1064 (87)	157 (13)	1.11	0.92, 1.35		-	-	1063 (87)	158 (13)	1.17	0.95, 1.45		-	-
Scheduled Caste/Tribe	621 (86)	103 (14)	1.25	1.01, 1.54		-	-	629 (87)	95 (13)	1.19	0.93, 1.53		-	-
None stated	172 (91)	18 (9)	0.79	0.45, 1.37	0.202	-	-	172 (91)	18 (9)	0.83	0.47, 1.45	0.375	-	-
All	4494 (88)	628 (12)						4515 (88)	607 (12)					
Socioeconomic quintile (n=4918 ever-married women)														
1 poorest	829 (84)	161 (16)	1			1		849 (86)	141 (14)	1			-	-
2	861 (88)	117 (12)	0.70	0.55, 0.88		0.70	0.54, 0.90	865 (88)	113 (12)	0.79	0.58, 1.07		-	-

For peer review only - http://bmjopen.bmj.com/site/about/guidelines.xhtml

3	865 (88)	118 (12)	0.70	0.52, 0.95		0.72	0.53, 0.99	871 (89)	112 (11)	0.77	0.53, 1.13		-	-
4	876 (89)	108 (11)	0.63	0.49, 0.83		0.66	0.50, 0.88	876 (89)	108 (11)	0.74	0.53, 1.04		-	-
5 least poor	884 (90)	99 (10)	0.58	0.41, 0.81	0.007	0.63	0.45, 0.89	875 (89)	108 (11)	0.74	0.53, 1.04	0.325	-	-
All	4315 (88)	603 (12)						4336 (88)	582 (12)					
Husband's schooling (n=4805 women with living husbands)														
None	391 (81)	90 (19)	1			1		396 (82)	85 (18)	1			1	
1-5 years	507 (86)	83 (14)	0.71	0.51, 1.00		0.73	0.51, 1.04	509 (86)	81 (14)	0.74	0.50, 1.10		0.76	0.51, 1.11
6-10 years	2206 (87)	318 (13)	0.63	0.48, 0.82		0.66	0.50, 0.87	2214 (88)	310 (12)	0.65	0.48, 0.89		0.68	0.50, 0.92
11 years or more	1069 (89)	123 (11)	0.54	0.39, 0.75		0.63	0.41, 0.97	1066 (89)	126 (11)	0.59	0.43, 0.83		0.68	0.49, 0.95
Unknown	17 (91)	1 (9)	0.44	0.29, 0.66	<0.001	0.55	0.37, 0.84	17 (91)	1 (9)	0.48	0.33, 0.70	0.005	0.60	0.40, 0.92
All	4190 (87)	615 (13)						4202 (87)	603 (13)					
Husband employed in previous 12 months (n=4805 women with living husbands)														
No	73 (74)	26 (26)	1			1		76 (77)	23 (23)	1			1	
Yes	4111 (88)	586 (12)	0.40	0.25, 0.64		0.50	0.29, 0.84	4120 (88)	577 (12)	0.46	0.28, 0.76		0.75	0.45, 1.27
Unknown	6 (67)	3 (33)	1.40	0.35, 5.60	0.001	1.20	0.21, 6.91	6 (67)	3 (33)	1.65	0.32, 8.42	0.001	1.34	0.34, 5.27
All	4190 (87)	615 (13)						4202 (87)	603 (13)					
Husband uses alcohol/drugs (n=4805 women with living husbands)														
No	3198 (89)	377 (11)	1			1		3216 (90)	359 (10)	1			1	
Yes	992 (81)	238 (19)	2.04	1.59, 2.61	<0.001	1.81	1.36, 2.40	986 (80)	244 (20)	2.22	1.79, 2.75	<0.001	2.00	1.59, 2.53
All	4190 (87)	615 (13)						4202 (87)	603 (13)					

IPV: intimate partner violence. OR: odds ratio. AOR: adjusted odds ratio. Ref: reference category. ORs and 95% confidence intervals (CI) from univariable and multivariable logistic regression models. Multivariable models (Adjusted OR) include all variables for which Adjusted ORs are presented. Of 5122 women, we had data on socioeconomic status for the 4918 who had ever been married and data on their husbands for 4805 who had not been widowed.

For peer review only

Associations between disability and symptoms of anxiety and depression

Table 3 compares the profiles of women screened as having symptoms of at least moderate anxiety or depression with those who did not. 10% of women fell into this category and disabled women had nearly three times greater odds of doing so than non-disabled women (AOR 2.88, 95% CI 2.17, 3.82). Women who reported physical or sexual IPV (AOR 2.50, CI 1.77, 3.53) or emotional IPV (AOR 2.34, CI 1.59, 3.43) also had greater odds of anxiety or depression. Associations with anxiety or depression were also seen for separated, divorced, or widowed women, who were engaged in remunerated work, and whose husbands used alcohol or drugs. Associations were not seen in unadjusted models for parity or caste.

Table 3: Associations of positive screens for moderate or severe anxiety (GAD-7) or depression (PHQ-9) with disability and intimate partner violence (IPV) in the preceding year, for 5122 women aged 18-49 years living in informal settlements in Mumbai

Characteristic (n)	No, minimal, or mild anxiety or depression n (%)	Moderate or severe anxiety or depression n (%)	OR	(95% CI)	p	Adjusted OR	(95% CI)
Disability							
None	4217 (91)	400 (9)	1			1	
At least some impairment	372 (74)	133 (26)	3.77	2.99, 4.75	<0.001	2.88	2.17, 3.82
All	4589 (90)	533 (10)					
Physical or sexual IPV							

For peer review only

No	4132 (92)	362 (8)	1			1	
Yes	457 (73)	171 (27)	4.27	3.35, 5.45	<0.001	2.50	1.77, 3.53
All	4589 (90)	533 (10)					
Emotional IPV							
No	4150 (92)	365 (8)	1			1	
Yes	439 (72)	168 (28)	4.35	3.37, 5.62	<0.001	2.34	1.59, 3.43
All	4589 (90)	533 (10)					
Woman's age (years)							
<20	102 (89)	13 (11)	1			1	
20-29	1854 (91)	184 (9)	0.78	0.44, 1.38		0.69	0.38, 1.24
30-39	1857 (90)	199 (10)	0.84	0.47, 1.51		0.70	0.38, 1.28
>40	776 (85)	137 (15)	1.39	0.75, 2.56	<0.001	0.95	0.50, 1.82
All	4589 (90)	533 (10)					
Marital status							
Married	4267 (91)	438 (9)	1			1	
Unmarried	189 (92)	16 (8)	0.82	0.46, 1.48		1.21	0.64, 2.25
Separated or divorced	57 (57)	43 (43)	7.35	4.72, 11.45		4.73	2.68, 9.34
Widowed	76 (68)	36 (32)	4.61	2.91, 7.33	<0.001	3.25	1.72, 6.12
All	4589 (90)	533 (10)					
Woman's schooling							
None	819 (86)	129 (14)	1 =			1	
1-5 years	758 (88)	101 (12)	0.85	0.61, 1.17		0.72	0.50, 1.06
6-10 years	2046 (90)	234 (10)	0.73	0.56, 0.94		0.70	0.50, 0.96
11 years or more	966 (93)	69 (7)	0.45	0.32, 0.65	<0.001	0.59	0.39, 0.90
All	4589 (90)	533 (10)					
Woman's employment							
None	3535 (91)	335 (9)	1			1	
Home-based	662 (86)	111 (14)	1.77	1.39, 2.25		1.48	1.12, 1.96
Outside the home	392 (82)	87 (18)	2.34	1.73, 3.18	<0.001	1.70	1.19, 2.44
All	4589 (90)	533 (10)					
Religion							
Hindu	2737 (91)	265 (9)	1			1	
Muslim	1669 (88)	233 (12)	1.44	1.15, 1.81		1.28	0.98, 1.67
Buddhist	165 (84)	32 (16)	2.00	1.27, 3.17		1.63	0.98, 2.70
Other	18 (86)	3 (14)	1.72	0.45, 6.61	0.003	1.13	0.40, 3.22
All	4589 (90)	533 (10)					
Socioeconomic quintile (n=4918 ever-married women)							
1 poorest	857 (87)	133 (13)	1			1	
2	877 (90)	101 (10)	0.74	0.56, 0.98		0.73	0.53, 1.02
3	879 (89)	104 (11)	0.76	0.56, 1.04		0.77	0.54, 1.11
4	891 (91)	93 (9)	0.67	0.50, 0.90		0.68	0.48, 0.96
5 least poor	903 (92)	80 (8)	0.57	0.41, 0.80	0.014	0.64	0.43, 0.96
All	4407 (90)	511 (10)					
Husband's schooling (n=4805 women with living husbands)							

For peer review only - http://bmjopen.bmj.com/site/about/guidelines.xhtml

None	410 (85)	71 (15)	1			1	
1-5 years	517 (88)	73 (12)	0.82	0.59, 1.13		1.04	0.76, 1.44
6-10 years	2282 (90)	242 (10)	0.61	0.44, 0.84		1.01	0.73, 1.38
11 years or more	1103 (91)	89 (9)	0.58	0.38, 0.91		1.19	0.72, 1.95
Unknown	12 (94)	6 (6)	0.37	0.24, 0.58	<0.001	0.84	0.47, 1.50
All	4324 (90)	481 (10)					
Husband employed in previous 12 months (n=4805 women with living husbands)							
No	72 (73)	27 (27)	1			1	
Yes	4247 (90)	450 (10)	0.28	0.19, 0.42		0.81	0.45, 1.48
Unknown	5 (56)	4 (44)	2.13	0.49, 9.23	<0.001	1.70	0.29, 9.94
All	4324 (90)	481 (10)					
Husband uses alcohol or drugs (n=4805 women with living husbands)							
No	3317 (93)	258 (7)	1			1	
Yes	1007 (82)	223 (18)	2.85	2.19, 3.70	<0.001	1.98	1.48, 2.64
All	4324 (90)	481 (10)					

OR: odds ratio. AOR: adjusted odds ratio. ORs and 95% confidence intervals (CI) from univariable and multivariable logistic regression models.

Multivariable models (Adjusted OR) include all variables for which Adjusted ORs are presented. Of 5122 women, we had data on socioeconomic status for the 4918 who had ever been married, and data on their husbands for 4805 who had not been widowed.

Table 4 examines associations in more detail, presenting odds ratios for positive screens for moderate or severe anxiety, depression, and suicidal thinking in the presence of disability and physical, sexual, or emotional IPV. Overall, 6% of women screened positive for anxiety symptoms and 9% for symptoms of depression, while 13% had experienced suicidal thinking. In unadjusted models, disability increased the odds of each of these by two to four times. Adjusting for potential confounders reduced the odds ratios, but they remained substantial. IPV may also be a confounding factor, as it is associated with both mental health concerns and disability. Adjusting for IPV

For peer review only - http://bmjopen.bmj.com/site/about/guidelines.xhtml
For peer review only

and sociodemographic characteristics further reduced the odds ratios, but women with disability continued to have elevated odds of having a mental health concern. In this second adjusted model, women with functional difficulty had more than twice the odds of reporting anxiety symptoms as non-disabled women (AOR2 2.50, CI 1.78, 3.49) and nearly three times the odds of depression (AOR2 2.91, CI 2.13, 3.99). They had almost twice the odds of reporting suicidal thinking (AOR2 1.94, CI 1.50, 2.50). Independent of functional status, physical, sexual and emotional IPV were all associated with greater odds of having a mental health concern.

Table 4: Unadjusted and adjusted logistic regression models for associations between disability status, symptoms of anxiety and depression, and suicidal thinking, for 5122 women aged 18-49 years living in informal settlements in Mumbai

	Moderate or severe anxiety n (%)		OR	(95% CI)	Adjusted OR 1	(95% CI)	Adjusted OR 2	(95% CI)
	No	Yes						
Disability status								
None	4389 (95)	228 (5)	1		1		1	
At least some difficulty	425 (84)	80 (16)	3.62	2.77, 4.73	2.75	2.01, 3.76	2.50	1.78, 3.49
All	4814 (94)	308 (6)						
Physical or sexual IPV								
No	4293 (95)	201 (4)	1		1			
Yes	521 (83)	107 (17)	4.39	3.41, 5.65	4.05	2.98, 5.50	-	-
All	4814 (94)	308 (6)						
Emotional IPV								
No	4315 (96)	200 (4)	1		1			
Yes	499 (82)	108 (18)	4.67	3.59, 6.07	4.11	3.08, 5.49	-	-
All	4814 (94)	308 (6)						

For peer review only - http://bmjopen.bmj.com/site/about/guidelines.xhtml

	Moderate or severe depression n (%) No	Yes	OR	(95% CI)	Adjusted OR 1	(95% CI)	Adjusted OR 2	(95% CI)
Disability status								
None	4280 (93)	337 (7)	1		1		1	
At least some difficulty	383 (76)	122 (24)	4.05	3.16, 5.18	3.12	2.36, 4.14	2.91	2.13, 3.99
All	4663 (91)	459 (9)						
Physical or sexual IPV								
No	4186 (93)	308 (7)	1		1			
Yes	477 (76)	151 (24)	4.30	3.40, 5.44	3.97	3.10, 5.09	-	-
All	4663 (91)	459 (9)						
Emotional IPV								
No	4204 (92)	311 (7)	1		1			
Yes	459 (76)	148 (24)	4.36	3.40, 5.58	4.01	3.04, 5.29	-	-
All	4663 (91)	459 (9)						
	**Suicidal ideation n (%) No**	**Yes**	**OR**	**(95% CI)**	**Adjusted OR 1**	**(95% CI)**	**Adjusted OR 2**	**(95% CI)**
Disability status								
None	4089 (89)	528 (11)	1		1		1	
At least some difficulty	384 (76)	121 (24)	2.44	1.98, 3.01	2.13	1.66, 2.75	1.94	1.50, 2.50
All	4473 (87)	649 (13)						
Physical or sexual IPV								
No	4087 (91)	407 (9)	1		1			
Yes	386 (61)	242 (39)	6.30	5.01, 7.92	6.04	4.80, 7.62	-	-
All	4473 (87)	649 (13)						
Emotional IPV								
No	4112 (91)	403 (9)	1		1			
Yes	361 (59)	246 (41)	6.95	5.81, 8.33	6.55	5.36, 8.00	-	-
All	4473 (87)	649 (13)						

OR: odds ratio. AOR: adjusted odds ratio. ORs and 95% confidence intervals (CI) from univariable and multivariable logistic regression models.

Adjusted OR 1: adjusted for sociodemographic covariates: woman's age, marital status, schooling, employment, religion, socioeconomic quintile, and husband's schooling, employment, and alcohol or drug use.

Adjusted OR 2: adjusted for sociodemographic covariates and preceding year physical or sexual IPV and emotional IPV.

Discussion

For peer review only - http://bmjopen.bmj.com/site/about/guidelines.xhtml

In a cross-sectional survey of women living in informal settlements in Mumbai, disability was more common in women over 30 years of age and with children, who were separated, divorced or widowed, and from poorer households. Women with disability had over 50% greater odds of reporting physical, sexual, or emotional IPV than non-disabled women, and disabled women and those who had experienced IPV had greater odds of anxiety, depression, and suicidal thinking. Overall, the increased odds of disabled women experiencing IPV are consistent with studies from high-income [12] [85] and low-income settings [37] [86]. These findings contribute to our understanding of a serious but insufficiently investigated public health issue [15].

The reported prevalence of IPV among all women (12% for physical or sexual and emotional IPV) was lower than at national level [28] [29] and in slums [32] [33]. It was also lower among disabled women (17% for physical or sexual IPV, 16% for emotional IPV) compared with other studies [11, 35, 36]. The cause of this disparity is unclear. Strong efforts were made to help participants feel able to respond openly to questions, and the study used definitions of violence and survey instruments comparable with other studies: the lower rates may therefore be a fair reflection of levels of violence in

For peer review only - http://bmjopen.bmj.com/site/about/guidelines.xhtml

this community. Indeed, the rate accords closely with a study of physical (12%), sexual (2%), and emotional IPV (8%) experienced six weeks postpartum also undertaken in Mumbai informal settlements [74]. It is possible, nevertheless, that lower rates of violence may reflect under-reporting. Multiple factors play a role in the reporting of violence, such as a community's redressal structures, the extent of local welfare activities, and social norms. It may be that some communities are particularly close and not forthcoming enough to report violence. The context of each community is different and efforts must be made to understand local norms and behaviours in order to contextualise research outcomes.

The mental health burden in India is high and greater treatment provision is needed [87] [88]. One-in-seven Indian people are believed to be affected by some mental disorder and the burden of depressive and anxiety disorders is believed to be greatest among women [89]. For women with disabilities, the burden may be higher [90]. Two studies from South India found that disabled women were more likely to experience comorbid depression and diabetes than non-disabled women [91] [92]. One of these found that disabled women were nearly ten times more likely to have depression than

For peer review only - http://bmjopen.bmj.com/site/about/guidelines.xhtml

non-disabled women (AOR 9.5, CI, 2.2-40.8) [91]. Our study supports these findings: disabled women had more mental health concerns even when controlling for socioeconomic conditions and experience of violence. Whether poor mental health and disability precede violence or vice-versa cannot be determined here. Comorbid mental health disorders may also result from pre-existing health conditions and be exacerbated by exposure to violence. These issues should be the subject of future research. Further, as with the elevated vulnerability to violence, this burden of mental disorder needs to be communicated to relevant stakeholders and methods to address it developed.

The intersection of poverty, inequality, and discriminatory social norms is central to both violence and mental health disorder [93] [94]. In visualising and categorising interconnected determinants, the ecological framework used in our analysis has utility. Determining the source of violence, whether at individual micro or broader macro levels, is less clear-cut. The effects of multidimensional poverty and gender in ecologies of violence have received attention in recent years [9] [95], but more research is needed, both quantitative and qualitative, to understand the place of

For peer review only - http://bmjopen.bmj.com/site/about/guidelines.xhtml

disability and mental health in this matrix, particularly in the context of India. A potential data source is India's National Family Health Survey (NFHS), which has been broadened to include measures of disability [96]. The NFHS has already been used widely in IPV studies involving non-disabled women [97] [40]. It is hoped that this change will bring greater visibility to women with disabilities in investigations of violence.

Alongside improved visibility, regressive social and cultural norms must be challenged. These include the conception of disability as a personal failing or a sign of ill fate, and of disabled people as deserving of pity [98] [99], as well as patriarchal and stigmatizing attitudes towards women and mental disorder in general [100, 101], and tolerance of violence among both women and men [102]. This can be achieved, in part, through integrated inclusive interventions that incorporate anti-violence messages with other approaches, and through targeted interventions that seek to change violence-related norms directly [103] [55]. These efforts work best when addressing both men and women across all social classes and levels of the social ecology [65]. A recent targeted pilot study in Jharkhand, India that mobilised community resources through

For peer review only

participatory learning and action groups facilitated by Accredited Social Health Activists (ASHAs) reported reductions in experience and tolerance of violence and greater levels of help-seeking [104]. The study contributes to a growing body of evidence demonstrating that norms and practices that perpetuate inequities and violence can be challenged by community mobilisation and education [105]. Such interventions should work with disabled communities and activists, incorporating disability-positive messaging, to challenge discrimination at the community level and provide a more nuanced and inclusive approach to anti-violence efforts.

In healthcare settings, efforts should be made to identify and support disabled women unknown to healthcare and violence support services. Routine healthcare and IPV education and screening programmes need to be disability-inclusive and the vulnerability of disabled women needs to be incorporated into service provision. At screenings, disabled women should have access to safe and private spaces, away from accompanying family members who may be party to violence. Those delivering screening may need training to consider different kinds of functional difficulty and access needs such as adapted communication. Training should also incorporate

For peer review only - http://bmjopen.bmj.com/site/about/guidelines.xhtml

issues of trust and respect in order to challenge the stereotyping that can cause women with disabilities to be disbelieved when reporting episodes of violence [99] [106]. The need to challenge stereotypes and promote respect extends to intersecting issues such as widowhood, separation and divorce. Women can be stigmatised in India after the loss of a husband or the ending of a marriage, with a burden of fault placed on them [107]. In our study and elsewhere [10], women with disabilities were more likely to be separated or divorced, which is likely to further compound any stigma they already experience. Healthcare workers are well placed to challenge these kinds of stereotypes, but may themselves be party to sustaining them and require support to break reductive patterns of behaviour and thinking.

At a policy level, three major instruments in Indian law pertain directly to the evidence presented herein: the Protection of Women from Domestic Violence Act, 2005 (PWDVA) [108], the Rights of Persons with Disability Act, 2016 [109], and the Mental Healthcare Act, 2017 [110]. The greater scope for understanding and protection that these most recent instruments afford is a step in the right direction. The Rights of Persons with Disability Act brought India's disability policy in line with the United

For peer review only - http://bmjopen.bmj.com/site/about/guidelines.xhtml

Nations Convention on the Rights of Persons with Disabilities (UN-CRPD) and introduced a rights-based approach towards disability [111], while the Mental Healthcare Act shifted the approach to mental health away from criminalisation towards provision of healthcare [112]. However, there were limitations in the development of both [111] [113], including the Mental Healthcare Act's failure to sufficiently address stigma [114]. As for the PWDVA, India's persistently high rates of domestic violence, which have grown during the COVID-19 pandemic [115], are testament to the act's failure to effect any real change in society [116] [117]. This further demonstrates that, while policy can be an important tool for addressing attitudinal and environmental barriers, change will remain elusive without commitment to implementation supported by adequate funding and monitoring-accountability mechanisms.

Limitations

Limitations include the cross-sectional study design, which precludes determination of whether disability was an outcome of or risk factor for IPV. Use of self-report for exposure items is a further limitation, although the recall period was short, participants

For peer review only - http://bmjopen.bmj.com/site/about/guidelines.xhtml

being asked about their experiences in the last year. Responses to questions about IPV and mental health may have been self-censored due to the sensitive nature of the questions and therefore underestimated, despite researchers being familiar figures in the community and their efforts to develop trust. Use of self-report for disability is also a limitation and may be reflected in the low rates. The WG-SS determines a person's limitations based on self-perception in relation to their environment. Given the disadvantaged setting in which the participants lived, this may have also impacted the disability rates.

Conclusion

Violence against women in India is a major and ongoing public health problem. Women with disabilities are perhaps the most marginalised of all and experience violence to a greater degree than others. This violence derives from a culture that tolerates and perpetuates the marginalisation of both women in general and women with disabilities in particular and contributes to an unacceptable physical and mental health burden. The issue requires greater attention from national and community

For peer review only

leaders to address its causes, especially poverty and gender inequality, and to meet India's commitments to its disabled population.

Acknowledgments

We are grateful to the women and community guardians who agreed to contribute to the study. We thank the investigators, data collection supervisors Miheeka Vast and Manju Singh, Bhaskar Kakad for supervision, Gauri Savkur for training support, Unnati Machchhar and Shilpa Adelkar for subsequent supervision of the intervention programme, Archana Bagra and Vibhavari Bali for financial and human resources management, and Vanessa D'Souza and Shanti Pantvaidya for leadership at SNEHA.

For peer review only

Contributorship

AR, ND and DO conceived the study. ND, MW, SK, AG, and DO developed the methodology for data collection. ND, SK, and AG oversaw investigation. AR and DO curated the data and designed the analysis. AR did the analysis and wrote the first draft. ND managed the project and ND and DO acquired funding. All authors critically reviewed drafts of the manuscript and read and commented on the final version.

Competing interests.

The authors declare no competing interest.

Funding

This research was funded in part by the Wellcome Trust (206417). For the purpose of open access, the author has applied a CC BY public copyright licence to any Author Accepted Manuscript version arising from this submission. The funder had no role in study design, data collection and analysis, decision to publish, or preparation of the manuscript.

Data sharing

Data are available in Open Science Framework: Osrin D. Disability dataset. 2021.osf.io/th6a3

For peer review only

References

1. WHO. 2011. *World report on disability*. https://www.who.int/disabilities/world_report/2011/report/en/.
2. UN. 2006. *Convention on the Rights of Persons with Disabilities* (13 December 2006); A/RES/61/106, Annex I. https://www.un.org/development/desa/disabilities/convention-on-the-rights-of-persons-with-disabilities.html.
3. UNESCO. 2018. *Education and Disability: Analysis of Data from 49 Countries. Information Paper No. 49*. Institute for Statistics. http://uis.unesco.org/en/news/education-and-disability-analysis-data-49-countries.
4. Mitra, S., Posarac, A. and Vick, B. 2013. Disability and poverty in developing countries: a multidimensional study. *World Development*. 41; pp.1-18.
5. Heymann, J., Stein, M.A. and Moreno, G. 2013. *Disability and Equity at Work*. Oxford University Press, Oxford.
6. Mithen, J., Aitken, Z., Ziersch, A. and Kavanagh, A.M. 2015. Inequalities in social capital and health between people with and without disabilities. *Social Science & Medicine*. 126; pp.26-35.
7. Banks, L.M., Kuper, H. and Polack, S. 2017. Poverty and disability in low-and middle-income countries: A systematic review. *PloS One*. 12(12), p.e0189996.

For peer review only - http://bmjopen.bmj.com/site/about/guidelines.xhtml

8. Braithwaite, J., Mont, D. 2009. Disability and poverty: a survey of World Bank poverty assessments and implications. *Alter*. 3(3); pp.219-232.
9. Groce, N., Kett, M., Lang, R. and Trani, J.F. 2011. Disability and poverty: The need for a more nuanced understanding of implications for development policy and practice. *Third World Quarterly*. 32(8); pp.1493-1513.
10. Daruwalla, N., Chakravarty, S., Chatterji, S., Shah More, N., Alcock, G., Hawkes, S. and Osrin, D. 2013. Violence against women with disability in Mumbai, India: a qualitative study. *Sage Open*. 3(3); p.2158244013499144.
11. Rashid, S., Daruwalla, N., Puri, M., Hawkes, S. and Chow, J. 2012. *Count me in! Research Report: Violence Against Disabled, Lesbian, and Sex-Working Women in Bangladesh, India, and Nepal*. CREA. http://www.creaworld.org/sites/default/files/The%20Count%20Me%20In!%20Research%20Report.pdf.
12. Brownridge, D.A. 2006. Partner violence against women with disabilities: Prevalence, risk and explanations. *Violence Against Women*. 12; pp.805-822.
13. Krug, E.G., Mercy, J.A., Dahlberg, L.L. and Zwi, A.B. (eds.). 2002. *World Report on Violence and Health*. World Health Organisation. https://www.who.int/violence_injury_prevention/violence/world_report/en/.
14. Butchart, A., Mikton, C. 2014. Global Status Report on Violence Prevention. *World Health Organisation*. https://www.who.int/violence_injury_prevention/violence/status_report/2014/en/.
15. Hughes, K., Bellis, M.A., Jones, L., Wood, S., Bates, G., Eckley, L., McCoy, E., Mikton, C., Shakespeare, T. and Officer, A. 2012. Prevalence and risk of violence against adults with disabilities: a systematic review and meta-analysis of observational studies. *The Lancet*. 379(9826); pp.1621-1629.
16. García-Moreno, C., Pallitto, C., Devries, K., Stöckl, H., Watts, C. and Abrahams, N. 2013. Global and regional estimates of violence against women: prevalence and health effects of intimate partner violence and non-partner sexual violence. *World Health Organization*. https://www.who.int/reproductivehealth/publications/violence/9789241564625/en/.
17. Cieza, A., Sabariego, C., Bickenbach, J. and Chatterji, S. 2018. Rethinking disability. *BMC Medicine*. 16(1); pp.1-5.
18. Mikton, C., Maguire, H. and Shakespeare, T. 2014. A systematic review of the effectiveness of interventions to prevent and respond to violence against persons with disabilities. *Journal of Interpersonal Violence*. 29(17); pp.3207-3226.
19. Government of India. 2016a. *Disabled Persons in India: A statistical profile 2016*. Ministry of Statistics and Programme Implementation. http://mospi.nic.in/sites/default/files/publication_reports/Disabled_persons_in_India_2016.pdf.
20. Government of India. 2018. *Persons with Disabilities in India*. National Statistics Office. http://www.mospi.gov.in/sites/default/files/publication_reports/Report_583_Final_0.pdf.
21. Dandona, R., Pandey, A., George, S., Kumar, G.A. and Dandona, L. 2019. India's disability estimates: Limitations and way forward. *PloS One*. 14(9).
22. O'Keefe, P. 2009. *People with Disabilities in India: From Commitments to Outcomes*. World Bank Human Development Unit, South Asia Region. https://documents.worldbank.org/en/publication/documents-

For peer review only - http://bmjopen.bmj.com/site/about/guidelines.xhtml

reports/documentdetail/577801468259486686/people-with-disabilities-in-india-from-commitments-to-outcomes.

23. Mitra, S. and Sambamoorthi, U. 2014. Disability prevalence among adults: estimates for 54 countries and progress toward a global estimate. *Disability and Rehabilitation*. 36(11); pp.940-947.
24. Velayutham, B., Kangusamy, B. and Mehendale, S. 2017. Prevalence of Disability in Tamil Nadu, India. *The National Medical Journal of India*. 30(3); p.125.
25. Mopari, R., Garg, B., Puliyel, J. and Varughese, S. 2019. Measuring disability in an urban slum community in India using the Washington Group questionnaire. *Disability and Health Journal*. 12(2); pp.263-268.
26. Gudlavalleti, M.V.S. 2014. *The Telangana Disability Study, India Country Report*. International Centre for Evidence in Disability and London School of Hygiene and Tropical Medicine. https://www.researchgate.net/publication/280312727_Telangana_Disability_Study.
27. Saikia, N., Bora, J.K., Jasilionis, D. and Shkolnikov, V.M. 2016. Disability divides in India: evidence from the 2011 census. *PloS One*. 11(8).
28. IIPS. 2017. *National Family Health Survey 2015-16 (NFHS-4)*. International Institute for Population Sciences, Government of India, Mumbai. https://dhsprogram.com/pubs/pdf/FR339/FR339.pdf.
29. Kalokhe, A., del Rio, C., Dunkle, K., Stephenson, R., Metheny, N., Paranjape, A. and Sahay, S. 2017. Domestic violence against women in India: A systematic review of a decade of quantitative studies. *Global Public Health*. 12(4); pp.498-513.
30. Begum, S., Donta, B., Nair, S. and Prakasam, C.P. 2015. Socio-demographic factors associated with domestic violence in urban slums, Mumbai, Maharashtra, India. *The Indian Journal of Medical Research*. 141(6); p.783.
31. Dasgupta, A., Preeti, P.S., Sahoo, S.K., Biswas, D., Kumar, A. and Das, M. 2015. Domestic Violence and its Determinants: A cross-sectional study among women in a slum of Kolkata. *Indian Journal of Community Health*. 27(3); pp.334-340.
32. Gaikwad, V., Rao, D.H. 2014. A Cross-sectional Study of Domestic Violence Perpetrated by Intimate Partner against Married Women in the Reproductive Age Group in an Urban Slum Area in Mumbai. *Indian Journal of Public Health Research & Development*. 5(1); pp.49-54.
33. Pal, J., Mullick, T.H., Ahmad, S. and Yadav, A.K. 2017. Domestic Violence against Women-An Unsolved Issue: A Community Based Study in an Urban Slum of Kolkata, India. *Journal of Clinical & Diagnostic Research*. 11(10).
34. Sinha, A., Mallik, S., Sanyal, D., Dasgupta, S., Pal, D. and Mukherjee, A. 2012. Domestic violence among ever married women of reproductive age group in a slum area of Kolkata. *Indian Journal of Public Health Research & Development*. 56(1); p.31.
35. Deepak, S., Kumar, J., Santosh, B., Gornalli, S., Manikappa, P., Vyjantha, U. and Giriyappa, R. 2014. Violence against persons with disabilities in Bidar District, India. *Disability, CBR & Inclusive Development*. 25(2); pp.35-53.
36. Mohapatra, S. and Mohanty, M. 2005. *Abuse and activity limitation - A study on domestic violence against disabled women in Orissa, India*. Oxfam, India.
37. Gupta, J., Cardoso, L.F., Ferguson, G., Shrestha, B., Shrestha, P.N., Harris, C., Groce, N. and Clark, C.J. 2018. Disability status, intimate partner violence and perceived social support among married women in three districts of the Terai region of Nepal. *BMJ Global Health*. 3(5); p.e000934.

For peer review only

38. Hasan, T., Muhaddes, T., Camellia, S., Selim, N. and Rashid, S.F. 2014. Prevalence and experiences of intimate partner violence against women with disabilities in Bangladesh: Results of an explanatory sequential mixed-method study. *Journal of Interpersonal Violence*. 29(17); pp.3105-3126.
39. Puri, M., Misra, G. and Hawkes, S. 2015. Hidden voices: prevalence and risk factors for violence against women with disabilities in Nepal. *BMC Public Health*. 15(1); p.261.
40. Sabri, B., Renner, L.M., Stockman, J.K., Mittal, M. and Decker, M.R. 2014. Risk factors for severe intimate partner violence and violence-related injuries among women in India. *Women & Health*. 54(4); pp.281-300.
41. Spiwak, R., Logsetty, S., Afifi, T.O. and Sareen, J. 2015. Severe partner perpetrated burn: Examining a nationally representative sample of women in India. *Burns*. 41(8); pp.1847-1854.
42. Silverman, J.G., Decker, M.R., Saggurti, N., Balaiah, D. and Raj, A. 2008. Intimate partner violence and HIV infection among married Indian women. *JAMA*. 300(6); pp.703-710.
43. Sudha, S. and Morrison, S. 2011. Marital violence and women's reproductive health care in Uttar Pradesh, India. *Women's Health Issues*. 21(3); pp.214-221.
44. Anand, E., Unisa, S. and Singh, J. 2017. Intimate partner violence and unintended pregnancy among adolescent and young adult married women in south Asia. *Journal of Biosocial Science*. 49(2); pp.206-21.
45. Dhar, D., McDougal, L., Hay, K., Atmavilas, Y., Silverman, J., Triplett, D. and Raj, A. 2018. Associations between intimate partner violence and reproductive and maternal health outcomes in Bihar, India: a cross-sectional study. *Reproductive Health*. 15(1); p.109.
46. Jejeebhoy, S.J., Santhya, K.G. and Acharya, R. 2013. Physical and sexual violence and symptoms of gynaecological morbidity among married young women in India. *Global Public Health*. 8(10); pp.1151-1167.
47. Chandra, P.S., Satyanarayana, V.A. and Carey, M.P. 2009. Women reporting intimate partner violence in India: Associations with PTSD and depressive symptoms. *Archives of Women's Mental Health*. 12(4); p.203.
48. Chowdhury, A.N., Brahma, A., Banerjee, S. and Biswas, M.K. 2009. Pattern of domestic violence amongst non-fatal deliberate self-harm attempters: A study from primary care of West Bengal. *Indian Journal of Psychiatry*. 51(2); p.96.
49. Maselko, J. and Patel, V. 2008. Why women attempt suicide: the role of mental illness and social disadvantage in a community cohort study in India. *Journal of Epidemiology & Community Health*. 62(9); pp.817-822.
50. Suryanarayana, M., Agrawal, A. and Prabhu, K.S. 2011. *Inequality adjusted human development index for India's states*. United Nations Development Programme, India.
51. MCGM. 2020. *Mumbai Census Population*. Municipal Corporation of Greater Mumbai. https://portal.mcgm.gov.in/irj/portal/anonymous/qlvitalstatsreport?guest_user=english.
52. Chandramouli, C. 2011. *Housing stock, amenities and assets in slums - Census 2011*. New Delhi: Office of the Registrar General and Census Commissioner.
53. Ezeh, A., Oyebode, O., Satterthwaite, D., Chen, Y.F., Ndugwa, R., Sartori, J., Mberu, B., Melendez-Torres, G.J., Haregu, T., Watson, S.I. and Caiaffa, W. 2017. The history, geography, and sociology of slums and the health problems of people who live in slums. *The Lancet*. 389(10068); pp.547-558.

For peer review only - http://bmjopen.bmj.com/site/about/guidelines.xhtml

54. UN-Habitat. 2017. *Human Rights in Cities Handbook Series Volume 1: The Human Rights-Based Approach to Housing and Slum Upgrading*. https://www.ohchr.org/Documents/Issues/Housing/InformalSettlements/UNHABITAT_HumanRights-BasedApproch.pdf.
55. Daruwalla, N., Machchhar, U., Pantvaidya, S., D'Souza, V., Gram, L., Copas, A. and Osrin, D. 2019. Community interventions to prevent violence against women and girls in informal settlements in Mumbai: the SNEHA-TARA pragmatic cluster randomised controlled trial. *Trials*. 20(1); p.743.
56. Daruwalla, N., Kanougiya, S., Gupta, A., Gram, L. and Osrin, D. . 2020. Prevalence of domestic violence against women in informal settlements in Mumbai, India: a cross-sectional survey. *BMJ Open*. 10:e042444. DOI: 10.1136/bmjopen-2020-042444.
57. Kalokhe, A.S., Stephenson, R., Kelley, M.E., Dunkle, K.L., Paranjape, A., Solas, V., Karve, L., del Rio, C. and Sahay, S. 2016. The development and validation of the Indian family violence and control scale. *PLoS One*. 11(1); p.e0148120.
58. Johnson, H., Ollus, N. and Nevala, S. 2008. *Violence Against Women: An International Perspective*. Springer Science & Business Media, New York.
59. García-Moreno, C., Jansen, H.A., Ellsberg, M., Heise, L. and Watts, C. 2005. WHO multi-country study on women's health and domestic violence against women. *World Health Organization*. https://www.who.int/reproductivehealth/publications/violence/24159358X/en/.
60. Washington Group on Disability Statistics. 2009. *Understanding and interpreting disability as measured using the WG short set of questions*. https://www.cdc.gov/nchs/data/washington_group/meeting8/interpreting_disability.pdf.
61. Spitzer, R.L., Kroenke, K., Williams, J.B. and Löwe, B. 2006. A brief measure for assessing generalized anxiety disorder: the GAD-7. *Archives of Internal Medicine*. 166(10); pp.1092-1097.
62. Kroenke, K., Spitzer, R.L. and Williams, J.B. 2001. The PHQ-9: validity of a brief depression severity measure. *Journal of General Internal Medicine*. 16(9); pp.606-613.
63. Kroenke, K. and Spitzer, R.L. 2010. *Instruction manual: Instructions for patient health questionnaire (PHQ) and GAD-7 measures*. https://www.phqscreeners.com/.
64. Heise, L. 1998. Violence against women: An integrated, ecological framework. *Violence Against Women*. 4(3); pp.262-290.
65. Heise, L. 2011. *What works to prevent partner violence? An evidence overview*. STRIVE. http://strive.lshtm.ac.uk/system/files/attachments/What%20works%20to%20prevent%20partner%20violence.pdf.
66. Jain, S., Varshney, K., Vaid, N.B., Guleria, K., Vaid, K. and Sharma, N. 2017. A hospital-based study of intimate partner violence during pregnancy. *International Journal of Gynecology & Obstetrics*. 137(1); pp.8-13.
67. Mahapatro, M., Gupta, R.N. and Gupta, V. 2012. The risk factor of domestic violence in India. *Indian journal of Community Medicine: Official Publication of Indian Association of Preventive & Social Medicine*. 37(3); p.153.
68. Shrivastava, P.S. and Shrivastava, S.R. 2013. A study of spousal domestic violence in an urban slum of Mumbai. *International Journal of Preventive Medicine*. 4(1); p.27.
69. Biswas, C.S. 2017. Spousal Violence against Working Women in India. *Journal of Family Violence*. 32(1); pp.55-67.
70. Bhattacharya, H. 2015. Spousal violence and Women's employment in India. *Feminist Economics*. 21(2); pp.30-52.

For peer review only - http://bmjopen.bmj.com/site/about/guidelines.xhtml

71. Weitzman, A. 2014. Women's and men's relative status and intimate partner violence in India. *Population and Development Review*. 40(1); pp.55-75.
72. Rapp, D., Zoch, B., Khan, M.M.H., Pollmann, T. and Krämer, A. 2012. Association between gap in spousal education and domestic violence in India and Bangladesh. *BMC Public Health*. 12(1); p.467.
73. Kimuna, S.R., Djamba, Y.K., Ciciurkaite, G. and Cherukuri, S. 2013. Domestic violence in India: Insights from the 2005-2006 national family health survey. *Journal of Interpersonal Violence*. 28(4); pp.773-807.
74. Das, S., Bapat, U., More, N.S., Alcock, G., Joshi, W., Pantvaidya, S. and Osrin, D. 2013. Intimate partner violence against women during and after pregnancy: a cross-sectional study in Mumbai slums. *BMC Public Health*. 13(1); p.817.
75. Wagman, J.A., Donta, B., Ritter, J., Naik, D.D., Nair, S., Saggurti, N., Raj, A. and Silverman, J.G. 2018. Husband's alcohol use, intimate partner violence, and family maltreatment of low-income postpartum women in Mumbai, India. *Journal of Interpersonal Violence*. 33(14); pp.2241-2267.
76. Huria, K.A., Deepti, D., Lajya, D. and Sunder, S.S. 2005. Domestic violence in pregnancy in North Indian women. *Indian Journal of Medical Sciences*. 59(5); pp.195-199.
77. Chibber, K.S., Krupp, K., Padian, N. and Madhivanan, P. 2012. Examining the determinants of sexual violence among young, married women in Southern India. *Journal of Interpersonal Violence*. 27(12); pp.2465-2483.
78. Dalal, K., Lindqvist, K. 2012. A national study of the prevalence and correlates of domestic violence among women in India. *Asia Pacific Journal of Public Health*. 24(2); pp.265-277.
79. Krishnan, S. 2005. Gender, caste, and economic inequalities and marital violence in rural South India. *Health Care for Women International*. 26(1); pp.87-99.
80. Victora, C.G., Huttly, S.R., Fuchs, S.C. and Olinto, M.T. 1997. The role of conceptual frameworks in epidemiological analysis: a hierarchical approach. *International Journal of Epidemiology*. 26(1); pp.224-227.
81. Filmer, D., Pritchett, L.H. 2001. Estimating wealth effects without expenditure data—or tears: an application to educational enrollments in states of India. *Demography*. 38(1); pp.115-132.
82. Vyas, S. and Kumaranayake, L. 2006. Constructing socio-economic status indices: how to use principal components analysis. *Health Policy and Planning*. 21(6); pp.459-468.
83. Riley, A., Daruwalla, N., Kanougiya, S., Apoorwa, G., Wickenden, M. and Osrin, D. 2021. Data from: Intimate partner violence against women with disability and associated mental health concerns: a cross-sectional survey in Mumbai, India. *Dryad Digital Repository*. Dataset Version 1; October 18, 2021. https://doi.org/10.5061/dryad.wwpzgmskk.
84. WHO. 2001. *Putting Women First: Ethical and Safety Recommendations for Research on Domestic Violence against Women*. Department of Gender and Women's Health, Family and Community Health. https://www.who.int/gender-equity-rights/knowledge/who_fch_gwh_01.1/en/.
85. Hahn, J.W., McCormick, M.C., Silverman, J.G., Robinson, E.B. and Koenen, K.C. 2014. Examining the impact of disability status on intimate partner violence victimization in a population sample. *Journal of Interpersonal Violence*. 29(17); pp.3063-3085.
86. Van Der Heijden, I. and Dunkle, K. 2017. *What Works Evidence Review: Preventing violence against women and girls with disabilities in lower-and middle-income countries (LMICs)*. UKAIDS. https://www.whatworks.co.za/resources/evidence-

reviews/item/349-what-works-evidence-review-preventing-violence-against-women-and-girls-with-disabilities-in-lower-and-middle-income-countries-lmics.

87. Kallakuri, S., Devarapalli, S., Tripathi, A.P., Patel, A. and Maulik, P.K. 2018. Common mental disorders and risk factors in rural India: baseline data from the SMART mental health project. *BJPsych Open*. 4(4); pp.192-198.
88. Kar, S.K., Sharma, E., Agarwal, V., Singh, S.K., Dalal, P.K., Singh, A., Gopalkrishna, G. and Rao, G.N. 2018. Prevalence and pattern of mental illnesses in Uttar Pradesh, India: Findings from the National Mental Health Survey 2015–16. *Asian Journal of Psychiatry*. 38; pp.45-52.
89. Sagar, R., Dandona, R., Gururaj, G., Dhaliwal, R.S., Singh, A., Ferrari, A., Dua, T., Ganguli, A., Varghese, M., Chakma, J.K. and Kumar, G.A. 2020. The burden of mental disorders across the states of India: The Global Burden of Disease Study 1990–2017. *The Lancet Psychiatry*. 7(2); pp.148-161.
90. Dembo, R.S., Mitra, M. and McKee, M. 2018. The psychological consequences of violence against people with disabilities. *Disability and Health Journal*. 11(3); pp.390-397.
91. Gudlavalleti, M.V.S., John, N., Allagh, K., Sagar, J., Kamalakannan, S., Ramachandra, S.S 2014. Access to health care and employment status of people with disabilities in South India, the SIDE (South India Disability Evidence) study. *BMC Public Health*. 14(1); p.1125.
92. Murthy, G.V.S., John, N., Sagar, J. . 2014. Reproductive health of women with and without disabilities in South India, the SIDE study (South India Disability Evidence) study: a case control study. *BMC Women's Health*. 14(1); p.146.
93. Bhattacharya, A., Camacho, D., Kimberly, L.L. and Lukens, E.P. 2019. Women's experiences and perceptions of depression in India: A metaethnography. *Qualitative Health Research*. 29(1); pp.80-95.
94. Malhotra, S. and Shah, R. 2015. Women and mental health in India: An overview. *Indian Journal of Psychiatry*. 57(Suppl 2); p.S205.
95. Subbaraman, R., Nolan, L., Shitole, T., Sawant, K., Shitole, S., Sood, K., Nanarkar, M., Ghannam, J., Betancourt, T.S., Bloom, D.E. and Patil-Deshmukh, A. 2014. The psychological toll of slum living in Mumbai, India: a mixed methods study. *Social Science & Medicine*. 119; pp.155-169.
96. Government of India. 2019. *Conducting the NFHS/Annual Health Surveys*. Ministry of Health and Family Welfare. https://pib.gov.in/PressReleaseIframePage.aspx?PRID=1576629.
97. Ahmad, J., Khan, N. and Mozumdar, A. 2019. Spousal Violence Against Women in India: A Social–Ecological Analysis Using Data from the National Family Health Survey 2015 to 2016. *Journal of Interpersonal Violence*. DOI: 10.1177/0886260519881530.
98. Ghai, A. 2019. Rethinking Disability in India. *Taylor & Francis*. London.
99. Ghosh, N. 2016. Negotiating Femininity: Lived Experiences of Women with Locomotor Disabilities in Bengal. In: Ghosh, N. (ed.). *Interrogating Disability in India*. 2016 pp. 127-144, Springer, New Delhi.
100. Goel, R. 2005. Sita's trousseau: Restorative justice, domestic violence, and South Asian culture. *Violence Against Women*. 11; 639-665.
101. Ahmed-Ghosh, H. 2004. Chattels of society: Domestic violence in India. *Violence against women*. 10; 94-118.

For peer review only - http://bmjopen.bmj.com/site/about/guidelines.xhtml

102. Rowan, K., Mumford, E. and Clark, C.J. . 2018. Is women's empowerment associated with help-seeking for spousal violence in India? *Journal of interpersonal violence*. 33; 1519-1548.
103. WHO. 2009. *Changing cultural and social norms supportive of violence behaviour. Series of briefings on violence prevention: the evidence*. https://www.who.int/violence_injury_prevention/violence/norms.pdf.
104. Nair, N., Daruwalla, N., Osrin, D., Rath, S., Gagrai, S., Sahu, R., Pradhan, H., De, M., Ambavkar, G., Das, N. and Dungdung, G.P. . 2020. Community mobilisation to prevent violence against women and girls in eastern India through participatory learning and action with women's groups facilitated by accredited social health activists: a before-and-after pilot study. *BMC International Health and Human Rights*. 20(1), pp.1-12.
105. Ellsberg, M., Arango, D.J., Morton, M., Gennari, F., Kiplesund, S., Contreras, M. and Watts, C. . 2015. Prevention of violence against women and girls: what does the evidence say? *The Lancet*. 385(9977), pp.1555-1566.
106. Sharma, S. and Sivakami, M. 2019. Sexual and reproductive health concerns of persons with disability in india: An issue of deep-rooted silence. *Journal of Biosocial Science*. 51(2); pp.225-243.
107. Asthana, A. 2017. Women in Distress: A Study on the Widowed, Divorced and Separated Women in Surguja (Chhattisgarh, India). *Divorced and Separated Women in Surguja (Chhattisgarh, India)*. https://dx.doi.org/10.2139/ssrn.3223334.
108. Government of India. 2005. *The Protection of Women from Domestic Violence Act, 2005*. Ministry of Law and Justice. https://legislative.gov.in/actsofparliamentfromtheyear/protection-women-domestic-violence-act-2005.
109. Government of India. 2016b. *The Rights of Persons with Disability Act, 2016*. Ministry of Law and Justice. http://legislative.gov.in/actsofparliamentfromtheyear/rights-persons-disabilities-act-2016.
110. Government of India. 2017. *The Mental Healthcare Act, 2017*. Ministry of Law and Justice. https://www.prsindia.org/uploads/media/Mental%20Health/Mental%20Healthcare%20Act,%202017.pdf.
111. Math, S.B., Gowda, G.S., Basavaraju, V., Manjunatha, N., Kumar, C.N., Philip, S. and Gowda, M. 2019. The rights of persons with disability act, 2016: Challenges and opportunities. *Indian Journal of Psychiatry*. 61(Suppl 4); p.S809.
112. Mishra, A. and Galhotra, A. 2018. Mental healthcare Act 2017: Need to wait and watch. *International Journal of Applied and Basic Medical Research*. 8(2), p.67.
113. Balakrishnan, A., Kulkarni, K., Moirangthem, S., Kumar, C.N., Math, S.B. and Murthy, P. 2019. The rights of persons with disabilities Act 2016: Mental health implications. *Indian Journal of Psychological Medicine*. 41(2), pp.119-125.
114. Duffy, R.M., Gulati, G., Kasar, N., Paralikar, V., Narayan, C.L., Desousa, A., Goyal, N. and Kelly, B.D. 2019. Stigma, inclusion and India's Mental Healthcare Act 2017. *Journal of Public Mental Health*. Vol. 18 No. 3, pp. 199-205.
115. Maji, S., Bansod, S. and Singh, T. 2021. Domestic violence during COVID-19 pandemic: The case for Indian women. *Journal of Community & Applied Social Psychology*. https://doi.org/10.1002/casp.2501.
116. Manjoo, R. 2014. Report of the Special Rapporteur on violence against women, its causes and consequences. *United Nations Human Rights Council*. Twenty-sixth session.

For peer review only - http://bmjopen.bmj.com/site/about/guidelines.xhtml

https://www.ohchr.org/en/hrbodies/hrc/regularsessions/session26/documents/a-hrc-26-38-add1_en.doc.
117. Tulsyan, A. 2016. Protection of Women from Domestic Violence Act 2005: Lessons from a Decade of Implementation. *Oxfam India*. https://www.oxfamindia.org/sites/default/files/Protection-of-women-from-domestic-violence-act-2005.pdf.

Footnote reference

- Shakespeare, T., 2006. The social model of disability. *The Disability Studies Reader*, *2*, pp.197-204.

For peer review only

Text (English)
What is your religion?
Hindu
Muslim
Christian
Sikh
Buddhist/Neo-Buddhist
Jain
Parsi/Zoroastrian
No religion/did not say
What is your caste?
Open/General
OBC
Scheduled caste (SC)
Scheduled tribe (ST)
None of these
What kind of house do you live in?
Kachha (non-concrete)
Pucca (concrete)
Mixed concrete and non-concrete
Other
Is the house you live in with your family rented or your own?
Own
Rented
Other
What type of toilet facility do you use?
Private or individual
Public
Paid toilet
Charity toilet
Open defecation
Other
***Now I would like to ask you about some important aspects of a woman's life. You [illegible]
What is your age?
What is your current marital status?
Unmarried
Married, Gauna not performed
Currently married
Living with partner
Separated
Divorced
Widowed/Widower
Answer refused
What is the highest level of education you have completed?
No formal education
Primary (1-5th standard)

For peer review only

Middle (6-8th standard)
High school (9-10th standard)
Senior school (11-12th standard)
Undergraduate
Graduate
Incomplete postgraduate
Postgraduate or higher
Other
WG-SS
***Now I am going to ask you if you have any type of difficulty. Please reply which des

Do you have difficulty seeing, even if wearing glasses?
No - no difficulty
Yes – some difficulty
Yes – a lot of difficulty
Cannot do at all
Do you have difficulty hearing, even if using a hearing aid?
Do you have difficulty walking or climbing steps?
Do you have difficulty remembering or concentrating?
Do you have difficulty (with self-care such as) washing all over or dressing?
**Using your usual (customary) language, do you have difficulty communicating, for ex
Respondent's livelihood
***As you know, some women take up jobs for which they are paid in cash or kind. Ot
No
Yes
What kind of work do you mainly do?
Home-based earnings *[stitching, pani-puri making, embroidery, snacks, gota/sitar
Vendor job *[fish selling, vegetable selling, flower/garland selling, snacks selling]*
Shop, parlor, saloon owner
Driver-Taxi/Auto/Cab/Bus
House maid, sweeper, construction or agriculture labour etc.
Job/service [work in mall/shops. Parlor/Saloon, executive, private job etc.]
Salaried job, consultant, executive, doctor, nurse
Other
Husband/ Partner's information
How old is (was) your husband/parner?
**What is the highest level of education your husband/partner has (had) completed?*
No formal education
Primary (1-5th standard)
Middle (6-8th standard)
High school (9-10th standard)
Senior school (11-12th standard)
Undergraduate
Graduate
Incomplete postgraduate
Postgraduate or higher

For peer review only

Other
Don't know
Is (was) your husband/partner working?
No
Yes
Don’t Know
What kind of work does he (did he) mainly do?
Home-based earnings *[stitching, pani-puri making, embroidery, snacks, gota/sitar
Vendor job [fish selling, vegetable selling, flower/garland selling, snacks selling]
Shop owner [Parlor, Saloon, Grocery, Pan, Meat etc.]
Driver-Taxi/Auto/Cab/Bus etc.]
House worker, sweeper, construction or agriculture labour etc.
Job/service [work in mall/shops. Parlor/Saloon, executive, private job etc.]
Salaried job, consultant, executive, doctor, nurse, teacher
Don’t Know
Other
Does (did) your husband/partner drink alcohol or take drugs?
No
Yes
Don’t Know
How often in a month does (did) he take it?
Sometimes
Regularly
Don’t Know
Now I would like to ask about all the births you have had during your life.
Have you ever been pregnant?
No
Yes
Have you ever given birth to a child?
No
Yes
Do you have any (live) children?
No
Yes
Number of living son(s)
Number of living daughter(s)

Household assets
Which of these things do you have in your home?
Mattress
Chair
Sofa-set
Cot/bed (Sofa bed)
Table
Almirah
Pressure cooker

For peer review only

Clock/watch
Metered Electricity
Radio/transistor
Electric fan
Cooler
AC
Normal TV
LED or LCD TV
Basic mobile phone
Smart phone
Computer (desktop)
Laptop
Induction cooker
Refrigerator/fridge
Mixer-grinder
Water purifier
Washing machine
Sewing machine
Water-pump/motor
Cycle
Bike/scooter/scooty (two-wheeler)
Car
Auto or taxi
Geyser
Tap drinking water
Do you drink alcohol or take any drug?
No
Yes
How often in a month do you take it?
Sometimes
Regularly
PHQ9
Over the last 2 weeks, how often have you been bothered by any of the following problems?
Little interest or pleasure in doing things
Not at all sure
Several days
Over half the days
Nearly every day
Feeling down, depressed, or hopeless
Trouble falling or staying asleep, or sleeping too much
Feeling tired or having little energy
Poor appetite or overeating
**Feeling bad about yourself or that you are a failure or have let yourself or your fami
**Trouble concentrating on things, such as reading the newspaper or watching televis
**Moving or speaking so slowly that other people could have noticed. Or the opposite

For peer review only

Thoughts that you would be better off dead, or of hurting yourself.
GAD7
***Over the last 2 weeks, how often have you been bothered
by any of the following problems?***
Feeling nervous, anxious, or on edge
Not at all sure
Several days
Over half the days
Nearly every day
Not being able to stop or control worrying
Worrying too much about different things
Trouble relaxing
Being so restless that it's hard to sit still
Becoming easily annoyed or irritable
Feeling afraid as if something awful might happen
Suicidal ideation
Apart from in the last two weeks, have you ever thought about ending your life?
Never
Yes, in the last 6 months
Yes, in the last year
Yes more than a year ago,
Have you ever tried to take your life?
Emotional violence
*Now I am going to ask you about some situations which happen to some women. Ple
Has anyone in your family ever insulted you or make you feel bad about yourself?
Yes
No
How often has this happened to you?
Once in the last 12 months
Sometimes in the last 12 months
Many times in the last 12 months
In the last 15 days
None of the above
Who mainly did this to you?
Husband (Partner)
Natal family members
Husband's family members
Other (stranger, neighbour, teacher, leader, police etc)
Has anyone in your family ever ignored you or treated you indifferently?
**Has anyone in your family ever said or done something to belittle or humiliate you i
**Has anyone in your family ever done things to scare you or intimidate you on purpo
**Has anyone in your family ever threatened to hurt you or someone you care about
Physical violence
***I am going to ask you about some situations which happen to some women. Please
**Has anyone in your family ever pushed you, shoved you, shaken you or done somet
Yes

For peer review only

No
How often has this happened to you?
Once in the last 12 months
Sometimes in the last 12 months
Many times in the last 12 months
In the last 15 days
None of the above
Who mainly did this to you?
Husband (Partner)
Natal family members
Husband's family members
Other (stranger, neighbour, teacher, leader, police etc)
**Has anyone in your family ever twisted your arm, banged your head or pulled your l
Has anyone in your family ever slapped, pinched or bitten you?
**Has anyone in your family ever hit or punched you with their fist or something else
Has anyone in your family ever kicked you, dragged you or beaten you up?
**Has anyone in your family ever thrown things at you or burned you with a cigarette,
**Has anyone in your family ever attacked you with a sharp object such as broken gla
**Has anyone in your family ever attacked or threatened you with a blunt object such
**Has anyone in your family ever attempted to suffocate, choke, hang you, or poison
Sexual violence
***I am going to ask you about some situations which happen to some women. Please
**Has anyone in your family ever physically forced you to have sexual intercourse witl
Yes
No
How often has this happened to you?
Once in the last 12 months
Sometimes in the last 12 months
Many times in the last 12 months
In the last 15 days
None of the above
Who mainly did this to you?
Husband (Partner)
Natal family members
Husband's family members
Other (stranger, neighbour, teacher, leader, police etc)
**Has anyone ever physically forced you to perform any other sexual acts *even you c
**Has anyone ever forced or threatened you in any other way to perform sexual acts
**Has anyone in your family ever forced you to replicate a sexual behaviour from porı

For peer review only

Type

Choice
Choice
Choice
Choice
Choice
Choice
Choice
Choice

Choice
Choice
Choice
Choice
Choice

Choice
Choice
Choice
Choice

Choice
Choice
Choice

Choice
Choice
Choice
Choice
Choice
Choice
may find some of the
Integer

Choice
Choice
Choice
Choice
Choice
Choice
Choice
Choice

Choice
Choice

For peer review only

Choice
Choice
Choice
Choice
Choice
Choice
Choice
Choice

scribes your conditior

Choice
Choice
Choice
Choice

xample, understandii

thers sell things, have
Choice
Choice

Choice
Choice
Choice
Choice
Choice
Choice
Choice
Choice

Integer
******
Choice
Choice
Choice
Choice
Choice
Choice
Choice
Choice
Choice

For peer review only

Choice
Choice

Choice
Choice
Choice

Choice
Choice
Choice
Choice
Choice
Choice
Choice
Choice
Choice

Choice
Choice
Choice

Choice
Choice
Choice
Label

Choice
Choice

Choice
Choice

Choice
Choice
Integer
Integer

Choice
Choice
Choice
Choice
Choice
Choice
Choice

For peer review only

Choice
Choice
Choice
Choice
Choice
Choice
Choice
Choice
Choice
Choice
Choice
Choice
Choice
Choice
Choice
Choice
Choice
Choice
Choice
Choice
Choice
Choice
Choice
Choice
Choice

Choice
Choice

Choice
Choice

Choice
Choice
Choice
Choice
As 171

ily down.**
sion**
being so figety or re

For peer review only

Choice
Choice
Choice
Choice
As 186
As 186
As 186
As 186
As 186
As 186

Choice
Choice
Choice
Choice

ease tell me if these a

Choice
Choice

Choice
Choice
Choice
Choice
Choice

Choice
Choice
Choice
Choice
As 209
As 209
As 209
As 209

e tell me if anyone ha
thing to hurt you?**
Choice

For peer review only

Choice

Choice
Choice
Choice
Choice
Choice

Choice
Choice
Choice
Choice
As 226
As 226
As 226
As 226
As 226
As 226
As 226
As 226

e tell me if anyone ha
h him *even when yo
Choice
Choice

Choice
Choice
Choice
Choice
Choice

Choice
Choice
Choice
Choice
As 250
As 250
As 250

For peer review only

Text (Hindi)

आपका धर्म क्या है?

हदिू
मुस्लमि
ईसाई
सखि
बौद्ध / नव-बौद्ध
जैन
पारसी / पारसी
कोई धर्म नहीं/जानकारी नहीं

आपकी जात क्या हैं?

सामान्य
ओबीसी
अनुसूचति जात
अनुसूचति जनजात
इनमें से कोई भी नहीं

आप कसि तरह के घर में रहतें हैं?

कच्चा
पक्का
कच्चा एवं पक्का, दोनों
अन्य

आप जसि घर में अपने परविार के साथ रहतीं है क्या वह करिाए का है या आपका अपना घर है?

स्वयं का
करिाये का
अन्य

आप कसि प्रकार के शौचालय सुवधिा का प्रयोग करते हैं?

नजिी
सार्वजानकि
नजिी लेकनि भुगतान शौचालय
सार्वजानकि लेकनि भुगतान सौचालय
खुले में
अन्य

***अब मैं आपसे महलिाओं के जीवन के कुछ महत्वपूर्ण पहलुओं के बारे में सवाल पूछना चाहती हूँ. इनमें से कुछ प्र

आपकी (उत्तरदाता) की उम्र कतिनी उम्र है?

वर्तमान में आपकी (उत्तरदाता) वैवाहकि स्थति क्या है?

अववविाहति
शादी की पर गौना नहीं हुआ
शादीशुदा
साथी के साथ रहना
पत से अलग
तलाकशुदा
वधिवा/ वधिुर
उत्तर से इनकार कर दयिा

आपकी उच्चतम शक्िषा क्या है?

कोई औपचारकि शक्िषा नहीं
प्राइमरी (1-5th standard)

For peer review only

मडिलि (6-8th standard)
हाई स्कूल (9-10th standard)
सीनयिर स्कूल (11-12th standard)
ग्रेजुएशन पूरा नहीं
ग्रेजुएशन
पोस्ट ग्रेजुएशन पूरा नहीं
पोस्ट ग्रेजुएशन और आगे
अन्य
वकिलांगता
***अब मै आपसे कुछ कठनिाइयों के बारे में पूछने जा रहीं हूँ. यदआिपको कसिी प्रकार की कठनिाई है तो, कृपया उ
क्या आपको देखने में परेशानी होती है?
*(कृपया पूंछे कि्या वे चश्मा पहनने पहनते है? यदहिाँ तो क्या चश्मा पहनने के बाद भी उन्हें देखने में
नहीं देखने में कोई कठनिाई नहीं
हां - देखने में कुछ कठनिाई
हां - देखने में बहुत मुश्कलि
बल्किुल नहीं देख सकते
क्या आपको सुनने में कठनिाई होती है (भले ही आप सुनवाई सहायता का उपयोग करते है)?
क्या आपको चलने या चढ़ने में कठनिाई होती है?
क्या आपको चीजे याद रखने में या ध्यान केंद्रति करने में कठनिाई होती है?
क्या आपको स्वयं की देखभाल जैसे नहाने-धोने या कपडे पहनने कठनिाई होती है?
**अपनी सामान्य भाषा का उपयोग करते हुए, क्या आपको बात-चीत करने में कठनिाई होती है, उदाहरण के लिए,
आजीवकिा
***जैसा कआिप जानती हों, कुछ महलिायें ऐसे काम करती हैं जनिके लिए उन्हें नगद या कसिी चीज के रूप में भुगत
नहीं
हाँ
मुख्यतः आप कसि प्रकार का काम करती हों?
घर पर आधारति काम (आय) *[सहायता [सलिाई, पानी-पुरी बनाने, कढ़ाई, नाश्ते बनाने, गोटा / सीतारा टकगि
वक्रिेता नौकरी* [मछली बेचने, सब्जी की बक्रिी, फूल / माला बेचने, नाश्ते की बक्रिी]*
दुकान, पार्लर, सैलून आदके मालकि
ड्राइवर *[टैक्सी / ऑटो / टैक्सी / बस]*
घर नौकरानी, सफाई कर्मचारी, नरि्माण या कृषशि्रमकि आद
नौकरी / सेवा *[मॉल / दुकानों में काम करते हैं। पार्लर / सैलून, कार्यकारी, नजिी नौकरी आद]*
वेतनभोगी नौकरी, परामर्शदाता, अधकिारी, डॉक्टर, नर्स
अन्य
पत/साथी
आपके पत/साथी की उम्र कतिनी है (थी)?
आपके पत/साथी की उच्चतम शक्षिा क्या है (थी) ?
कोई औपचारकि शक्षिा नहीं
प्राइमरी (1-5th standard)
मडिलि (6-8th standard)
हाई स्कूल (9-10th standard)
सीनयिर स्कूल (11-12th standard)
ग्रेजुएशन पूरा नहीं
ग्रेजुएशन
पोस्ट ग्रेजुएशन पूरा नहीं
पोस्ट ग्रेजुएशन और आगे

अन्य
नहीं पता
क्या आपके पति/ साथी कोई रोजगार या काम करते हैं (थे)?
नहीं
हाँ
पता नहीं
मुख्यतः आपके पति/साथी किस प्रकार का काम/व्यवसाय करते हैं (थे)?
घर पर आधारित काम (आय) [सहायता [सिलाई, पानी-पुरी बनाने, कढ़ाई, नाश्ते बनाने, गोटा / सीतारा टकिंग]
विक्रेता नौकरी [मछली बेचने, सब्जी की बिक्री, फूल / माला बेचने, नाश्ते की बिक्री]
दुकान मालिक [पार्लर, सैलून, पान, किराना, मांस इत्यादि]
ड्राइवर [टैक्सी / ऑटो / टैक्सी / बस इत्यादि]
घर नौकरानी, सफाई कर्मचारी, निर्माण या कृषि श्रमिक इत्यादि]
नौकरी / सेवा [मॉल / दुकानों में काम करते हैं। पार्लर / सैलून, कार्यकारी, निजी नौकरी आदि]
वेतनभोगी नौकरी, परामर्शदाता, अधिकारी, डॉक्टर, नर्स, शिक्षक
पता नहीं
अन्य
क्या आपके पति/साथी किसी भी तरह के मादक पदार्थ या शराब पीते है (थे)?
नहीं
हाँ
पता नहीं
आपके पति/साथी एक महीने में कितनी बार नशा करते है (थे)?
कभी कभी
नियमित रूप से
पता नहीं
अब मै आपसे उन बच्चे के बारे में पूछना चाहूंगी जिनको आपने अपने जीवनकाल में जन्म दिया हैं.
क्या आप कभी गर्भवती हुई है?
नहीं
हाँ
क्या आपने कभी किसी बच्चे को जन्म दिया है?
नहीं
हाँ
क्या आपको कोई (जीवित) बच्चे है?
नहीं
हाँ
जीवित बेटों की संख्या
जीवित बेटियों की संख्या

हाउसहोल्ड की संपत्ति
आपके घर में इनमें से कौन से समान हैं?
गद्दे
कुर्सी
सोफा सेट
बेड (पलंग)/सोफा बेड
टेबल /मेज
अलमारी
प्रेशर कुकर

घड़ी
मीटर बजिली
रेडिओ / ट्रांजसि्टर
बजिली का पंखा
कूलर
ए. सी.
सामान्य टीवी
एल.इ.डी या एल.सी.डी टी. वी.
साधारण मोबाइल फ़ोन
स्मार्ट फ़ोन
कम्पूटर
लैपटॉप
इंडक्शन कुकर
फ्रजि
मक्सिर
वाटर प्यूरीफायर
वॉशगि मशीन
सलिाई मशीन
पानी का पंप / मोटर
साइकल
बाइक /स्कूटर/स्कूटी/(अन्य दुपहयिा)
कार
रक्शिा/ टैक्सी
ग्रीज़र
नल का पानी
क्या आप कसिी भी तरह के मादक पदार्थ या शराब पीते हैं?
नहीं
हाँ
आप इसे महीने में कतिनी बार लेते हैं ?
कभी कभी
नयिमति रूप से
अवसाद (PHQ9)

पछिले 2 हफ्तों (15 दनिों) में, आपको कतिनी बार नमि्नलखिति समस्याओं से परेशान कयिा गया है?
कोई भी काम करने में कोई ख़ुशी नहीं होती और न ही कोई इच्छा (चाहत) होती है.
एक भी दनि नहीं
कई दनि
ज्यादातर दनिों में
लगभग हर रोज
उदास रहना या हर वक्त नराशा में रहना.
नींद का न आना या फरि सोये रहने में मुश्कलि होना या बहुत ज्यादा सोते रहना.
थकावट सी रहना या कमजोरी महसूस करना.
खाने को दलि नहीं करना या जरूरत से ज्यादा खाना.
**खुद के बारे में बुरा महसूस करना या खुद को असफल समझना. या खुद को नीचा समझना क्योकिआपने परविार व
कसिी भी काम में मन नहीं लगना जैसे कि कुछ पढना, लखिना या टी.वी. देखना इत्यादि.
**इतने धीरे (अहसि्ता) चलना या इतने धीरे बातें करना कि लोग इस बात को ध्यान (नोटसि) देने लगे या फरि इस

For peer review only

**आप यह सोचतीं हैं कि ऐसी जिन्दगी से तो मौत ही अच्छी है या आपने खुद को नुकसान पहुचने के बारे में सोचा.*
चिंता (GAD7)

पिछले 2 हफ्तों (15 दिनों) में, आपको कितनी बार निम्नलिखित समस्याओं से परेशान किया गया है?
घबराहट या परेशनी महसूस करना.
एक भी दिन नहीं
कई दिन
ज्यादातर दिनों में
लगभग हर रोज
चिंता को रोक न पाना
हर बात के लिए ज्यादा चिंतित रहना.
आराम न कर पाना या मुश्किल होना.
इतनी बेचैनी होना की आप के लिए एक जगह पर आराम से बैठ पाना मुश्किल हों
मामूली सी बात पर बेचैन या गुस्सा या नाराज हो जाना.
कोई भयकर घटना घटने का डर या भय महसूस होता हो.
आत्महत्या करने की प्रवत्ति
क्या आपने अपने जीवन में कभी भी आत्महत्या करने के बारे में सोचा है?
कभी नहीं
हाँ, पिछले 6 महीने में
हाँ, पिछले एक साल में
हाँ, पिछले एक साल से पहले
क्या आपने अपने जीवन में कभी भी आत्महत्या (खुदखुशी) करने की कोशिश की है?
भावनात्मक हिंसा
***अब मैं आपको कुछ परिस्थितियों के बारे में पूछने जा रही हूं जो कुछ महिलाओं के साथ होती हैं. कृपया मुझे बताएं
**क्या आपके परिवार में किसी व्यक्ति ने कभी भी, आपका अपमान किया है या आपको आपके बारे में बुरा महसूस
हाँ
नहीं
आपके साथ ऐसा कितनी बार हुआ है?
पिछले 12 महीनों मे एक बार
पिछले 12 महीनों मे कई बार
पिछले 12 महीनों मे बहुत बार
पिछले 15 दिनों में
इनमें से कोई नहीं
आपके साथ यह करने वाला मुख्य व्यक्ति कौन था?
पति (साथी)
मायके (के लोग) वाले
ससुराल (के लोग) वाले
अन्य (अजनबी, पड़ोसी, शिक्षक, नेता, पुलिस आदि)
**क्या आपके परिवार में किसी व्यक्ति ने कभी भी, आपको अनदेखा किया या आपके साथ बत्तमीजी से बर्ताव कि
**क्या आपके परिवार के किसी भी सदस्य नें कभी भी, आपको अन्य लोगो के सामने नीचा दिखाया या आपके साथ
**क्या आपके परिवार के किसी भी सदस्य नें कभी भी, आपको डराने के लिए जानबूज कर कुछ किया है (जैसे आपक
**क्या आपके परिवार के किसी भी सदस्य नें कभी भी, आपको या आपके किसी अपने/ नजदीकी व्यक्ति को चोट या
शारीरिक हिंसा
***मैं आपको कुछ स्थितियों के बारे में पूछने जा रही हूं जो कुछ महिलाओं के साथ होती हैं. कृपया मुझे बताओ कि क्
**क्या आपके परिवार के किसी भी सदस्य नें कभी भी आपको धक्का दिया, झंझोडा या आपकी तरफ कोई चीज़ उठ
हाँ

नहीं
आपके साथ ऐसा कतिनी बार हुआ है?
पछिले 12 महीनों मे एक बार
पछिले 12 महीनों मे कई बार
पछिले 12 महीनों मे बहुत बार
पछिले 15 दनिों में
इनमें से कोई नहीं
आपके साथ यह करने वाला मुख्य व्यक्त‍ि कौन था?
पत‍ि (साथी)
मायके (के लोग) वाले
ससुराल (के लोग) वाले
अन्य (अजनबी, पड़ोसी, शक्ि‍षक, नेता, पुलसि आद‍ि)
**क्या आपके परविार के कसिी भी सदस्य नें कभी भी आपकी बांह मरोड़ी या आपका सरि पटका या आपके बाल खीं
क्या आपके परविार के कसिी भी सदस्य नें कभी भी, आपको थप्पड़ मारा या चमिटी काटा या दांतों से काटा?
**क्या आपके परविार के कसिी भी सदस्य नें कभी भी, आपको मुक्के मारे ग़ा कसिी चीज़ से मारा ज़सिसे आपको चो
क्या आपके परविार के कसिी भी सदस्य नें कभी भी, आपको लात मारी, आपको घसीटा या आपको पीटा है?
**क्या आपके परविार के कसिी भी सदस्य नें कभी भी, आपके ऊपर चीजों को फेंका या आप पर सगिरेट / बीड़ी, मा
**क्या आपके परविार के कसिी भी सदस्य नें कभी भी, आप पर कसिी तेज़ धार वाले साधन या हथयिार जैसे टूटे कांच
**क्या आपके परविार के कसिी भी सदस्य नें कभी भी, आपको बेल्ट, पत्थर, झाड़ू या बेलन जैसी या कसिी भारी वस
**क्या आपके परविार के कसिी भी सदस्य नें कभी भी, आपका दम या गला घोंटने या आपको फांसी लगाने या जहर
लैंगकि हसिा
***मै आपसे कुछ उन स्थतयिो के बारे मे पूछने जा रही हूँ जो कुछ महलियों के साथ होती है. आप मुझे बताये की इन
**क्या आपके परविार के कसिी भी सदस्य नें कभी भी, *आपके न चाहते हुए भी*, शारीरकि बल के प्रयोग से आपके
हाँ
नहीं
आपके साथ ऐसा कतिनी बार हुआ है?
पछिले 12 महीनों मे एक बार
पछिले 12 महीनों मे कई बार
पछिले 12 महीनों मे बहुत बार
पछिले 15 दनिों में
इनमें से कोई नहीं
आपके साथ यह करने वाला मुख्य व्यक्त‍ि कौन था?
पत‍ि (साथी)
मायके (के लोग) वाले
ससुराल (के लोग) वाले
अन्य (अजनबी, पड़ोसी, शक्ि‍षक, नेता, पुलसि आद‍ि)
**क्या आपके परविार के कसिी भी सदस्य नें कभी भी, *आपके न चाहते हुए भी*, शारीरकि बल से आपको सेक्स/यौ
**क्या आपके परविार के कसिी भी सदस्य नें कभी भी, *आपके न चाहते हुए भी*, डरा-धमका कर या कोई और तरीक
**क्या आपके परविार के कसिी भी सदस्य नें कभी भी, *आपके न चाहते हुए भी*, अश्लील साहत्यि या अन्य यौन स

For peer review only

[illegible]स्ती में महिलाओं की स्थिति को और अच्छे से समझने में के लिए आपके उत्तर महत्वपूर्ण हैं. मैं आपको भरोसा

For peer review only - http://bmjopen.bmj.com/site/about/guidelines.xhtml

· वर्णन करता हो.***

ातों को दूसरों को समझाने में?**

ाती हैं, छोटा व्यापार करती हैं, अथवा घर की खेती या घर के व्यापार में हाथ बंटाती हैं. पछिले 12 महीनों में, क्

For peer review only

For peer review only

For peer review only

[illegible] बेचैनी में रहते हों.**

ने आपको अपमानति लगे?**
को तोडा इत्यादि)?
ने की धमकी दी?**

हुआ है.***

ालाया?**
ुमला कयिा या करने की धमकी दी?**

?***
ा मजबूर कयिा?**

को मजबूर कयिा?**
ने के को मजबूर कयिा?**
र (हरकत) को दोहराने के लिए मजबूर कयिा?**

For peer review only

देती हूँ कि आपके उत्तर पूरी तरह से गोपनीय रखे जायेंगे और उन्हें किसी को भी बताया नहीं जाएगा, और आपवे

For peer review only

क्या आपने इनमें से कोई काम या कोई और काम किया है?***

For peer review only

For peer review only

For peer review only

For peer review only

For peer review only

के परविार में से भी कोई भी नहीं जान पायेगा कि आपसे ये सरे सवाल पूछे गए हैं. यदि आप मेरे द्वारा पूछे गये क

For peer review only

For peer review only

For peer review only

For peer review only

For peer review only

सी भी सवाल का जवाब नहीं चाहतीं हैं कृपया मुझे बताये, मै आगे के सवालों पर बढ़ जाउंगी.***

For peer review only

For peer review only

For peer review only

For peer review only

For peer review only

For peer review only

For peer review only

STROBE Statement—Checklist of items that should be included in reports of ***cross-sectional studies***

	Item No	Recommendation	Page No
**Title and abstract**	1	(*a*) Indicate the study's design with a commonly used term in the title or the abstract	1
		(*b*) Provide in the abstract an informative and balanced summary of what was done and what was found	2
**Introduction**			
Background/rationale	2	Explain the scientific background and rationale for the investigation being reported	4-6
Objectives	3	State specific objectives, including any prespecified hypotheses	7
**Methods**			
Study design	4	Present key elements of study design early in the paper	7
Setting	5	Describe the setting, locations, and relevant dates, including periods of recruitment, exposure, follow-up, and data collection	7
Participants	6	(*a*) Give the eligibility criteria, and the sources and methods of selection of participants	8
Variables	7	Clearly define all outcomes, exposures, predictors, potential confounders, and effect modifiers. Give diagnostic criteria, if applicable	9-11
Data sources/ measurement	8*	For each variable of interest, give sources of data and details of methods of assessment (measurement). Describe comparability of assessment methods if there is more than one group	*8*
Bias	9	Describe any efforts to address potential sources of bias	12
Study size	10	Explain how the study size was arrived at	8
Quantitative variables	11	Explain how quantitative variables were handled in the analyses. If applicable, describe which groupings were chosen and why	10
Statistical methods	12	(*a*) Describe all statistical methods, including those used to control for confounding	11
		(*b*) Describe any methods used to examine subgroups and interactions	
		(*c*) Explain how missing data were addressed	
		(*d*) If applicable, describe analytical methods taking account of sampling strategy	
		(*e*) Describe any sensitivity analyses	
**Results**			
Participants	13*	(a) Report numbers of individuals at each stage of study—eg numbers potentially eligible, examined for eligibility, confirmed eligible, included in the study, completing follow-up, and analysed	13
		(b) Give reasons for non-participation at each stage	12
		(c) Consider use of a flow diagram	
Descriptive data	14*	(a) Give characteristics of study participants (eg demographic, clinical, social) and information on exposures and potential confounders	13-14
		(b) Indicate number of participants with missing data for each variable of interest	
Outcome data	15*	Report numbers of outcome events or summary measures	15-20

For peer review only

For peer review only - http://bmjopen.bmj.com/site/about/guidelines.xhtml

Main results	16	(*a*) Give unadjusted estimates and, if applicable, confounder-adjusted estimates and their precision (eg, 95% confidence interval). Make clear which confounders were adjusted for and why they were included	15-20
		(*b*) Report category boundaries when continuous variables were categorized	
		(*c*) If relevant, consider translating estimates of relative risk into absolute risk for a meaningful time period	
Other analyses	17	Report other analyses done—eg analyses of subgroups and interactions, and sensitivity analyses	
**Discussion**			
Key results	18	Summarise key results with reference to study objectives	21
Limitations	19	Discuss limitations of the study, taking into account sources of potential bias or imprecision. Discuss both direction and magnitude of any potential bias	25
Interpretation	20	Give a cautious overall interpretation of results considering objectives, limitations, multiplicity of analyses, results from similar studies, and other relevant evidence	21-23
Generalisability	21	Discuss the generalisability (external validity) of the study results	21-23
**Other information**			
Funding	22	Give the source of funding and the role of the funders for the present study and, if applicable, for the original study on which the present article is based	27

*Give information separately for exposed and unexposed groups.

Note: An Explanation and Elaboration article discusses each checklist item and gives methodological background and published examples of transparent reporting. The STROBE checklist is best used in conjunction with this article (freely available on the Web sites of PLoS Medicine at http://www.plosmedicine.org/, Annals of Internal Medicine at http://www.annals.org/, and Epidemiology at http://www.epidem.com/). Information on the STROBE Initiative is available at www.strobe-statement.org.

For peer review only

BMJ Open

Intimate partner violence against women with disability and associated mental health concerns: a cross-sectional survey in Mumbai, India

Journal:	*BMJ Open*
Manuscript ID	bmjopen-2021-056475.R2
Article Type:	Original research
Date Submitted by the Author:	12-Feb-2022
Complete List of Authors:	Riley, Andrew; University of Liverpool, Institute of Population Health<br>Daruwalla, Nayreen; SNEHA<br>Kanougiya, Suman; Tata Institute of Social Sciences (TISS)<br>Gupta, Apoorwa; SNEHA<br>Wickenden, Mary; University of Sussex<br>Osrin, David; University College London
**Primary Subject Heading**:	Public health
Secondary Subject Heading:	Mental health, Global health
Keywords:	MENTAL HEALTH, PUBLIC HEALTH, SOCIAL MEDICINE

SCHOLARONE™
Manuscripts

I, the Submitting Author has the right to grant and does grant on behalf of all authors of the Work (as defined in the below author licence), an exclusive licence and/or a non-exclusive licence for contributions from authors who are: i) UK Crown employees; ii) where BMJ has agreed a CC-BY licence shall apply, and/or iii) in accordance with the terms applicable for US Federal Government officers or employees acting as part of their official duties; on a worldwide, perpetual, irrevocable, royalty-free basis to BMJ Publishing Group Ltd ("BMJ") its licensees and where the relevant Journal is co-owned by BMJ to the co-owners of the Journal, to publish the Work in this journal and any other BMJ products and to exploit all rights, as set out in our licence.

The Submitting Author accepts and understands that any supply made under these terms is made by BMJ to the Submitting Author unless you are acting as an employee on behalf of your employer or a postgraduate student of an affiliated institution which is paying any applicable article publishing charge ("APC") for Open Access articles. Where the Submitting Author wishes to make the Work available on an Open Access basis (and intends to pay the relevant APC), the terms of reuse of such Open Access shall be governed by a Creative Commons licence – details of these licences and which Creative Commons licence will apply to this Work are set out in our licence referred to above.

Other than as permitted in any relevant BMJ Author's Self Archiving Policies, I confirm this Work has not been accepted for publication elsewhere, is not being considered for publication elsewhere and does not duplicate material already published. I confirm all authors consent to publication of this Work and authorise the granting of this licence.

For peer review only

Intimate partner violence against women with disability and associated mental health concerns: a cross-sectional survey in Mumbai, India

Authors

1. Andrew Riley *Corresponding author*

   Institute of Population Health, University of Liverpool, Liverpool, L69 3BX, UK

   andrew.riley@liverpool.ac.uk

2. Nayreen Daruwalla

   Program on Prevention of Violence Against Women and Children, SNEHA, Mumbai, Maharashtra, 400017, India

   nayreen@snehamumbai.org

3. Suman Kanougiya

   School of Health Systems Studies (SHSS), Tata Institute of Social Sciences (TISS), Mumbai, Maharashtra, India

   suman.kanougiya@gmail.com

4. Apoorwa Deepak Gupta

   Program on Prevention of Violence Against Women and Children, SNEHA, Mumbai, Maharashtra, 400017, India

   apoorwa.gupta@snehamumbai.org

5. Mary Wickenden

   Institute of Development Studies, University of Sussex, Library Rd, Falmer, Brighton BN1 9RE, UK

   M.Wickenden@ids.ac.uk

6. David Osrin

For peer review only

Institute for Global Health, University College London,

London, WC1N IEH, UK

d.osrin@ucl.ac.uk

For peer review only

Abstract

Objectives

The risk of intimate partner violence (IPV) against women with disability is believed to be high. We aimed to compare the prevalence of past-year IPV against women with and without functional difficulties in urban informal settlements, to review its social determinants, and to explore its association with mental health.

Design

Cross-sectional survey

Setting

Fifty clusters within four informal settlements.

Participants

For peer review only - http://bmjopen.bmj.com/site/about/guidelines.xhtml

5122 women aged 18 to 49 years.

Primary and secondary outcome measures

We used the Washington Group Short Set of Questions to assess functional difficulties. IPV in the past year was described by binary composites of questions about physical, sexual, and emotional violence. We screened for symptoms of depression using the Patient Health Questionnaire 9 and of anxiety using the Generalized Anxiety Disorder 7. Multivariable logistic regression models examined associations between functional difficulties, IPV, and mental health.

Results

The 10% of participants who screened positive for functional disability had greater odds of experiencing physical or sexual IPV (Adjusted Odds Ratio (AOR) 1.68, 95% CI 1.23, 2.29) and emotional IPV (1.52, CI 1.16, 2.00) than women who screened negative. Women who screened positive for functional disability had greater odds than women who screened negative of symptoms suggesting moderate or severe anxiety (AOR 2.50, CI 1.78, 3.49), depression (2.91, CI 2.13, 3.99), and suicidal thinking (AOR 1.94, CI 1.50, 2.50).

Conclusions

The burden of IPV fell disproportionately on women with functional difficulties, who were also more likely to screen positive for common mental disorder. Public health initiatives need to respond at local and national levels to address the overlapping and mutually reinforcing determinants of violence, while existing policy needs to be better utilised to ensure protection for the most vulnerable.

For peer review only

Keywords

Intimate partner violence; Domestic violence; Disability; India; Poverty areas

Strengths and limitations of this study

- Data came from a large cross-sectional survey of women in informal settlements.
- Data were collected by an organisation providing support for survivors of intimate partner violence.
- Functional difficulties were self-reported and not the primary focus of the survey.
- The cross-sectional nature of the study limits the possibility of causal inference.

For peer review only

Background

Approximately 15% of the world's population, around one billion people, live with a physical, intellectual, sensory, or mental health impairment that affects their daily lives and long-term health and wellbeing [1]. Disability is the product of attitudinal and environmental barriers that limit persons with functional difficulties from fully participating in society [2][1]. These barriers mean that, compared with non-disabled people, people with disability are less likely to be educated [3], financially secure [4], employed [5], have access to health services [1], have their health needs met [1, 6], and have their social needs met [7-9].

The double burden on women and girls with disability means that they are among the more marginalised and vulnerable in society, particularly when this burden intersects with multidimensional poverty [1]. Lacking physical and financial independence or access to socially inclusive services, disabled women who depend on partners and

[1] In this paper our approach to disability is in line with the social model which identifies disability as a social creation, 'a relationship between people with impairment and a disabling society' (Shakespeare, 2006, p.198), distinct from medical and individual models of disability. We therefore use the term 'disabled women', which denotes this position, interchangeably with 'women with disabilities'.

For peer review only - http://bmjopen.bmj.com/site/about/guidelines.xhtml

families for support are vulnerable to further social marginalisation and isolation and are at higher risk of physical violence and sexual abuse [10, 11], as well as other forms of abuse such as neglect, exploitation, and coercive control [12]. Irrespective of disability, violence against women occurs most commonly in the home, in the form of intimate partner violence (IPV) or non-partner domestic violence [13, 14]. Women with disability are believed to be at greater risk of these and other forms of violence. However, a dearth of population-level research [15], and the typically hidden nature of violence [16], mean that little is known of how disability status may lead to additional risks of IPV. Such research is important for informing IPV prevention programmes [17, 18], and for meeting the objectives of the United Nations Sustainable Development Goals.

In India, the 2011 national census [19] and the 2018 National Sample Survey (NSS) [20] both estimated disability prevalence at 2.2% (26.8 million people), although these are considered underestimates due to proxy reporting and insufficient consideration of disability [21, 22]. Other national estimates range from 8% by the World Bank [22], to 25% by the World Health Survey [1, 23]. More local estimates reflect different

For peer review only - http://bmjopen.bmj.com/site/about/guidelines.xhtml

approaches to classification and include 1.6% in Tamil Nadu State [24], 1.9% in a Delhi slum community [25] and 12.2% in Telangana State [26]. Disability is most common among older, rural women and members of scheduled tribes and scheduled castes [20, 27], while prevalence increases with age, among lower-socioeconomic groups and those without employment / the unemployed [24, 26]. Disabled women often report lower rates of marriage [20, 26], and higher rates of widowhood, separation, and divorce [20, 24].

The ubiquity of violence against women in India has been well reported in both popular media and population-level research. The fourth National Family Health Survey (NFHS-4) reported that 30% of married women had experienced physical, sexual, or emotional violence in their lifetime and 26% in the past year [28]. Similar rates were reported in a nationally representative systematic review [29] and in urban and slum settings [30-34]. Violence against women with disabilities in India is less documented: to date, only three major studies to date have reported on it at population level. A survey in Mumbai involving women aged 15-49 years with functional impairments (visual, locomotor, hearing) found that 20% of 123 ever-married disabled women had

For peer review only - http://bmjopen.bmj.com/site/about/guidelines.xhtml

experienced past-year physical IPV, 23% emotional IPV (being insulted, humiliated or threatened), and 10% sexual IPV. Violence perpetrated by people other than intimate partners was experienced by 18% of ever-married and 23% of unmarried women, most commonly by mothers-in-law in the marital family and brothers in the natal [11].

In Karnataka, of 70 female interviewees with disability, 73% reported 'significant' violence (versus 'rare' or 'occasional') and 23% sexual violence in the previous 12 months, while 59% reported generalised violence and 10% sexual violence in their childhood. Around half of this violence was perpetrated by family members (46%) or by people outside the family (54%) [35]. In a study involving 729 women in Odisha State, 48% of those with learning difficulties and 23% with physical impairments reported experiencing domestic violence [36]. There are a number of limitations to these studies, notably their small sample sizes and, in the latter two, their imprecision in defining violence and the use of convenience sampling [35, 36]; however, their findings are consistent with other reports from the wider South Asia region [37-39].

For peer review only

For peer review only - http://bmjopen.bmj.com/site/about/guidelines.xhtml

For all women, violence increases the risk of serious injury and mortality [40] [41], HIV and sexually transmitted infections [42, 43], unintended pregnancy [44], reproductive health morbidity [45, 46] and a range of mental health concerns [47-49]. Among women with disabilities in India who have experienced violence, high levels of severe mental distress, depression, suicidal ideation and attempted suicide have been documented [11].

Given its impact on physical and mental health, as well as being a matter of individual and social justice, violence against disabled women is largely absent from the literature. This absence limits the capacity of health planners and policymakers to act, while also contributing to the ongoing marginalisation of disabled communities, experiences, and concerns.

Objectives

This study sought to address the gap in understanding of the sociodemographic circumstances of women with disabilities in urban India and their experience of intimate partner violence. We aimed to identify associations between individual

For peer review only - http://bmjopen.bmj.com/site/about/guidelines.xhtml

socioeconomic determinants and disability in women aged 18 to 49 years living in informal settlements in Mumbai and to test the hypothesis that women with functional difficulty/disability are more likely to have experienced recent intimate partner violence and have higher rates of common mental disorders.

Methods

Setting

Maharashtra ranks fourth among Indian states in the human development index [50], and women tend to have higher than the national averages on indicators of literacy, marriage age and workforce participation. Mumbai, Maharashtra's capital, has approximately 12.7 million residents, 41% of whom live in informal settlements [51, 52]. These are characterised by overcrowding, lack of tenure and insufficient living space, housing durability, and water and sanitation facilities [53, 54]. Women in these disadvantaged communities typically depend on relatives for housing, making them vulnerable to homelessness, poverty and violence [54].

Design

For peer review only

We used data collected by SNEHA (Society for Nutrition, Education and Health Action), a non-government organization working in Mumbai's disadvantaged communities to improve the health of women and children and address the burden of violence. The dataset came from a survey conducted before the implementation of a community-based intervention to prevent violence against women and girls through individual volunteers and groups [55]. The survey included 50 clusters of equal size, each of 500 dwellings, covering a population of 77,000 in four areas within major informal settlements.

Participants and Data collection

Data collection has been described in detail elsewhere [56]. Clusters were mapped and homes visited to identify potential participants. Criteria for inclusion were women aged 18-49 years resident in a study cluster, who consented to interview. One woman per household was sampled (around 100 participants per cluster). Data collectors visited participants to explain the survey, discuss the process, answer questions, and provide a participant information sheet. Signed consent to interview was obtained and participants were assured of data confidentiality.

For peer review only - http://bmjopen.bmj.com/site/about/guidelines.xhtml

Sixteen women interviewers collected data. They were graduates who had not worked in the mental health field and were selected after application and interview through open recruitment. They received three months of training, which included three days of training facilitated by an expert on disability awareness and the Washington Group Short Set of questions. For participants with visual impairment, the interviews were oral, as they were for all participants. For participants with hearing impairment, a communications specialist accompanied the interviewer. For participants with learning difficulties, we developed an alternative interview in a simple visual format that was used 14 times during the survey.

Participants were asked about demographic characteristics, socioeconomic indicators, their health, wellbeing, functional difficulty and disability status, common mental disorders, their experience of emotional, physical, and sexual domestic violence, injuries, help-seeking, support and disclosure, and spousal drug and alcohol use. All participant information was confidential and no names or identifiable details appear in outputs or analyses. Sources of questions included the National Family Health Survey (NFHS-4) [28], the Indian Family Violence and Control Scale [57], the

For peer review only - http://bmjopen.bmj.com/site/about/guidelines.xhtml

International Violence Against Women Survey [58] and the WHO multi-country study [59]. Questions were translated where necessary, back-translated, piloted, and refined (see Supplementary File 1).

Sample size

An estimate of prevalence from a cross-sectional sample of 5000 in a population of 125,000 would have a precision of around 1%. Within this, a comparison of two categories of determinant for 100 participants in each of 50 clusters would provide 80% power at 5% significance level to detect a difference of 6% in prevalence estimates of 10-20%.

Variables

Disability was assessed using the Washington Group Short Set of Questions (WG-SS), based on the International Classification of Functioning, Disability and Health (ICF) model of disability [60]. The questions cover six functional domains (*seeing, hearing, walking, memory, self-care, speaking),* with four categories of response (*no difficulty, some difficulty, a lot of difficulty, cannot do at all*) [60]. For this analysis, a participant was classified as having disability if they reported 'some difficulty' in at least

For peer review only - http://bmjopen.bmj.com/site/about/guidelines.xhtml

one domain. This cut-off is one of several possible approaches and has been used in a similar setting [37].

Definitions of violence follow the WHO Multi-Country Study [59] and are discussed in detail elsewhere [56]. We screened for anxiety, depression, and suicidal thinking as common mental disorders. Anxiety was screened for using the Generalized Anxiety Disorder 7-item (GAD-7) scale [61]. Depression was screened for using the Patient Health Questionnaire (PHQ-9) [62]. For both instruments, a cut-off score of ≥10 has been used as suggestive of clinically relevant conditions requiring further assessment [63]. In this study, variables were coded 'yes' for depression and anxiety if responses were recorded as moderate or severe. Suicidal thinking was coded 'yes' for lifetime positive responses.

Covariates corresponded to determinants of risk for IPV identified from the wider body of Indian IPV research undertaken in comparable settings with women whose disability status was not identified. Covariates were classified by level of occurrence, following Heise's ecologic framework for violence against women [64, 65]. This approach

For peer review only

conceptualises risk of violence as a function of mutually interacting personal, situational, and sociocultural factors across individual, relationship, household, community, and society levels.

We hypothesised that a woman's odds of experiencing IPV would increase with age [66] [67] [68], number of children [66] [40], and employment outside the home [69] [70] [71], and would decrease with years of education [32] [72] [40]. Partner use of alcohol or drugs was hypothesised to increase the odds [30] [73] [74] [67] [75], while no clear relationship between IPV and partner level of education was predicted [33] [76] [66] [77]. Lower socioeconomic status was considered as potentially increasing the odds of IPV [11] [33] [34] [40] [74] [78], as were faith [67] [78] and caste [79] [67], due to the association of some groups with lower socioeconomic status.

Associations between common mental disorders and sociodemographic characteristics were hypothesised to conform to a similar pattern due to their close association with IPV. Hypothetical determinants were modelled as independent variables in both univariable and multivariable analyses [80]. At the level of individual

For peer review only - http://bmjopen.bmj.com/site/about/guidelines.xhtml

women, age-group, marital status, parity, education and employment were entered as categorical variables. Categories of age and schooling were based on those used in other studies [37] [40] [42] [45]. Individual employment categories were recoded as either home-based or outside the home as IPV has been associated with women working outside the home in general rather than individual employment categories [69] [70] [71]. At 'partner' level, husband's schooling and employment were entered as categorical indicators and alcohol or drug use as binary. At 'household' level, faith, caste and socioeconomic quintile were entered as categorical indicators. Socioeconomic quintile was entered as a categorical rather than continuous variable to retain categorical consistency and because it was hypothesised that a reduction in odds would be observed in the wealthier quintiles. Quintiles were based on analysis of 22 individual household assets, with scores derived from standardized weights from the first component of a principal components analysis [81] [82].

Statistical analysis

The survey had a single-stage design with clustering and stratification. Data analyses were unweighted because the clusters were of similar size and each was sampled for

For peer review only - http://bmjopen.bmj.com/site/about/guidelines.xhtml

about 100 questionnaires. Sociodemographic factors were summarised and cross-tabulated using frequencies and percentages, and univariable logistic regression models used to test associations between each exposure variable and the outcome. 204 values were missing in the socioeconomic quintile variable and 317 in variables relating to husbands, which were accounted for in the regression analysis through listwise deletion. Variables with associations at $p<0.1$ were included in multivariable analysis. Multivariable logistic regression was used to examine associations between outcome variables (disability, IPV, depression, anxiety) and primary exposure variables, adjusting for confounders (individual demographic and socioeconomic characteristics). Post hoc Wald tests were performed to check models. All analyses used survey commands in Stata 15.1 (College Station, TX). The data are available online at Open Science Framework [dataset] [83], available from https://osf.io/zhtpw/.

Ethical considerations

The survey involved disclosing personal and sensitive information, which raises issues of interviewer behaviour, consent, data sharing, and privacy and confidentiality. All interviewers underwent appropriate training and WHO ethical and safety

For peer review only - http://bmjopen.bmj.com/site/about/guidelines.xhtml

recommendations were followed [84]. Interview processes were checked through random visits and systematic monitoring by project officers and supervisors.

During the interview, participants were made aware of the potentially sensitive nature of the questions about violence and consent was obtained before they began. Efforts were made to ensure privacy by prearranging visits at a preferred time and location. Participants were able to attend a local community office, but most were interviewed at home. If household members were unwilling to leave, researchers encouraged them to listen to some of the routine questions to be reassured of the survey's nature. Most were amenable to leaving, but when they were not, the interview was terminated and completed over up to three repeat visits

Safeguarding was paramount during the survey. All participants were given information on local counselling and crisis support services for themselves or other women, whether they reported violence or not. Service providers were made aware of the study and the potential for increased service use. Researchers were able to arrange referrals if requested. For those who disclosed experience of violence, duty

For peer review only

of care was of particular concern. Details of safeguarding measures and counselling services made available to survivors are described elsewhere [56].

Ethics approval

Data collection was approved by the UCL Research Ethics Committee (3546/003 27/09/2017) and the PUKAR (Partners for Urban Knowledge, Action and Research) Institutional Ethics Committee (25/12/2017) [55].

Patient and public involvement

Our research responds to the urgency of preventing violence and improving services for survivors, primarily through community-based programming in informal settlements. It is driven by survivors' needs, one of which is inclusion. We followed a protocol co-developed with community members to achieve maximal inclusion of women with functional difficulties. We discussed the survey questions with participants in a pilot phase and adapted them for subsequent use. Our findings will be shared through community women's groups, three of which meet monthly in each of the 50 informal settlement clusters.

For peer review only - http://bmjopen.bmj.com/site/about/guidelines.xhtml

Results

Interviewers approached 5277 households between 5th December 2017 and 28th March 2019. In 967 (5%) households there was no eligible female resident; in 592 households (11%) the interviewers could not achieve privacy across repeat visits; and in 155 (3%) households a potential participant declined interview. In total, 5122 interviews were conducted.

Disability prevalence

Table 1 summarises the type and frequency of functional difficulties reported by women. 10% had ‘some difficulty’ in at least one domain, of which the most common were visual (4%) and locomotor (5%). Across all domains, the number of participants reporting a lot of difficulty or no ability at all was low, each less than 1%.

Table 1: Frequencies of domains of functional difficulty among 5122 women aged 18-49 years living in informal settlements in Mumbai

	None	Some difficulty	A lot of difficulty	Cannot do at all

For peer review only

Functional difficulty	n (%)	n (%)	n (%)	n (%)
Seeing	4896 (96)	212 (4)	12 (<1)	2 (<1)
Hearing	5078 (99)	40 (<1)	3 (<1)	1 (<1)
Walking	4860 (95)	234 (5)	25 (<1)	3 (<1)
Memory	4988 (97)	123 (2)	11 (<1)	0 (0)
Self-care	5108 (>99)	10 (<1)	4 (<1)	0 (0)
Speaking	5109 (>99)	4 (<1)	9 (<1)	0 (0)

Washington Group Disability prevalence assessment	n (%)
At least one domain *some difficulty*	505 (10)
At least one domain *a lot of difficulty*	63 (1)
At least one domain *cannot at all*	6 (<1)
At least one domain *a lot of difficulty* or *cannot at all* or at least *some difficulty* in two domains	178 (3)

For peer review only

Women's sociodemographic profiles and disability

Most women were in their 20s or 30s (80%), currently married (92%) and with one or two children (51%). A fifth of women (19%) reported having no schooling, while 44% had had six to 10 years. Having no remunerated work was common (76%), while those who had an income tended to work from home (15%). In unadjusted analyses, greater odds of disability were observed in women over 40 (odds ratio 2.7; 95% confidence interval 1.3, 5.5), women who were separated or divorced (3.3; 1.9, 5.6), or widowed

For peer review only - http://bmjopen.bmj.com/site/about/guidelines.xhtml

(3.6; 2.2, 5.9), and women who had three or more children (1.9; 1.4, 2.4). Attending school for at least six years was associated with lesser odds of disability than no schooling at all (0.8; 0.6, 0.9). Women who earned in the home had greater odds of disability than women who did not (1.5; 1.1, 2.0), as did women from Muslim rather than Hindu families (1.4; 1.1, 1.8).

Associations between disability and past-year IPV

Table 2 presents associations between women's functional difficulty and past-year physical, sexual, and emotional IPV. Of 5122 women, 628 reported suffering physical or sexual IPV in the last year (12%) and 606 had suffered emotional IPV (12%). These proportions were greater for women with disability: 84 of 505 who reported at least some functional difficulty had suffered physical or sexual IPV (17%) and 83 emotional IPV (16%). We had information on socioeconomic asset score for 4918 women who had ever been married, and on their husbands for 4805 women whose husbands were alive. After adjustment for age, marital status, parity, woman's and husband's schooling, woman's and husband's employment, religion, and husband's use of alcohol or drugs, these differences were manifest in adjusted odds ratios of 1.68 (95% CI 1.23, 2.29) for physical or sexual and 1.52 (1.16, 2.00) for emotional IPV.

For peer review only - http://bmjopen.bmj.com/site/about/guidelines.xhtml

Table 2: Associations between experience of intimate partner violence (physical or sexual IPV, emotional IPV) in the preceding year, disability, and sociodemographic characteristics, for 5122 women aged 18-49 years living in informal settlements in Mumbai

Characteristic (n)	Physical or sexual IPV in last year							Emotional IPV in last year						
	No n (%)	Yes n (%)	OR	(95% CI)	p-value	Adjusted OR	(95% CI)	No n (%)	Yes n (%)	OR	(95% CI)	p-value	Adjusted OR	(95% CI)
Disability														
None	4073 (88)	544 (12)	1			1		4093 (89)	524 (11)	1			1	
At least some difficulty	421 (83)	84 (17)	1.49	1.24, 1.99	0.007	1.68	1.23, 2.29	422 (84)	83 (16)	1.54	1.20, 1.97	0.001	1.52	1.16, 2.00
All	4494 (88)	628 (12)						4515 (88)	607 (12)					
Woman's age (years)														
<20	104 (90)	11 (10)	1			1		108 (94)	7 (6)	1			-	-
20-29	1747 (86)	291 (14)	1.57	0.54, 4.64		0.82	0.26, 2.56	1782 (87)	256 (13)	2.22	0.80, 6.16		-	-
30-39	1808 (88)	248 (12)	1.30	0.43, 3.88		0.57	0.18, 1.86	1812 (88)	243 (12)	2.07	0.74, 5.81		-	-
>40	835 (91)	78 (9)	0.88	0.28, 2.75	<0.001	0.39	0.11, 1.36	812 (89)	101 (11)	1.92	0.69, 5.34	0.329	-	-
All	4494 (88)	628 (12)						4515 (88)	607 (12)					
Marital status														
Married	4112 (87)	593 (13)	1			1		4131 (88)	574 (12)	1			1	
Unmarried	203 (99)	2 (1)	0.07	0.02, 0.27		0.07	0.02, 0.23	204 (99)	1 (<1)	0.04	0.01, 0.26		0.03	0.01, 0.24
Separated or divorced	78 (78)	22 (22)	1.96	1.20, 3.18		1.44	0.88, 2.33	71 (71)	29 (29)	2.94	1.90, 4.54		2.14	1.37, 3.35
Widowed	101 (90)	11 (10)	0.76	0.44, 1.29	0.001	0.79	0.45, 1.41	109 (97)	3 (3)	0.20	0.07, 0.59	<0.001	0.14	0.04, 0.44
All	4494 (88)	628 (12)						4515 (88)	607 (12)					
Parity														
No children	692 (92)	63 (8)	1			1		692 (92)	63 (8)	1			1	
1 or 2	2263 (86)	358 (14)	1.74	1.29, 2.34		1.41	1.04, 1.91	2279 (87)	342 (13)	1.65	1.28, 2.13		1.16	0.89, 1.51
3 or more	1539 (88)	207 (12)	1.48	1.09, 2.00	0.002	1.29	0.92, 1.80	1544 (88)	202 (12)	1.44	1.09, 1.90	0.002	0.96	0.70, 1.31
All	4494 (88)	628 (12)						4515 (88)	607 (12)					

For peer review only - http://bmjopen.bmj.com/site/about/guidelines.xhtml

Woman's schooling														
None	848 (89)	100 (11)	1			1		854 (90)	94 (10)	1			1	
1-5 years	734 (85)	125 (15)	1.44	1.03, 2.02		1.39	0.99, 1.96	737 (86)	122 (14)	1.50	1.13, 2.00		1.45	1.09, 1.94
6-10 years	1976 (87)	304 (13)	1.30	1.00, 1.70		1.24	0.93, 1.66	1983 (87)	297 (13)	1.36	1.07, 1.73		1.29	1.00, 1.67
11 years or more	936 (90)	99 (10)	0.90	0.66, 1.21	0.027	0.93	0.67, 1.29	941 (91)	94 (9)	0.91	0.72, 1.15	0.002	0.95	0.72, 1.24
All	4494 (88)	628 (12)						4515 (88)	607 (12)					
Woman's employment														
None	3437 (89)	433 (11)	1			1		3446 (89)	424 (11)	1			1	
Home-based	665 (86)	108 (14)	1.29	1.07, 1.56		1.22	1.01, 1.49	677 (88)	96 (12)	1.15	0.92, 1.44		1.08	0.85, 1.37
Outside the home	392 (82)	87 (18)	1.76	1.33, 2.32	<0.001	2.11	1.60, 2.80	392 (82)	87 (18)	1.80	1.31, 2.49	0.003	2.00	1.43, 2.80
All	4494 (88)	628 (12)						4515 (88)	607 (12)					

Table 2 continued

Religion														
Hindu	2685 (89)	317 (11)	1			1		2677 (89)	325 (11)	1			1	
Muslim	1629 (86)	273 (14)	1.42	1.15, 1.76		1.38	1.11, 1.72	1655 (87)	247 (13)	1.23	1.00, 1.52		1.23	1.00, 1.54
Buddhist	163 (83)	34 (17)	1.77	1.25, 2.50		1.68	1.16, 2.43	167 (85)	30 (15)	1.48	1.11, 1.97		1.33	1.00, 1.79
Other	17 (81)	4 (19)	1.99	0.64, 6.23	0.003	1.90	0.63, 5.74	16 (76)	5 (24)	2.57	0.90, 7.36	0.013	2.94	0.93, 9.27
All	4494 (88)	628 (12)						4515 (88)	607 (12)					
Caste														
General	2637 (88)	350 (12)	1			-	-	2651 (89)	336 (11)	1			-	-
Other Backward Caste	1064 (87)	157 (13)	1.11	0.92, 1.35		-	-	1063 (87)	158 (13)	1.17	0.95, 1.45		-	-
Scheduled Caste/Tribe	621 (86)	103 (14)	1.25	1.01, 1.54		-	-	629 (87)	95 (13)	1.19	0.93, 1.53		-	-
None stated	172 (91)	18 (9)	0.79	0.45, 1.37	0.202	-	-	172 (91)	18 (9)	0.83	0.47, 1.45	0.375	-	-
All	4494 (88)	628 (12)						4515 (88)	607 (12)					
Socioeconomic quintile (n=4918 ever-married women)														
1 poorest	829 (84)	161 (16)	1			1		849 (86)	141 (14)	1			-	-
2	861 (88)	117 (12)	0.70	0.55, 0.88		0.70	0.54, 0.90	865 (88)	113 (12)	0.79	0.58, 1.07		-	-

For peer review only

3	865 (88)	118 (12)	0.70	0.52, 0.95		0.72	0.53, 0.99	871 (89)	112 (11)	0.77	0.53, 1.13		-	-
4	876 (89)	108 (11)	0.63	0.49, 0.83		0.66	0.50, 0.88	876 (89)	108 (11)	0.74	0.53, 1.04		-	-
5 least poor	884 (90)	99 (10)	0.58	0.41, 0.81	0.007	0.63	0.45, 0.89	875 (89)	108 (11)	0.74	0.53, 1.04	0.325	-	-
All	4315 (88)	603 (12)						4336 (88)	582 (12)					
Husband's schooling (n=4805 women with living husbands)														
None	391 (81)	90 (19)	1			1		396 (82)	85 (18)	1			1	
1-5 years	507 (86)	83 (14)	0.71	0.51, 1.00		0.73	0.51, 1.04	509 (86)	81 (14)	0.74	0.50, 1.10		0.76	0.51, 1.11
6-10 years	2206 (87)	318 (13)	0.63	0.48, 0.82		0.66	0.50, 0.87	2214 (88)	310 (12)	0.65	0.48, 0.89		0.68	0.50, 0.92
11 years or more	1069 (89)	123 (11)	0.54	0.39, 0.75		0.63	0.41, 0.97	1066 (89)	126 (11)	0.59	0.43, 0.83		0.68	0.49, 0.95
Unknown	17 (91)	1 (9)	0.44	0.29, 0.66	<0.001	0.55	0.37, 0.84	17 (91)	1 (9)	0.48	0.33, 0.70	0.005	0.60	0.40, 0.92
All	4190 (87)	615 (13)						4202 (87)	603 (13)					
Husband employed in previous 12 months (n=4805 women with living husbands)														
No	73 (74)	26 (26)	1			1		76 (77)	23 (23)	1			1	
Yes	4111 (88)	586 (12)	0.40	0.25, 0.64		0.50	0.29, 0.84	4120 (88)	577 (12)	0.46	0.28, 0.76		0.75	0.45, 1.27
Unknown	6 (67)	3 (33)	1.40	0.35, 5.60	0.001	1.20	0.21, 6.91	6 (67)	3 (33)	1.65	0.32, 8.42	0.001	1.34	0.34, 5.27
All	4190 (87)	615 (13)						4202 (87)	603 (13)					
Husband uses alcohol/drugs (n=4805 women with living husbands)														
No	3198 (89)	377 (11)	1			1		3216 (90)	359 (10)	1			1	
Yes	992 (81)	238 (19)	2.04	1.59, 2.61	<0.001	1.81	1.36, 2.40	986 (80)	244 (20)	2.22	1.79, 2.75	<0.001	2.00	1.59, 2.53
All	4190 (87)	615 (13)						4202 (87)	603 (13)					

IPV: intimate partner violence. OR: odds ratio. AOR: adjusted odds ratio. Ref: reference category. ORs and 95% confidence intervals (CI) from univariable and multivariable logistic regression models. Multivariable models (Adjusted OR) include all variables for which Adjusted ORs are presented. Of 5122 women, we had data on socioeconomic status for the 4918 who had ever been married and data on their husbands for 4805 who had not been widowed.

For peer review only

For peer review only - http://bmjopen.bmj.com/site/about/guidelines.xhtml

Associations between disability and symptoms of anxiety and depression

Table 3 compares the profiles of women screened as having symptoms of at least moderate anxiety or depression with those who did not. 10% of women fell into this category and disabled women had nearly three times greater odds of doing so than non-disabled women (AOR 2.88, 95% CI 2.17, 3.82). Women who reported physical or sexual IPV (AOR 2.50, CI 1.77, 3.53) or emotional IPV (AOR 2.34, CI 1.59, 3.43) also had greater odds of anxiety or depression. Associations with anxiety or depression were also seen for separated, divorced, or widowed women, who were engaged in remunerated work, and whose husbands used alcohol or drugs. Associations were not seen in unadjusted models for parity or caste.

For peer review only

Table 3: Associations of positive screens for moderate or severe anxiety (GAD-7) or depression (PHQ-9) with disability and intimate partner violence (IPV) in the preceding year, for 5122 women aged 18-49 years living in informal settlements in Mumbai

Characteristic (n)	No, minimal, or mild anxiety or depression n (%)	Moderate or severe anxiety or depression n (%)	OR	(95% CI)	p	Adjusted OR	(95% CI)
Disability							
None	4217 (91)	400 (9)	1			1	
At least some impairment	372 (74)	133 (26)	3.77	2.99, 4.75	<0.001	2.88	2.17, 3.82
All	4589 (90)	533 (10)					
Physical or sexual IPV							

No	4132 (92)	362 (8)	1			1	
Yes	457 (73)	171 (27)	4.27	3.35, 5.45	<0.001	2.50	1.77, 3.53
All	4589 (90)	533 (10)					
Emotional IPV							
No	4150 (92)	365 (8)	1			1	
Yes	439 (72)	168 (28)	4.35	3.37, 5.62	<0.001	2.34	1.59, 3.43
All	4589 (90)	533 (10)					
Woman's age (years)							
<20	102 (89)	13 (11)	1			1	
20-29	1854 (91)	184 (9)	0.78	0.44, 1.38		0.69	0.38, 1.24
30-39	1857 (90)	199 (10)	0.84	0.47, 1.51		0.70	0.38, 1.28
>40	776 (85)	137 (15)	1.39	0.75, 2.56	<0.001	0.95	0.50, 1.82
All	4589 (90)	533 (10)					
Marital status							
Married	4267 (91)	438 (9)	1			1	
Unmarried	189 (92)	16 (8)	0.82	0.46, 1.48		1.21	0.64, 2.25
Separated or divorced	57 (57)	43 (43)	7.35	4.72, 11.45		4.73	2.68, 9.34
Widowed	76 (68)	36 (32)	4.61	2.91, 7.33	<0.001	3.25	1.72, 6.12
All	4589 (90)	533 (10)					
Woman's schooling							
None	819 (86)	129 (14)	1 =			1	
1-5 years	758 (88)	101 (12)	0.85	0.61, 1.17		0.72	0.50, 1.06
6-10 years	2046 (90)	234 (10)	0.73	0.56, 0.94		0.70	0.50, 0.96
11 years or more	966 (93)	69 (7)	0.45	0.32, 0.65	<0.001	0.59	0.39, 0.90
All	4589 (90)	533 (10)					
Woman's employment							
None	3535 (91)	335 (9)	1			1	
Home-based	662 (86)	111 (14)	1.77	1.39, 2.25		1.48	1.12, 1.96
Outside the home	392 (82)	87 (18)	2.34	1.73, 3.18	<0.001	1.70	1.19, 2.44
All	4589 (90)	533 (10)					
Religion							
Hindu	2737 (91)	265 (9)	1			1	
Muslim	1669 (88)	233 (12)	1.44	1.15, 1.81		1.28	0.98, 1.67
Buddhist	165 (84)	32 (16)	2.00	1.27, 3.17		1.63	0.98, 2.70
Other	18 (86)	3 (14)	1.72	0.45, 6.61	0.003	1.13	0.40, 3.22
All	4589 (90)	533 (10)					
Socioeconomic quintile (n=4918 ever-married women)							
1 poorest	857 (87)	133 (13)	1			1	
2	877 (90)	101 (10)	0.74	0.56, 0.98		0.73	0.53, 1.02
3	879 (89)	104 (11)	0.76	0.56, 1.04		0.77	0.54, 1.11
4	891 (91)	93 (9)	0.67	0.50, 0.90		0.68	0.48, 0.96
5 least poor	903 (92)	80 (8)	0.57	0.41, 0.80	0.014	0.64	0.43, 0.96
All	4407 (90)	511 (10)					
Husband's schooling (n=4805 women with living husbands)							

For peer review only

None	410 (85)	71 (15)	1			1	
1-5 years	517 (88)	73 (12)	0.82	0.59, 1.13		1.04	0.76, 1.44
6-10 years	2282 (90)	242 (10)	0.61	0.44, 0.84		1.01	0.73, 1.38
11 years or more	1103 (91)	89 (9)	0.58	0.38, 0.91		1.19	0.72, 1.95
Unknown	12 (94)	6 (6)	0.37	0.24, 0.58	<0.001	0.84	0.47, 1.50
All	4324 (90)	481 (10)					
Husband employed in previous 12 months (n=4805 women with living husbands)							
No	72 (73)	27 (27)	1			1	
Yes	4247 (90)	450 (10)	0.28	0.19, 0.42		0.81	0.45, 1.48
Unknown	5 (56)	4 (44)	2.13	0.49, 9.23	<0.001	1.70	0.29, 9.94
All	4324 (90)	481 (10)					
Husband uses alcohol or drugs (n=4805 women with living husbands)							
No	3317 (93)	258 (7)	1			1	
Yes	1007 (82)	223 (18)	2.85	2.19, 3.70	<0.001	1.98	1.48, 2.64
All	4324 (90)	481 (10)					

OR: odds ratio. AOR: adjusted odds ratio. ORs and 95% confidence intervals (CI) from univariable and multivariable logistic regression models.

Multivariable models (Adjusted OR) include all variables for which Adjusted ORs are presented. Of 5122 women, we had data on socioeconomic status for the 4918 who had ever been married, and data on their husbands for 4805 who had not been widowed.

Table 4 examines associations in more detail, presenting odds ratios for positive screens for moderate or severe anxiety, depression, and suicidal thinking in the presence of disability and physical, sexual, or emotional IPV. Overall, 6% of women screened positive for anxiety symptoms and 9% for symptoms of depression, while 13% had experienced suicidal thinking. In unadjusted models, disability increased the odds of each of these by two to four times. Adjusting for potential confounders reduced the odds ratios, but they remained substantial. IPV may also be a confounding factor, as it is associated with both mental health concerns and disability. Adjusting for IPV

For peer review only - http://bmjopen.bmj.com/site/about/guidelines.xhtml

and sociodemographic characteristics further reduced the odds ratios, but women with disability continued to have elevated odds of having a mental health concern. In this second adjusted model, women with functional difficulty had more than twice the odds of reporting anxiety symptoms as non-disabled women (AOR2 2.50, CI 1.78, 3.49) and nearly three times the odds of depression (AOR2 2.91, CI 2.13, 3.99). They had almost twice the odds of reporting suicidal thinking (AOR2 1.94, CI 1.50, 2.50). Independent of functional status, physical, sexual and emotional IPV were all associated with greater odds of having a mental health concern.

For peer review only

Table 4: Unadjusted and adjusted logistic regression models for associations between disability status, symptoms of anxiety and depression, and suicidal thinking, for 5122 women aged 18-49 years living in informal settlements in Mumbai

	Moderate or severe anxiety n (%)		OR	(95% CI)	Adjusted OR 1	(95% CI)	Adjusted OR 2	(95% CI)
	No	Yes						
Disability status								
None	4389 (95)	228 (5)	1		1		1	
At least some difficulty	425 (84)	80 (16)	3.62	2.77, 4.73	2.75	2.01, 3.76	2.50	1.78, 3.49
All	4814 (94)	308 (6)						
Physical or sexual IPV								
No	4293 (95)	201 (4)	1		1			
Yes	521 (83)	107 (17)	4.39	3.41, 5.65	4.05	2.98, 5.50	-	-
All	4814 (94)	308 (6)						
Emotional IPV								
No	4315 (96)	200 (4)	1		1			
Yes	499 (82)	108 (18)	4.67	3.59, 6.07	4.11	3.08, 5.49	-	-
All	4814 (94)	308 (6)						

	Moderate or severe depression n (%)		OR	(95% CI)	Adjusted OR 1	(95% CI)	Adjusted OR 2	(95% CI)
	No	Yes						
Disability status								
None	4280 (93)	337 (7)	1		1		1	
At least some difficulty	383 (76)	122 (24)	4.05	3.16, 5.18	3.12	2.36, 4.14	2.91	2.13, 3.99
All	4663 (91)	459 (9)						
Physical or sexual IPV								
No	4186 (93)	308 (7)	1		1			
Yes	477 (76)	151 (24)	4.30	3.40, 5.44	3.97	3.10, 5.09	-	-
All	4663 (91)	459 (9)						
Emotional IPV								
No	4204 (92)	311 (7)	1		1			
Yes	459 (76)	148 (24)	4.36	3.40, 5.58	4.01	3.04, 5.29	-	-
All	4663 (91)	459 (9)						
	Suicidal ideation n (%)		OR	(95% CI)	Adjusted OR 1	(95% CI)	Adjusted OR 2	(95% CI)
	No	Yes						
Disability status								
None	4089 (89)	528 (11)	1		1		1	
At least some difficulty	384 (76)	121 (24)	2.44	1.98, 3.01	2.13	1.66, 2.75	1.94	1.50, 2.50
All	4473 (87)	649 (13)						
Physical or sexual IPV								
No	4087 (91)	407 (9)	1		1			
Yes	386 (61)	242 (39)	6.30	5.01, 7.92	6.04	4.80, 7.62	-	-
All	4473 (87)	649 (13)						
Emotional IPV								
No	4112 (91)	403 (9)	1		1			
Yes	361 (59)	246 (41)	6.95	5.81, 8.33	6.55	5.36, 8.00	-	-
All	4473 (87)	649 (13)						

OR: odds ratio. AOR: adjusted odds ratio. ORs and 95% confidence intervals (CI) from univariable and multivariable logistic regression models.

Adjusted OR 1: adjusted for sociodemographic covariates: woman's age, marital status, schooling, employment, religion, socioeconomic quintile, and husband's schooling, employment, and alcohol or drug use.

Adjusted OR 2: adjusted for sociodemographic covariates and preceding year physical or sexual IPV and emotional IPV.

Discussion

For peer review only - http://bmjopen.bmj.com/site/about/guidelines.xhtml

In a cross-sectional survey of women living in informal settlements in Mumbai, disability was more common in women over 30 years of age and with children, who were separated, divorced or widowed, and from poorer households. Women with disability had over 50% greater odds of reporting physical, sexual, or emotional IPV than non-disabled women, and disabled women and those who had experienced IPV had greater odds of anxiety, depression, and suicidal thinking. Overall, the increased odds of disabled women experiencing IPV are consistent with studies from high-income [12] [85] and low-income settings [37] [86]. These findings contribute to our understanding of a serious but insufficiently investigated public health issue [15].

The reported prevalence of IPV among all women (12% for physical or sexual and emotional IPV) was lower than at national level [28] [29] and in slums [32] [33]. It was also lower among disabled women (17% for physical or sexual IPV, 16% for emotional IPV) compared with other studies [11, 35, 36]. The cause of this disparity is unclear. Strong efforts were made to help participants feel able to respond openly to questions, and the study used definitions of violence and survey instruments comparable with other studies: the lower rates may therefore be a fair reflection of levels of violence in

For peer review only - http://bmjopen.bmj.com/site/about/guidelines.xhtml

this community. Indeed, the rate accords closely with a study of physical (12%), sexual (2%), and emotional IPV (8%) experienced six weeks postpartum also undertaken in Mumbai informal settlements [74]. It is possible, nevertheless, that lower rates of violence may reflect under-reporting. Multiple factors play a role in the reporting of violence, such as a community's redressal structures, the extent of local welfare activities, and social norms. It may be that some communities are particularly close and not forthcoming enough to report violence. The context of each community is different and efforts must be made to understand local norms and behaviours in order to contextualise research outcomes.

The mental health burden in India is high and greater treatment provision is needed [87] [88]. One-in-seven Indian people are believed to be affected by some mental disorder and the burden of depressive and anxiety disorders is believed to be greatest among women [89]. For women with disabilities, the burden may be higher [90]. Two studies from South India found that disabled women were more likely to experience comorbid depression and diabetes than non-disabled women [91] [92]. One of these found that disabled women were nearly ten times more likely to have depression than

For peer review only

non-disabled women (AOR 9.5, CI, 2.2-40.8) [91]. Our study supports these findings: disabled women had more mental health concerns even when controlling for socioeconomic conditions and experience of violence. Whether poor mental health and disability precede violence or vice-versa cannot be determined here. Comorbid mental health disorders may also result from pre-existing health conditions and be exacerbated by exposure to violence. These issues should be the subject of future research. Further, as with the elevated vulnerability to violence, this burden of mental disorder needs to be communicated to relevant stakeholders and methods to address it developed.

The intersection of poverty, inequality, and discriminatory social norms is central to both violence and mental health disorders [93] [94]. In visualising and categorising interconnected determinants, the ecological framework used in our analysis has utility. Determining the source of violence, whether at individual micro or broader macro levels, is less clear-cut. The effects of multidimensional poverty and gender in ecologies of violence have received attention in recent years [9] [95], but more research is needed, both quantitative and qualitative, to understand the place of

For peer review only - http://bmjopen.bmj.com/site/about/guidelines.xhtml

disability and mental health in this matrix, particularly in the context of India. A potential data source is India's National Family Health Survey (NFHS), which has been broadened to include measures of disability [96]. The NFHS has already been used widely in IPV studies involving non-disabled women [97] [40]. It is hoped that this change will bring greater visibility to women with disabilities in investigations of violence.

Alongside improved visibility, regressive social and cultural norms must be challenged. These include the conception of disability as a personal failing or a sign of ill fate, and of disabled people as deserving of pity [98] [99], as well as patriarchal and stigmatizing attitudes towards women and mental disorder in general [100, 101], and tolerance of violence among both women and men [102]. This can be achieved, in part, through integrated inclusive interventions that incorporate anti-violence messages with other approaches, and through targeted interventions that seek to change violence-related norms directly [103] [55]. These efforts work best when addressing both men and women across all social classes and levels of the social ecology [65]. A recent targeted pilot study in Jharkhand, India that mobilised community resources through

For peer review only

For peer review only - http://bmjopen.bmj.com/site/about/guidelines.xhtml

participatory learning and action groups facilitated by Accredited Social Health Activists (ASHAs) reported reductions in experience and tolerance of violence and greater levels of help-seeking [104]. The study contributes to a growing body of evidence demonstrating that norms and practices that perpetuate inequities and violence can be challenged by community mobilisation and education [105]. Such interventions should work with disabled communities and activists, incorporating disability-positive messaging, to challenge discrimination at the community level and provide a more nuanced and inclusive approach to anti-violence efforts.

In healthcare settings, efforts should be made to identify and support disabled women unknown to healthcare and violence support services. Routine healthcare and IPV education and screening programmes need to be disability-inclusive and the vulnerability of disabled women needs to be incorporated into service provision. At screenings, disabled women should have access to safe and private spaces, away from accompanying family members who may be party to violence. Those delivering screening may need training to consider different kinds of functional difficulty and access needs such as adapted communication. Training should also incorporate

For peer review only - http://bmjopen.bmj.com/site/about/guidelines.xhtml

issues of trust and respect in order to challenge the stereotyping that can cause women with disabilities to be disbelieved when reporting episodes of violence [99] [106]. The need to challenge stereotypes and promote respect extends to intersecting issues such as widowhood, separation and divorce. Women can be stigmatised in India after the loss of a husband or the ending of a marriage, with a burden of fault placed on them [107]. In our study and elsewhere [10], women with disabilities were more likely to be separated or divorced, which is likely to further compound any stigma they already experience. Healthcare workers are well placed to challenge these kinds of stereotypes, but may themselves be party to sustaining them and require support to break reductive patterns of behaviour and thinking.

At a policy level, three major instruments in Indian law pertain directly to the evidence presented herein: the Protection of Women from Domestic Violence Act, 2005 (PWDVA) [108], the Rights of Persons with Disability Act, 2016 [109], and the Mental Healthcare Act, 2017 [110]. The greater scope for understanding and protection that these most recent instruments afford is a step in the right direction. The Rights of Persons with Disability Act brought India's disability policy in line with the United

For peer review only - http://bmjopen.bmj.com/site/about/guidelines.xhtml

Nations Convention on the Rights of Persons with Disabilities (UN-CRPD) and introduced a rights-based approach towards disability [111], while the Mental Healthcare Act shifted the approach to mental health away from criminalisation towards provision of healthcare [112]. However, there were limitations in the development of both [111] [113], including the Mental Healthcare Act's failure to sufficiently address stigma [114]. As for the PWDVA, India's persistently high rates of domestic violence, which have grown during the COVID-19 pandemic [115], are testament to the act's failure to effect any real change in society [116] [117]. This further demonstrates that, while policy can be an important tool for addressing attitudinal and environmental barriers, change will remain elusive without commitment to implementation supported by adequate funding and monitoring-accountability mechanisms.

Limitations

Limitations include the cross-sectional study design, which precludes determination of whether disability was an outcome of or risk factor for IPV. Use of self-report for exposure items is a further limitation, although the recall period was short, participants

For peer review only - http://bmjopen.bmj.com/site/about/guidelines.xhtml

being asked about their experiences in the last year. Responses to questions about IPV and mental health may have been self-censored due to the sensitive nature of the questions and therefore underestimated, despite researchers being familiar figures in the community and their efforts to develop trust. Use of self-report for disability is also a limitation and may be reflected in the low rates. The WG-SS determines a person's limitations based on self-perception in relation to their environment. Given the disadvantaged setting in which the participants lived, this may have also impacted the disability rates.

Conclusion

Violence against women in India is a major and ongoing public health problem. Women with disabilities are perhaps the most marginalised of all and experience violence to a greater degree than others. This violence derives from a culture that tolerates and perpetuates the marginalisation of both women in general and women with disabilities in particular and contributes to an unacceptable physical and mental health burden. The issue requires greater attention from national and community

For peer review only

leaders to address its causes, especially poverty and gender inequality, and to meet India’s commitments to its disabled population.

Acknowledgments

We are grateful to the women and community guardians who agreed to contribute to the study. We thank the investigators, data collection supervisors Miheeka Vast and Manju Singh, Bhaskar Kakad for supervision, Gauri Savkur for training support, Unnati Machchhar and Shilpa Adelkar for subsequent supervision of the intervention programme, Archana Bagra and Vibhavari Bali for financial and human resources management, and Vanessa D’Souza and Shanti Pantvaidya for leadership at SNEHA.

For peer review only

Contributorship

AR, ND and DO conceived the study. ND, MW, SK, AG, and DO developed the methodology for data collection. ND, SK, and AG oversaw investigation. AR and DO curated the data and designed the analysis. AR did the analysis and wrote the first draft. ND managed the project and ND and DO acquired funding. All authors critically reviewed drafts of the manuscript and read and commented on the final version.

Competing interests.

The authors declare no competing interest.

Funding

This research was funded in part by the Wellcome Trust (206417). For the purpose of open access, the author has applied a CC BY public copyright licence to any Author Accepted Manuscript version arising from this submission. The funder had no role in study design, data collection and analysis, decision to publish, or preparation of the manuscript.

Data sharing

Data are available online in Open Science Framework: Osrin, D. Data from: Intimate partner violence against women with disability and associated mental health

For peer review only

concerns: a cross-sectional survey in Mumbai, India. Open Science Framework. March 31, 2022. https://osf.io/zhtpw.

For peer review only

References

1. WHO. 2011. *World report on disability*. https://www.who.int/disabilities/world_report/2011/report/en/.
2. UN. 2006. *Convention on the Rights of Persons with Disabilities* (13 December 2006); A/RES/61/106, Annex I. https://www.un.org/development/desa/disabilities/convention-on-the-rights-of-persons-with-disabilities.html.
3. UNESCO. 2018. *Education and Disability: Analysis of Data from 49 Countries. Information Paper No. 49*. Institute for Statistics. http://uis.unesco.org/en/news/education-and-disability-analysis-data-49-countries.
4. Mitra, S., Posarac, A. and Vick, B. 2013. Disability and poverty in developing countries: a multidimensional study. *World Development*. 41; pp.1-18.
5. Heymann, J., Stein, M.A. and Moreno, G. 2013. *Disability and Equity at Work*. Oxford University Press, Oxford.

For peer review only - http://bmjopen.bmj.com/site/about/guidelines.xhtml

6. Mithen, J., Aitken, Z., Ziersch, A. and Kavanagh, A.M. 2015. Inequalities in social capital and health between people with and without disabilities. *Social Science & Medicine*. 126; pp.26-35.
7. Banks, L.M., Kuper, H. and Polack, S. 2017. Poverty and disability in low-and middle-income countries: A systematic review. *PloS One*. 12(12), p.e0189996.
8. Braithwaite, J., Mont, D. 2009. Disability and poverty: a survey of World Bank poverty assessments and implications. *Alter*. 3(3); pp.219-232.
9. Groce, N., Kett, M., Lang, R. and Trani, J.F. 2011. Disability and poverty: The need for a more nuanced understanding of implications for development policy and practice. *Third World Quarterly*. 32(8); pp.1493-1513.
10. Daruwalla, N., Chakravarty, S., Chatterji, S., Shah More, N., Alcock, G., Hawkes, S. and Osrin, D. 2013. Violence against women with disability in Mumbai, India: a qualitative study. *Sage Open*. 3(3); p.2158244013499144.
11. Rashid, S., Daruwalla, N., Puri, M., Hawkes, S. and Chow, J. 2012. *Count me in! Research Report: Violence Against Disabled, Lesbian, and Sex-Working Women in Bangladesh, India, and Nepal*. CREA. http://www.creaworld.org/sites/default/files/The%20Count%20Me%20In!%20Research%20Report.pdf.
12. Brownridge, D.A. 2006. Partner violence against women with disabilities: Prevalence, risk and explanations. *Violence Against Women*. 12; pp.805-822.
13. Krug, E.G., Mercy, J.A., Dahlberg, L.L. and Zwi, A.B. (eds.). 2002. *World Report on Violence and Health*. World Health Organisation. https://www.who.int/violence_injury_prevention/violence/world_report/en/.
14. Butchart, A., Mikton, C. 2014. Global Status Report on Violence Prevention. *World Health Organisation*. https://www.who.int/violence_injury_prevention/violence/status_report/2014/en/.
15. Hughes, K., Bellis, M.A., Jones, L., Wood, S., Bates, G., Eckley, L., McCoy, E., Mikton, C., Shakespeare, T. and Officer, A. 2012. Prevalence and risk of violence against adults with disabilities: a systematic review and meta-analysis of observational studies. *The Lancet*. 379(9826); pp.1621-1629.
16. García-Moreno, C., Pallitto, C., Devries, K., Stöckl, H., Watts, C. and Abrahams, N. 2013. Global and regional estimates of violence against women: prevalence and health effects of intimate partner violence and non-partner sexual violence. *World Health Organization*. https://www.who.int/reproductivehealth/publications/violence/9789241564625/en/.
17. Cieza, A., Sabariego, C., Bickenbach, J. and Chatterji, S. 2018. Rethinking disability. *BMC Medicine*. 16(1); pp.1-5.
18. Mikton, C., Maguire, H. and Shakespeare, T. 2014. A systematic review of the effectiveness of interventions to prevent and respond to violence against persons with disabilities. *Journal of Interpersonal Violence*. 29(17); pp.3207-3226.
19. Government of India. 2016a. *Disabled Persons in India: A statistical profile 2016*. Ministry of Statistics and Programme Implementation. http://mospi.nic.in/sites/default/files/publication_reports/Disabled_persons_in_India_2016.pdf.
20. Government of India. 2018. *Persons with Disabilities in India*. National Statistics Office. http://www.mospi.gov.in/sites/default/files/publication_reports/Report_583_Final_0.pdf.

For peer review only

21. Dandona, R., Pandey, A., George, S., Kumar, G.A. and Dandona, L. 2019. India's disability estimates: Limitations and way forward. *PloS One*. 14(9).
22. O'Keefe, P. 2009. *People with Disabilities in India: From Commitments to Outcomes*. World Bank Human Development Unit, South Asia Region. https://documents.worldbank.org/en/publication/documents-reports/documentdetail/577801468259486686/people-with-disabilities-in-india-from-commitments-to-outcomes.
23. Mitra, S. and Sambamoorthi, U. 2014. Disability prevalence among adults: estimates for 54 countries and progress toward a global estimate. *Disability and Rehabilitation*. 36(11); pp.940-947.
24. Velayutham, B., Kangusamy, B. and Mehendale, S. 2017. Prevalence of Disability in Tamil Nadu, India. *The National Medical Journal of India*. 30(3); p.125.
25. Mopari, R., Garg, B., Puliyel, J. and Varughese, S. 2019. Measuring disability in an urban slum community in India using the Washington Group questionnaire. *Disability and Health Journal*. 12(2); pp.263-268.
26. Gudlavalleti, M.V.S. 2014. *The Telangana Disability Study, India Country Report*. International Centre for Evidence in Disability and London School of Hygiene and Tropical Medicine. https://www.researchgate.net/publication/280312727_Telangana_Disability_Study.
27. Saikia, N., Bora, J.K., Jasilionis, D. and Shkolnikov, V.M. 2016. Disability divides in India: evidence from the 2011 census. *PloS One*. 11(8).
28. IIPS. 2017. *National Family Health Survey 2015-16 (NFHS-4)*. International Institute for Population Sciences, Government of India, Mumbai. https://dhsprogram.com/pubs/pdf/FR339/FR339.pdf.
29. Kalokhe, A., del Rio, C., Dunkle, K., Stephenson, R., Metheny, N., Paranjape, A. and Sahay, S. 2017. Domestic violence against women in India: A systematic review of a decade of quantitative studies. *Global Public Health*. 12(4); pp.498-513.
30. Begum, S., Donta, B., Nair, S. and Prakasam, C.P. 2015. Socio-demographic factors associated with domestic violence in urban slums, Mumbai, Maharashtra, India. *The Indian Journal of Medical Research*. 141(6); p.783.
31. Dasgupta, A., Preeti, P.S., Sahoo, S.K., Biswas, D., Kumar, A. and Das, M. 2015. Domestic Violence and its Determinants: A cross-sectional study among women in a slum of Kolkata. *Indian Journal of Community Health*. 27(3); pp.334-340.
32. Gaikwad, V., Rao, D.H. 2014. A Cross-sectional Study of Domestic Violence Perpetrated by Intimate Partner against Married Women in the Reproductive Age Group in an Urban Slum Area in Mumbai. *Indian Journal of Public Health Research & Development*. 5(1); pp.49-54.
33. Pal, J., Mullick, T.H., Ahmad, S. and Yadav, A.K. 2017. Domestic Violence against Women-An Unsolved Issue: A Community Based Study in an Urban Slum of Kolkata, India. *Journal of Clinical & Diagnostic Research*. 11(10).
34. Sinha, A., Mallik, S., Sanyal, D., Dasgupta, S., Pal, D. and Mukherjee, A. 2012. Domestic violence among ever married women of reproductive age group in a slum area of Kolkata. *Indian Journal of Public Health Research & Development*. 56(1); p.31.
35. Deepak, S., Kumar, J., Santosh, B., Gornalli, S., Manikappa, P., Vyjantha, U. and Giriyappa, R. 2014. Violence against persons with disabilities in Bidar District, India. *Disability, CBR & Inclusive Development*. 25(2); pp.35-53.
36. Mohapatra, S. and Mohanty, M. 2005. *Abuse and activity limitation - A study on domestic violence against disabled women in Orissa, India*. Oxfam, India.

For peer review only - http://bmjopen.bmj.com/site/about/guidelines.xhtml

37. Gupta, J., Cardoso, L.F., Ferguson, G., Shrestha, B., Shrestha, P.N., Harris, C., Groce, N. and Clark, C.J. 2018. Disability status, intimate partner violence and perceived social support among married women in three districts of the Terai region of Nepal. *BMJ Global Health*. 3(5); p.e000934.
38. Hasan, T., Muhaddes, T., Camellia, S., Selim, N. and Rashid, S.F. 2014. Prevalence and experiences of intimate partner violence against women with disabilities in Bangladesh: Results of an explanatory sequential mixed-method study. *Journal of Interpersonal Violence*. 29(17); pp.3105-3126.
39. Puri, M., Misra, G. and Hawkes, S. 2015. Hidden voices: prevalence and risk factors for violence against women with disabilities in Nepal. *BMC Public Health*. 15(1); p.261.
40. Sabri, B., Renner, L.M., Stockman, J.K., Mittal, M. and Decker, M.R. 2014. Risk factors for severe intimate partner violence and violence-related injuries among women in India. *Women & Health*. 54(4); pp.281-300.
41. Spiwak, R., Logsetty, S., Afifi, T.O. and Sareen, J. 2015. Severe partner perpetrated burn: Examining a nationally representative sample of women in India. *Burns*. 41(8); pp.1847-1854.
42. Silverman, J.G., Decker, M.R., Saggurti, N., Balaiah, D. and Raj, A. 2008. Intimate partner violence and HIV infection among married Indian women. *JAMA*. 300(6); pp.703-710.
43. Sudha, S. and Morrison, S. 2011. Marital violence and women's reproductive health care in Uttar Pradesh, India. *Women's Health Issues*. 21(3); pp.214-221.
44. Anand, E., Unisa, S. and Singh, J. 2017. Intimate partner violence and unintended pregnancy among adolescent and young adult married women in south Asia. *Journal of Biosocial Science*. 49(2); pp.206-21.
45. Dhar, D., McDougal, L., Hay, K., Atmavilas, Y., Silverman, J., Triplett, D. and Raj, A. 2018. Associations between intimate partner violence and reproductive and maternal health outcomes in Bihar, India: a cross-sectional study. *Reproductive Health*. 15(1); p.109.
46. Jejeebhoy, S.J., Santhya, K.G. and Acharya, R. 2013. Physical and sexual violence and symptoms of gynaecological morbidity among married young women in India. *Global Public Health*. 8(10); pp.1151-1167.
47. Chandra, P.S., Satyanarayana, V.A. and Carey, M.P. 2009. Women reporting intimate partner violence in India: Associations with PTSD and depressive symptoms. *Archives of Women's Mental Health*. 12(4); p.203.
48. Chowdhury, A.N., Brahma, A., Banerjee, S. and Biswas, M.K. 2009. Pattern of domestic violence amongst non-fatal deliberate self-harm attempters: A study from primary care of West Bengal. *Indian Journal of Psychiatry*. 51(2); p.96.
49. Maselko, J. and Patel, V. 2008. Why women attempt suicide: the role of mental illness and social disadvantage in a community cohort study in India. *Journal of Epidemiology & Community Health*. 62(9); pp.817-822.
50. Suryanarayana, M., Agrawal, A. and Prabhu, K.S. 2011. *Inequality adjusted human development index for India's states*. United Nations Development Programme, India.
51. MCGM. 2020. *Mumbai Census Population*. Municipal Corporation of Greater Mumbai. https://portal.mcgm.gov.in/irj/portal/anonymous/qlvitalstatsreport?guest_user=english.
52. Chandramouli, C. 2011. *Housing stock, amenities and assets in slums - Census 2011*. New Delhi: Office of the Registrar General and Census Commissioner.

For peer review only - http://bmjopen.bmj.com/site/about/guidelines.xhtml

53. Ezeh, A., Oyebode, O., Satterthwaite, D., Chen, Y.F., Ndugwa, R., Sartori, J., Mberu, B., Melendez-Torres, G.J., Haregu, T., Watson, S.I. and Caiaffa, W. 2017. The history, geography, and sociology of slums and the health problems of people who live in slums. *The Lancet*. 389(10068); pp.547-558.
54. UN-Habitat. 2017. *Human Rights in Cities Handbook Series Volume 1: The Human Rights-Based Approach to Housing and Slum Upgrading*. https://www.ohchr.org/Documents/Issues/Housing/InformalSettlements/UNHABITAT_HumanRights-BasedApproch.pdf.
55. Daruwalla, N., Machchhar, U., Pantvaidya, S., D'Souza, V., Gram, L., Copas, A. and Osrin, D. 2019. Community interventions to prevent violence against women and girls in informal settlements in Mumbai: the SNEHA-TARA pragmatic cluster randomised controlled trial. *Trials*. 20(1); p.743.
56. Daruwalla, N., Kanougiya, S., Gupta, A., Gram, L. and Osrin, D. . 2020. Prevalence of domestic violence against women in informal settlements in Mumbai, India: a cross-sectional survey. *BMJ Open*. 10:e042444. DOI: 10.1136/bmjopen-2020-042444.
57. Kalokhe, A.S., Stephenson, R., Kelley, M.E., Dunkle, K.L., Paranjape, A., Solas, V., Karve, L., del Rio, C. and Sahay, S. 2016. The development and validation of the Indian family violence and control scale. *PLoS One*. 11(1); p.e0148120.
58. Johnson, H., Ollus, N. and Nevala, S. 2008. *Violence Against Women: An International Perspective*. Springer Science & Business Media, New York.
59. García-Moreno, C., Jansen, H.A., Ellsberg, M., Heise, L. and Watts, C. 2005. WHO multi-country study on women's health and domestic violence against women. *World Health Organization*. https://www.who.int/reproductivehealth/publications/violence/24159358X/en/.
60. Washington Group on Disability Statistics. 2009. *Understanding and interpreting disability as measured using the WG short set of questions*. https://www.cdc.gov/nchs/data/washington_group/meeting8/interpreting_disability.pdf.
61. Spitzer, R.L., Kroenke, K., Williams, J.B. and Löwe, B. 2006. A brief measure for assessing generalized anxiety disorder: the GAD-7. *Archives of Internal Medicine*. 166(10); pp.1092-1097.
62. Kroenke, K., Spitzer, R.L. and Williams, J.B. 2001. The PHQ-9: validity of a brief depression severity measure. *Journal of General Internal Medicine*. 16(9); pp.606-613.
63. Kroenke, K. and Spitzer, R.L. 2010. *Instruction manual: Instructions for patient health questionnaire (PHQ) and GAD-7 measures*. https://www.phqscreeners.com/.
64. Heise, L. 1998. Violence against women: An integrated, ecological framework. *Violence Against Women*. 4(3); pp.262-290.
65. Heise, L. 2011. *What works to prevent partner violence? An evidence overview*. STRIVE. http://strive.lshtm.ac.uk/system/files/attachments/What%20works%20to%20prevent%20partner%20violence.pdf.
66. Jain, S., Varshney, K., Vaid, N.B., Guleria, K., Vaid, K. and Sharma, N. 2017. A hospital-based study of intimate partner violence during pregnancy. *International Journal of Gynecology & Obstetrics*. 137(1); pp.8-13.
67. Mahapatro, M., Gupta, R.N. and Gupta, V. 2012. The risk factor of domestic violence in India. *Indian journal of Community Medicine: Official Publication of Indian Association of Preventive & Social Medicine*. 37(3); p.153.
68. Shrivastava, P.S. and Shrivastava, S.R. 2013. A study of spousal domestic violence in an urban slum of Mumbai. *International Journal of Preventive Medicine*. 4(1); p.27.

For peer review only - http://bmjopen.bmj.com/site/about/guidelines.xhtml

69. Biswas, C.S. 2017. Spousal Violence against Working Women in India. *Journal of Family Violence*. 32(1); pp.55-67.
70. Bhattacharya, H. 2015. Spousal violence and Women's employment in India. *Feminist Economics*. 21(2); pp.30-52.
71. Weitzman, A. 2014. Women's and men's relative status and intimate partner violence in India. *Population and Development Review*. 40(1); pp.55-75.
72. Rapp, D., Zoch, B., Khan, M.M.H., Pollmann, T. and Krämer, A. 2012. Association between gap in spousal education and domestic violence in India and Bangladesh. *BMC Public Health*. 12(1); p.467.
73. Kimuna, S.R., Djamba, Y.K., Ciciurkaite, G. and Cherukuri, S. 2013. Domestic violence in India: Insights from the 2005-2006 national family health survey. *Journal of Interpersonal Violence*. 28(4); pp.773-807.
74. Das, S., Bapat, U., More, N.S., Alcock, G., Joshi, W., Pantvaidya, S. and Osrin, D. 2013. Intimate partner violence against women during and after pregnancy: a cross-sectional study in Mumbai slums. *BMC Public Health*. 13(1); p.817.
75. Wagman, J.A., Donta, B., Ritter, J., Naik, D.D., Nair, S., Saggurti, N., Raj, A. and Silverman, J.G. 2018. Husband's alcohol use, intimate partner violence, and family maltreatment of low-income postpartum women in Mumbai, India. *Journal of Interpersonal Violence*. 33(14); pp.2241-2267.
76. Huria, K.A., Deepti, D., Lajya, D. and Sunder, S.S. 2005. Domestic violence in pregnancy in North Indian women. *Indian Journal of Medical Sciences*. 59(5); pp.195-199.
77. Chibber, K.S., Krupp, K., Padian, N. and Madhivanan, P. 2012. Examining the determinants of sexual violence among young, married women in Southern India. *Journal of Interpersonal Violence*. 27(12); pp.2465-2483.
78. Dalal, K., Lindqvist, K. 2012. A national study of the prevalence and correlates of domestic violence among women in India. *Asia Pacific Journal of Public Health*. 24(2); pp.265-277.
79. Krishnan, S. 2005. Gender, caste, and economic inequalities and marital violence in rural South India. *Health Care for Women International*. 26(1); pp.87-99.
80. Victora, C.G., Huttly, S.R., Fuchs, S.C. and Olinto, M.T. 1997. The role of conceptual frameworks in epidemiological analysis: a hierarchical approach. *International Journal of Epidemiology*. 26(1); pp.224-227.
81. Filmer, D., Pritchett, L.H. 2001. Estimating wealth effects without expenditure data—or tears: an application to educational enrollments in states of India. *Demography*. 38(1); pp.115-132.
82. Vyas, S. and Kumaranayake, L. 2006. Constructing socio-economic status indices: how to use principal components analysis. *Health Policy and Planning*. 21(6); pp.459-468.
[dataset] 83. Osrin, D. Data from: Intimate partner violence against women with disability and associated mental health concerns: a cross-sectional survey in Mumbai, India. Open Science Framework. March 31, 2022. https://osf.io/zhtpw.
84. WHO. 2001. *Putting Women First: Ethical and Safety Recommendations for Research on Domestic Violence against Women*. Department of Gender and Women's Health, Family and Community Health. https://www.who.int/gender-equity-rights/knowledge/who_fch_gwh_01.1/en/.
85. Hahn, J.W., McCormick, M.C., Silverman, J.G., Robinson, E.B. and Koenen, K.C. 2014. Examining the impact of disability status on intimate partner violence victimization in a population sample. *Journal of Interpersonal Violence*. 29(17); pp.3063-3085.

For peer review only - http://bmjopen.bmj.com/site/about/guidelines.xhtml

86. Van Der Heijden, I. and Dunkle, K. 2017. *What Works Evidence Review: Preventing violence against women and girls with disabilities in lower-and middle-income countries (LMICs)*. UKAIDS. https://www.whatworks.co.za/resources/evidence-reviews/item/349-what-works-evidence-review-preventing-violence-against-women-and-girls-with-disabilities-in-lower-and-middle-income-countries-lmics.
87. Kallakuri, S., Devarapalli, S., Tripathi, A.P., Patel, A. and Maulik, P.K. 2018. Common mental disorders and risk factors in rural India: baseline data from the SMART mental health project. *BJPsych Open*. 4(4); pp.192-198.
88. Kar, S.K., Sharma, E., Agarwal, V., Singh, S.K., Dalal, P.K., Singh, A., Gopalkrishna, G. and Rao, G.N. 2018. Prevalence and pattern of mental illnesses in Uttar Pradesh, India: Findings from the National Mental Health Survey 2015–16. *Asian Journal of Psychiatry*. 38; pp.45-52.
89. Sagar, R., Dandona, R., Gururaj, G., Dhaliwal, R.S., Singh, A., Ferrari, A., Dua, T., Ganguli, A., Varghese, M., Chakma, J.K. and Kumar, G.A. 2020. The burden of mental disorders across the states of India: The Global Burden of Disease Study 1990–2017. *The Lancet Psychiatry*. 7(2); pp.148-161.
90. Dembo, R.S., Mitra, M. and McKee, M. 2018. The psychological consequences of violence against people with disabilities. *Disability and Health Journal*. 11(3); pp.390-397.
91. Gudlavalleti, M.V.S., John, N., Allagh, K., Sagar, J., Kamalakannan, S., Ramachandra, S.S 2014. Access to health care and employment status of people with disabilities in South India, the SIDE (South India Disability Evidence) study. *BMC Public Health*. 14(1); p.1125.
92. Murthy, G.V.S., John, N., Sagar, J. . 2014. Reproductive health of women with and without disabilities in South India, the SIDE study (South India Disability Evidence) study: a case control study. *BMC Women's Health*. 14(1); p.146.
93. Bhattacharya, A., Camacho, D., Kimberly, L.L. and Lukens, E.P. 2019. Women’s experiences and perceptions of depression in India: A metaethnography. *Qualitative Health Research*. 29(1); pp.80-95.
94. Malhotra, S. and Shah, R. 2015. Women and mental health in India: An overview. *Indian Journal of Psychiatry*. 57(Suppl 2); p.S205.
95. Subbaraman, R., Nolan, L., Shitole, T., Sawant, K., Shitole, S., Sood, K., Nanarkar, M., Ghannam, J., Betancourt, T.S., Bloom, D.E. and Patil-Deshmukh, A. 2014. The psychological toll of slum living in Mumbai, India: a mixed methods study. *Social Science & Medicine*. 119; pp.155-169.
96. Government of India. 2019. *Conducting the NFHS/Annual Health Surveys*. Ministry of Health and Family Welfare. https://pib.gov.in/PressReleaseIframePage.aspx?PRID=1576629.
97. Ahmad, J., Khan, N. and Mozumdar, A. 2019. Spousal Violence Against Women in India: A Social–Ecological Analysis Using Data from the National Family Health Survey 2015 to 2016. *Journal of Interpersonal Violence*. DOI: 10.1177/0886260519881530.
98. Ghai, A. 2019. Rethinking Disability in India. *Taylor & Francis*. London.
99. Ghosh, N. 2016. Negotiating Femininity: Lived Experiences of Women with Locomotor Disabilities in Bengal. In: Ghosh, N. (ed.). *Interrogating Disability in India*. 2016 pp. 127-144, Springer, New Delhi.
100. Goel, R. 2005. Sita’s trousseau: Restorative justice, domestic violence, and South Asian culture. *Violence Against Women*. 11; 639-665.

For peer review only - http://bmjopen.bmj.com/site/about/guidelines.xhtml

101. Ahmed-Ghosh, H. 2004. Chattels of society: Domestic violence in India. *Violence against women*. 10; 94-118.
102. Rowan, K., Mumford, E. and Clark, C.J. . 2018. Is women's empowerment associated with help-seeking for spousal violence in India? *Journal of interpersonal violence*. 33; 1519-1548.
103. WHO. 2009. *Changing cultural and social norms supportive of violence behaviour. Series of briefings on violence prevention: the evidence*. https://www.who.int/violence_injury_prevention/violence/norms.pdf.
104. Nair, N., Daruwalla, N., Osrin, D., Rath, S., Gagrai, S., Sahu, R., Pradhan, H., De, M., Ambavkar, G., Das, N. and Dungdung, G.P. . 2020. Community mobilisation to prevent violence against women and girls in eastern India through participatory learning and action with women's groups facilitated by accredited social health activists: a before-and-after pilot study. *BMC International Health and Human Rights*. 20(1), pp.1-12.
105. Ellsberg, M., Arango, D.J., Morton, M., Gennari, F., Kiplesund, S., Contreras, M. and Watts, C. . 2015. Prevention of violence against women and girls: what does the evidence say? *The Lancet*. 385(9977), pp.1555-1566.
106. Sharma, S. and Sivakami, M. 2019. Sexual and reproductive health concerns of persons with disability in india: An issue of deep-rooted silence. *Journal of Biosocial Science*. 51(2); pp.225-243.
107. Asthana, A. 2017. Women in Distress: A Study on the Widowed, Divorced and Separated Women in Surguja (Chhattisgarh, India). *Divorced and Separated Women in Surguja (Chhattisgarh, India)*. https://dx.doi.org/10.2139/ssrn.3223334.
108. Government of India. 2005. *The Protection of Women from Domestic Violence Act, 2005*. Ministry of Law and Justice. https://legislative.gov.in/actsofparliamentfromtheyear/protection-women-domestic-violence-act-2005.
109. Government of India. 2016b. *The Rights of Persons with Disability Act, 2016*. Ministry of Law and Justice. http://legislative.gov.in/actsofparliamentfromtheyear/rights-persons-disabilities-act-2016.
110. Government of India. 2017. *The Mental Healthcare Act, 2017*. Ministry of Law and Justice. https://www.prsindia.org/uploads/media/Mental%20Health/Mental%20Healthcare%20Act,%202017.pdf.
111. Math, S.B., Gowda, G.S., Basavaraju, V., Manjunatha, N., Kumar, C.N., Philip, S. and Gowda, M. 2019. The rights of persons with disability act, 2016: Challenges and opportunities. *Indian Journal of Psychiatry*. 61(Suppl 4); p.S809.
112. Mishra, A. and Galhotra, A. 2018. Mental healthcare Act 2017: Need to wait and watch. *International Journal of Applied and Basic Medical Research*. 8(2), p.67.
113. Balakrishnan, A., Kulkarni, K., Moirangthem, S., Kumar, C.N., Math, S.B. and Murthy, P. 2019. The rights of persons with disabilities Act 2016: Mental health implications. *Indian Journal of Psychological Medicine*. 41(2), pp.119-125.
114. Duffy, R.M., Gulati, G., Kasar, N., Paralikar, V., Narayan, C.L., Desousa, A., Goyal, N. and Kelly, B.D. 2019. Stigma, inclusion and India's Mental Healthcare Act 2017. *Journal of Public Mental Health*. Vol. 18 No. 3, pp. 199-205.
115. Maji, S., Bansod, S. and Singh, T. 2021. Domestic violence during COVID-19 pandemic: The case for Indian women. *Journal of Community & Applied Social Psychology*. https://doi.org/10.1002/casp.2501.

For peer review only

116. Manjoo, R. 2014. Report of the Special Rapporteur on violence against women, its causes and consequences. *United Nations Human Rights Council*. Twenty-sixth session. https://www.ohchr.org/en/hrbodies/hrc/regularsessions/session26/documents/a-hrc-26-38-add1_en.doc.

117. Tulsyan, A. 2016. Protection of Women from Domestic Violence Act 2005: Lessons from a Decade of Implementation. *Oxfam India*. https://www.oxfamindia.org/sites/default/files/Protection-of-women-from-domestic-violence-act-2005.pdf.

Footnote reference

- Shakespeare, T., 2006. The social model of disability. *The Disability Studies Reader*, *2*, pp.197-204.

For peer review only

Text (English)	Type	Text (Hindi)
**What is your religion?**		**आपका धर्म क्या है?**
Hindu	Choice	हिंदू
Muslim	Choice	मुस्लिम
Christian	Choice	ईसाई
Sikh	Choice	सिख
Buddhist/Neo-Buddhist	Choice	बौद्ध / नव-बौद्ध
Jain	Choice	जैन
Parsi/Zoroastrian	Choice	पारसी / पारसी
No religion/did not say	Choice	कोई धर्म नहीं/जानकारी नहीं
**What is your caste?**		**आपकी जाति क्या हैं?**
Open/General	Choice	सामान्य
OBC	Choice	ओबीसी
Scheduled caste (SC)	Choice	अनुसूचित जाति
Scheduled tribe (ST)	Choice	अनुसूचित जनजाति
None of these	Choice	इनमें से कोई भी नहीं
**What kind of house do you live in?**		**आप किस तरह के घर में रहतें हैं?**
Kachha (non-concrete)	Choice	कच्चा
Pucca (concrete)	Choice	पक्का
Mixed concrete and non-concrete	Choice	कच्चा एवं पक्का, दोनों
Other	Choice	अन्य
**own?**		आपका अपना घर है?**
Own	Choice	स्वयं का
Rented	Choice	किराये का
Other	Choice	अन्य
**What type of toilet facility do you use?**		**आप किस प्रकार के शौचालय सुविधा का प्रयोग करते हैं?**
Private or individual	Choice	निजी
Public	Choice	सार्वजानिक
Paid toilet	Choice	निजी लेकिन भुगतान शौचालय
Charity toilet	Choice	सार्वजानिक लेकिन भुगतान सौचालय

For peer review only

Open defecation	Choice	खुले में
Other	Choice	अन्य
Now I would like to ask you about some important aspects of a woman's life. You may find some of the questions personal. However, your answers are crucial for helping us understand the condition of women in the community. Let me assure you that your answers will be completely confidential and no one else in your household will know that you were asked. If I ask you any question you don't want to answer, just let me know and I will go on to the next question.		***अब मैं आपसे महिलाओं के जीवन के कुछ महत्वपूर्ण पहलुओं के बारे में सवाल पूछना चाहती हूँ. इनमें से कुछ प्रश्न व्यक्तिगत हो सकते हैं. इन जानकारी से बस्ती में महिलाओं की स्थिति को और अच्छे से समझने में के लिए आपके उत्तर महत्वपूर्ण हैं. मैं आपको भरोसा देती हूँ कि आपके उत्तर पूरी तरह से गोपनीय रखे जायेंगे और उन्हें किसी को भी बताया नहीं जाएगा, और आपके परिवार में से भी कोई भी नहीं जान पायेगा कि आपसे ये सरे सवाल पूछे गए हैं. यदि आप मेरे द्वारा पूछे गये किसी भी सवाल का जवाब नहीं चाहतीं हैं कृपया मुझे बताये, मै आगे के सवालों पर बढ़ जाउंगी.***
**What is your age?**	Integer	**आपकी (उत्तरदाता) की उम्र कितनी उम्र है?**
**What is your current marital status?**		**वर्तमान में आपकी (उत्तरदाता) वैवाहिक स्थिति क्या है?**
Unmarried	Choice	अविवाहित
Married, Gauna not performed	Choice	शादी की पर गौना नहीं हुआ
Currently married	Choice	शादीशुदा
Living with partner	Choice	साथी के साथ रहना
Separated	Choice	पति से अलग
Divorced	Choice	तलाकशुदा
Widowed/Widower	Choice	विधवा/ विधुर
Answer refused	Choice	उत्तर से इनकार कर दिया
**completed?****		**आपकी उच्चतम शिक्षा क्या है?**
No formal education	Choice	कोई औपचारिक शिक्षा नहीं
Primary (1-5th standard)	Choice	प्राइमरी (1-5th standard)
Middle (6-8th standard)	Choice	मिडिल (6-8th standard)
High school (9-10th standard)	Choice	हाई स्कूल (9-10th standard)
Senior school (11-12th standard)	Choice	सीनियर स्कूल (11-12th standard)
Undergraduate	Choice	ग्रेजुएशन पूरा नहीं
Graduate	Choice	ग्रेजुएशन

For peer review only

Incomplete postgraduate	Choice	पोस्ट ग्रेजुएशन पूरा नहीं
Postgraduate or higher	Choice	पोस्ट ग्रेजुएशन और आगे
Other	Choice	अन्य
**WG-SS**		**विकलांगता**
difficulty. Please reply which describes your condition appropriately.***		प्रकार की कठिनाई है तो, कृपया उत्तर दें जो कि आपकी स्थिति का उचित रूप से वर्णन करता हो.***
**Do you have difficulty seeing, even if wearing glasses?**		*(कृपया पूंछे कि क्या वे चश्मा पहनने पहनते है? यदि हाँ तो क्या चश्मा पहनने के बाद भी उन्हें देखने में परेशानी होती है क्या?)*
No - no difficulty	Choice	नहीं देखने में कोई कठिनाई नहीं
Yes – some difficulty	Choice	हां - देखने में कुछ कठिनाई
Yes – a lot of difficulty	Choice	हां - देखने में बहुत मुश्किल
Cannot do at all	Choice	बिल्कुल नहीं देख सकते
hearing aid?**		उपयोग करते है)?**
**Do you have difficulty walking or climbing steps?**		**क्या आपको चलने या चढ़ने में कठिनाई होती है?**
concentrating?**		**क्या आपको चीजे याद रखने में या ध्यान केंद्रित करने में कठिनाई होती है?**
washing all over or dressing?**		है?**
difficulty communicating, for example, understanding or being understood?**		कठिनाई होती है, उदाहरण के लिए, दूसरों की बातों को समझ पाने में या खुद की बातों को दूसरों को समझाने में?**
**Respondent's livelihood**		**आजीविका**
they are paid in cash or kind. Others sell things, have a small business or work on the family farm or in the family business. In the last 12 months, have you done any of these things or any other work?***		नगद या किसी चीज के रूप में भुगतान दिया जाता है, अन्य महिलायें सामान बेचती हैं, छोटा व्यापार करती हैं, अथवा घर की खेती या घर के व्यापार में हाथ बंटाती हैं. पिछले 12 महीनों में, क्या आपने इनमें से कोई काम या कोई और काम किया है?***
No	Choice	नहीं
Yes	Choice	हाँ
**What kind of work do you mainly do?**		**मुख्यतः आप किस प्रकार का काम करती हों?**
**Home-based earnings** *[stitching, pani-puri making, embroidery, snacks, gota/sitara]*	Choice	**घर पर आधारित काम (आय)** *[सहायता [सिलाई, पानी-पुरी बनाने, कढ़ाई, नाश्ते बनाने, गोटा / सीतारा टकिंग]*
**Vendor job** *[fish selling, vegetable selling, flower/garland selling, snacks selling]*	Choice	**विक्रेता नौकरी*** [मछली बेचने, सब्जी की बिक्री, फूल / माला बेचने, नाश्ते की बिक्री]*

For peer review only

Shop, parlor, saloon owner	Choice	दुकान, पार्लर, सैलून आदि के मालिक
Driver-Taxi/Auto/Cab/Bus	Choice	**ड्राइवर** *[टैक्सी / ऑटो / टैक्सी / बस]*
labour etc.	Choice	घर नौकरानी, सफाई कर्मचारी, निर्माण या कृषि श्रमिक आदि
executive, private job etc.]	Choice	निजी नौकरी आदि]*
Salaried job, consultant, executive, doctor, nurse	Choice	वेतनभोगी नौकरी, परामर्शदाता, अधिकारी, डॉक्टर, नर्स
Other	Choice	अन्य
**Husband/ Partner's information**		**पति/साथी**
**How old is (was) your husband/parner?**	Integer	**आपके पति/साथी की उम्र कितनी है (थी)?**
**What is the highest level of education your husband/partner has (had) completed?**		**आपके पति /साथी की उच्चतम शिक्षा क्या है (थी) ?**
No formal education	Choice	कोई औपचारिक शिक्षा नहीं
Primary (1-5th standard)	Choice	प्राइमरी (1-5th standard)
Middle (6-8th standard)	Choice	मिडिल (6-8th standard)
High school (9-10th standard)	Choice	हाई स्कूल (9-10th standard)
Senior school (11-12th standard)	Choice	सीनियर स्कूल (11-12th standard)
Undergraduate	Choice	ग्रेजुएशन पूरा नहीं
Graduate	Choice	ग्रेजुएशन
Incomplete postgraduate	Choice	पोस्ट ग्रेजुएशन पूरा नहीं
Postgraduate or higher	Choice	पोस्ट ग्रेजुएशन और आगे
Other	Choice	अन्य
Don't know	Choice	नहीं पता
**Is (was) your husband/partner working?**		**क्या आपके पति/ साथी कोई रोजगार या काम करते हैं (थे)?**
No	Choice	नहीं
Yes	Choice	हाँ
Don't Know	Choice	पता नहीं
**What kind of work does he (did he) mainly do?**		**मुख्यतः आपके पति/साथी किस प्रकार का काम/व्यवसाय करते हैं (थे)?**
**Home-based earnings** *[stitching, pani-puri making, embroidery, snacks, gota/sitara]*	Choice	**घर पर आधारित काम (आय)** [सहायता [सिलाई, पानी-पुरी बनाने, कढ़ाई, नाश्ते बनाने, गोटा / सीतारा टकिंग]
flower/garland selling, snacks selling]	Choice	बिक्री]
etc.]	Choice	**दुकान मालिक** [पार्लर, सैलून, पान, किराना, मांस इत्यादि]

For peer review only

**Driver**-Taxi/Auto/Cab/Bus etc.]	Choice	**ड्राइवर** [टैक्सी / ऑटो / टैक्सी / बस इत्यादि]
labour etc.	Choice	घर नौकरानी, सफाई कर्मचारी, निर्माण या कृषि श्रमिक इत्यादि।
executive, private job etc.]	Choice	निजी नौकरी आदि]
teacher	Choice	वेतनभोगी नौकरी, परामर्शदाता, अधिकारी, डॉक्टर, नर्स, शिक्षक
Don’t Know	Choice	पता नहीं
Other	Choice	अन्य
take drugs?**		**क्या आपके पति/साथी किसी भी तरह के मादक पदार्थ या शराब पीते है (थे)?**
No	Choice	नहीं
Yes	Choice	हाँ
Don’t Know	Choice	पता नहीं
**How often in a month does (did) he take it?**		**आपके पति/साथी एक महीने में कितनी बार नशा करते है (थे)?**
Sometimes	Choice	कभी कभी
Regularly	Choice	नियमित रूप से
Don’t Know	Choice	पता नहीं
had during your life.	Label	जीवनकाल में जन्म दिया हैं.***
**Have you ever been pregnant?**		**क्या आप कभी गर्भवती हुई हैं?**
No	Choice	नहीं
Yes	Choice	हाँ
**Have you ever given birth to a child?**		**क्या आपने कभी किसी बच्चे को जन्म दिया है?**
No	Choice	नहीं
Yes	Choice	हाँ
**Do you have any (live) children?**		**क्या आपको कोई (जीवित) बच्चे है?**
No	Choice	नहीं
Yes	Choice	हाँ
**Number of living son(s)**	Integer	**जीवित बेटों की संख्या**
**Number of living daughter(s)**	Integer	**जीवित बेटियों की संख्या**
**Household assets**		**हाउसहोल्ड की संपत्ति**
**Which of these things do you have in your home?**		**आपके घर में इनमें से कौन से समान हैं?**
Mattress	Choice	गद्दे

For peer review only

Chair	Choice	कुर्सी
Sofa-set	Choice	सोफा सेट
Cot/bed (Sofa bed)	Choice	बेड (पलंग)/सोफा बेड
Table	Choice	टेबल /मेज
Almirah	Choice	अलमारी
Pressure cooker	Choice	प्रेशर कुकर
Clock/watch	Choice	घड़ी
Metered Electricity	Choice	मीटर बिजली
Radio/transistor	Choice	रेडिओ / ट्रांजिस्टर
Electric fan	Choice	बिजली का पंखा
Cooler	Choice	कूलर
AC	Choice	ए. सी.
Normal TV	Choice	सामान्य टीवी
LED or LCD TV	Choice	एल.इ.डी या एल.सी.डी टी. वी.
Basic mobile phone	Choice	साधारण मोबाइल फ़ोन
Smart phone	Choice	स्मार्ट फ़ोन
Computer (desktop)	Choice	कम्प्यूटर
Laptop	Choice	लैपटॉप
Induction cooker	Choice	इंडक्शन कुकर
Refrigerator/fridge	Choice	फ्रिज
Mixer-grinder	Choice	मिक्सर
Water purifier	Choice	वाटर प्यूरीफायर
Washing machine	Choice	वॉशिंग मशीन
Sewing machine	Choice	सिलाई मशीन
Water-pump/motor	Choice	पानी का पंप / मोटर
Cycle	Choice	साइकल
Bike/scooter/scooty (two-wheeler)	Choice	बाइक /स्कूटर/स्कूटी/(अन्य दुपहिया)
Car	Choice	कार
Auto or taxi	Choice	रिक्शा/ टैक्सी
Geyser	Choice	ग्रीज़र

For peer review only

Tap drinking water	Choice	नल का पानी
**Do you drink alcohol or take any drug?**		**क्या आप किसी भी तरह के मादक पदार्थ या शराब पीते हैं?**
No	Choice	नहीं
Yes	Choice	हाँ
**How often in a month do you take it?**		**आप इसे महीने में कितनी बार लेते हैं ?**
Sometimes	Choice	कभी कभी
Regularly	Choice	नियमित रूप से
**PHQ9**		**अवसाद (PHQ9)**
bothered by any of the following problems?***		***पिछले 2 हफ्तों (15 दिनों) में, आपको कितनी बार निम्नलिखित समस्याओं से परेशान किया गया है?***
**Little interest or pleasure in doing things**		है.**
Not at all sure	Choice	एक भी दिन नहीं
Several days	Choice	कई दिन
Over half the days	Choice	ज्यादातर दिनों में
Nearly every day	Choice	लगभग हर रोज
**Feeling down, depressed, or hopeless**	As 171	**उदास रहना या हर वक्त निराशा में रहना.**
much**		रहना.**
**Feeling tired or having little energy**		**थकावट सी रहना या कमजोरी महसूस करना.**
**Poor appetite or overeating**		**खाने को दिल नहीं करना या जरूरत से ज्यादा खाना.**
**Feeling bad about yourself or that you are a failure or have let yourself or your family down.**		**खुद के बारे में बुरा महसूस करना या खुद को असफल समझना. या खुद को नीचा समझना क्योंकि आपने परिवार वालों की उम्मीदों पर खरे नहीं उतर पाए.**
**Trouble concentrating on things, such as reading the newspaper or watching television**		**किसी भी काम में मन नहीं लगना जैसे कि कुछ पढना, लिखना या टी.वी. देखना इत्यादि.**
could have noticed. Or the opposite being so figety or restless that you have been moving around a lot more than usual**		**इतने धीरे (अहिस्ता) चलना या इतने धीरे बातें करना कि लोग इस बात को ध्यान (नोटिस) देने लगे या फिर इस का उलट की आप हर समय बहुत जल्दी में और बेचैनी में रहते हों.**
hurting yourself.**		नुकसान पहुचने के बारे में सोचा.**
**GAD7**		**चिंता (GAD7)**
bothered by any of the following problems?***		***पिछले 2 हफ्तों (15 दिनों) में, आपको कितनी बार निम्नलिखित समस्याओं से परेशान किया गया है?***

For peer review only - http://bmjopen.bmj.com/site/about/guidelines.xhtml

**Feeling nervous, anxious, or on edge**		**घबराहट या परेशनी महसूस करना.**
Not at all sure	Choice	एक भी दिन नहीं
Several days	Choice	कई दिन
Over half the days	Choice	ज्यादातर दिनों में
Nearly every day	Choice	लगभग हर रोज
**Not being able to stop or control worrying**	As 186	**चिंता को रोक न पाना**
**Worrying too much about different things**	As 186	**हर बात के लिए ज्यादा चिंतित रहना.**
**Trouble relaxing**	As 186	**आराम न कर पाना या मुश्किल होना.**
**Being so restless that it's hard to sit still**	As 186	हों**
**Becoming easily annoyed or irritable**	As 186	**मामूली सी बात पर बेचैन या गुस्सा या नाराज हो जाना.**
**Feeling afraid as if something awful might happen**	As 186	**कोई भयकर घटना घटने का डर या भय महसूस होता हो.**
**Suicidal ideation**		**आत्महत्या करने की प्रवत्ति**
**Apart from in the last two weeks, have you ever thought about ending your life?**		**क्या आपने अपने जीवन में कभी भी आत्महत्या करने के बारे में सोचा है?**
Never	Choice	कभी नहीं
Yes, in the last 6 months	Choice	हाँ, पिछले 6 महीने में
Yes, in the last year	Choice	हाँ, पिछले एक साल में
Yes more than a year ago,	Choice	हाँ, पिछले एक साल से पहले
**Have you ever tried to take your life?**		है?**
**Emotional violence**		**भावनात्मक हिंसा**
which happen to some women. Please tell me if these apply to your relationship with your husband/partner or other family members?*		***अब मैं आपको कुछ परिस्थितियों के बारे में पूछने जा रही हूं जो कुछ महिलाओं के साथ होती हैं. कृपया मुझे बताएं कि क्या ये आपके रिश्ते पर लागू होते हैं?***
you feel bad about yourself?		आपको आपके बारे में बुरा महसूस करवाया?**
Yes	Choice	हाँ
No	Choice	नहीं
**How often has this happened to you?**		**आपके साथ ऐसा कितनी बार हुआ है?**
Once in the last 12 months	Choice	पिछले 12 महीनों मे एक बार
Sometimes in the last 12 months	Choice	पिछले 12 महीनों मे कई बार
Many times in the last 12 months	Choice	पिछले 12 महीनों मे बहुत बार

For peer review only

In the last 15 days	Choice	पिछले 15 दिनों में
None of the above	Choice	इनमें से कोई नहीं
**Who mainly did this to you?**		**आपके साथ यह करने वाला मुख्य व्यक्ति कौन था?**
Husband (Partner)	Choice	पति (साथी)
Natal family members	Choice	मायके (के लोग) वाले
Husband's family members	Choice	ससुराल (के लोग) वाले
Other (stranger, neighbour, teacher, leader, police etc)	Choice	अन्य (अजनबी, पड़ोसी, शिक्षक, नेता, पुलिस आदि)
**treated you indifferently?**	As 209	आपके साथ बत्तमीजी से बर्ताव किया?**
**something to belittle or humiliate you in front of other people?**	As 209	सामने नीचा दिखाया या आपके साथ ऐसा कुछ व्यवहार किया जिससे लोगो के सामने आपको अपमानित लगे?**
**you or intimidate you on purpose (e.g. By the way they looked at you, by yelling and smashing things)?**	As 209	जानबूज कर कुछ किया है (जैसे आपकी तरफ गुस्से से देखा या चिल्लाया या चीजो को तोडा इत्यादि)?
**you or someone you care about or take away your children?**	As 209	अपने/ नजदीकी व्यक्ति को चोट या नुकसान पहुचाने या बच्चो को आपसे दूर करने की धमकी दी?**
**Physical violence**		**शारीरिक हिंसा**
**happen to some women. Please tell me if anyone has ever done any of the following things to you***		होती हैं. कृपया मुझे बताओ कि क्या आपने कभी भी इनमें से कुछ भी आपके साथ हुआ है.***
**Has anyone in your family ever pushed you, shoved you, shaken you or done something to hurt you?**		**क्या आपके परिवार के किसी भी सदस्य नें कभी भी आपको धक्का दिया, झिंझोडा या आपकी तरफ कोई चीज़ उठाकर फेंकी ताकि आपको चोट पहुचे?**
Yes	Choice	हाँ
No	Choice	नहीं
**How often has this happened to you?**		**आपके साथ ऐसा कितनी बार हुआ है?**
Once in the last 12 months	Choice	पिछले 12 महीनों मे एक बार
Sometimes in the last 12 months	Choice	पिछले 12 महीनों मे कई बार
Many times in the last 12 months	Choice	पिछले 12 महीनों मे बहुत बार
In the last 15 days	Choice	पिछले 15 दिनों में
None of the above	Choice	इनमें से कोई नहीं
**Who mainly did this to you?**		**आपके साथ यह करने वाला मुख्य व्यक्ति कौन था?**
Husband (Partner)	Choice	पति (साथी)
Natal family members	Choice	मायके (के लोग) वाले

For peer review only

For peer review only - http://bmjopen.bmj.com/site/about/guidelines.xhtml

Husband's family members	Choice	ससुराल (के लोग) वाले
Other (stranger, neighbour, teacher, leader, police etc)	Choice	अन्य (अजनबी, पड़ोसी, शिक्षक, नेता, पुलिस आदि)
**Has anyone in your family ever twisted your arm, banged your head or pulled your hair?**	As 226	**क्या आपके परिवार के किसी भी सदस्य नें कभी भी आपकी बांह मरोड़ी या आपका सिर पटका या आपके बाल खींचे?**
bitten you?**	As 226	चिमटी काटा या दांतों से काटा?**
**Has anyone in your family ever hit or punched you with their fist or something else that could hurt you?**	As 226	**क्या आपके परिवार के किसी भी सदस्य नें कभी भी, आपको मुक्के मारे य़ा किसी चीज़ से मारा ज़िससे आपको चोट लग सके?**
you or beaten you up?**	As 226	घसीटा या आपको पीटा है?**
or burned you with a cigarette/Bidi, kerosene, chemicals, acid?**	As 226	या आप पर सिगरेट / बीड़ी, मिट्टी का तेल, रसायन, एसिड का उपयोग कर जलाया?**
sharp object such as broken glass, a razor blade, axe, or knife or used any instruments or weapons to threaten or harm you?**	As 226	**क्या आपके परिवार के किसी भी सदस्य नें कभी भी, आप पर किसी तेज़ धार वाले साधन या हथियार जैसे टूटे कांच, रेजर, ब्लेड, कुल्हाड़ी, चाकू इत्यादि से हमला किया या करने की धमकी दी?**
threatened you with a blunt object such as a belt, stone, broomstick, or rolling pin?**	As 226	**क्या आपके परिवार के किसी भी सदस्य नें कभी भी, आपको बेल्ट, पत्थर, झाड़ू या बेलन जैसी या किसी भारी वस्तु से मारा है, या मारने की धमकी दी?**
suffocate, choke, hang you, or poison you on purpose?**	As 226	**क्या आपके परिवार के किसी भी सदस्य नें कभी भी, आपका दम या गला घोंटने या आपको फांसी लगाने या जहर देने की कोशिश की?**
**Sexual violence**		**लैंगिक हिंसा**
happen to some women. Please tell me if anyone has ever done any of the following things to you.***		साथ होती है. ऑप मुझे बताये की इनमे ने कोई भी बात ऑप कें साथ किसी ने की है?***
you to have sexual intercourse with him *even when you did not want to*?**		शारीरिक बल के प्रयोग से आपके साथ सोने (सेक्स/ यौन-संबंध) के लिए ऑपको मजबूर किया?**
Yes	Choice	हाँ
No	Choice	नहीं
**How often has this happened to you?**		**आपके साथ ऐसा कितनी बार हुआ है?**
Once in the last 12 months	Choice	पिछले 12 महीनों मे एक बार
Sometimes in the last 12 months	Choice	पिछले 12 महीनों मे कई बार
Many times in the last 12 months	Choice	पिछले 12 महीनों मे बहुत बार
In the last 15 days	Choice	पिछले 15 दिनों में
None of the above	Choice	इनमें से कोई नहीं

For peer review only

**Who mainly did this to you?**		**आपके साथ यह करने वाला मुख्य व्यक्ति कौन था?**
Husband (Partner)	Choice	पति (साथी)
Natal family members	Choice	मायके (के लोग) वाले
Husband's family members	Choice	ससुराल (के लोग) वाले
Other (stranger, neighbour, teacher, leader, police etc)	Choice	अन्य (अजनबी, पड़ोसी, शिक्षक, नेता, पुलिस आदि)
**Has anyone ever physically forced you to perform any other sexual acts *even you did not want to*?**	As 250	शारीरिक बल से आपको सेक्स/यौन से सम्बंधित कोई भी हरकत (क्रिया) करने को मजबूर किया?**
**other way to perform sexual acts *when you did not want to*?**	As 250	डरा-धमका कर या कोई और तरीके से सेक्स/यौन सम्बन्धी क्रिया (हरकत) करने के को मजबूर किया?**
**replicate a sexual behaviour from pornography or other sexual material *against your will*?**	As 250	अश्लील साहित्य या अन्य यौन सामग्री दिखाकर आपको वैसे ही यौन व्यवहार (हरकत) को दोहराने के लिए मजबूर किया?**

For peer review only

For peer review only - http://bmjopen.bmj.com/site/about/guidelines.xhtml

STROBE Statement—Checklist of items that should be included in reports of ***cross-sectional studies***

	Item No	Recommendation	Page No
**Title and abstract**	1	(*a*) Indicate the study's design with a commonly used term in the title or the abstract	2
		(*b*) Provide in the abstract an informative and balanced summary of what was done and what was found	2
**Introduction**			
Background/rationale	2	Explain the scientific background and rationale for the investigation being reported	4-6
Objectives	3	State specific objectives, including any prespecified hypotheses	7
**Methods**			
Study design	4	Present key elements of study design early in the paper	7
Setting	5	Describe the setting, locations, and relevant dates, including periods of recruitment, exposure, follow-up, and data collection	7
Participants	6	(*a*) Give the eligibility criteria, and the sources and methods of selection of participants	8
Variables	7	Clearly define all outcomes, exposures, predictors, potential confounders, and effect modifiers. Give diagnostic criteria, if applicable	9-11
Data sources/ measurement	8*	For each variable of interest, give sources of data and details of methods of assessment (measurement). Describe comparability of assessment methods if there is more than one group	*8*
Bias	9	Describe any efforts to address potential sources of bias	12
Study size	10	Explain how the study size was arrived at	8
Quantitative variables	11	Explain how quantitative variables were handled in the analyses. If applicable, describe which groupings were chosen and why	10
Statistical methods	12	(*a*) Describe all statistical methods, including those used to control for confounding	11
		(*b*) Describe any methods used to examine subgroups and interactions	Not Applicable
		(*c*) Explain how missing data were addressed	11
		(*d*) If applicable, describe analytical methods taking account of sampling strategy	Not Applicable
		(*e*) Describe any sensitivity analyses	11
**Results**			
Participants	13*	(a) Report numbers of individuals at each stage of study—eg numbers potentially eligible, examined for eligibility, confirmed eligible, included in the study, completing follow-up, and analysed	13
		(b) Give reasons for non-participation at each stage	12
		(c) Consider use of a flow diagram	Not Applicable
Descriptive data	14*	(a) Give characteristics of study participants (eg demographic, clinical, social) and information on exposures and potential confounders	13-14
		(b) Indicate number of participants with missing data for each variable of interest	11

For peer review only

Outcome data	15*	Report numbers of outcome events or summary measures	15-20
Main results	16	(*a*) Give unadjusted estimates and, if applicable, confounder-adjusted estimates and their precision (eg, 95% confidence interval). Make clear which confounders were adjusted for and why they were included	15-20
		(*b*) Report category boundaries when continuous variables were categorized	11
		(*c*) If relevant, consider translating estimates of relative risk into absolute risk for a meaningful time period	Not Applicable
Other analyses	17	Report other analyses done—eg analyses of subgroups and interactions, and sensitivity analyses	Not Applicable
**Discussion**			
Key results	18	Summarise key results with reference to study objectives	21
Limitations	19	Discuss limitations of the study, taking into account sources of potential bias or imprecision. Discuss both direction and magnitude of any potential bias	25
Interpretation	20	Give a cautious overall interpretation of results considering objectives, limitations, multiplicity of analyses, results from similar studies, and other relevant evidence	21-23
Generalisability	21	Discuss the generalisability (external validity) of the study results	21-23
**Other information**			
Funding	22	Give the source of funding and the role of the funders for the present study and, if applicable, for the original study on which the present article is based	27

*Give information separately for exposed and unexposed groups.

Note: An Explanation and Elaboration article discusses each checklist item and gives methodological background and published examples of transparent reporting. The STROBE checklist is best used in conjunction with this article (freely available on the Web sites of PLoS Medicine at http://www.plosmedicine.org/, Annals of Internal Medicine at http://www.annals.org/, and Epidemiology at http://www.epidem.com/). Information on the STROBE Initiative is available at www.strobe-statement.org.

For peer review only

For peer review only - http://bmjopen.bmj.com/site/about/guidelines.xhtml
